# Developing Growth Reference Curves for Jordanian Children and Adolescents: A Comparative Analysis with WHO Growth Standards and References

**DOI:** 10.3390/healthcare14182898

**Published:** 2026-09-08

**Authors:** Walid Al-Qerem, Ruba Zumot, Anan Jarab, Judith Eberhardt, Alaa Hammad, Fawaz Alasmari, Nouf Alsultan

**Affiliations:** 1 Department of Pharmacy, Faculty of Pharmacy, Al-Zaytoonah University of Jordan, Amman 11733, Jordan; 202127091@std-zuj.edu.jo (R.Z.); alaa.hammad@zuj.edu.jo (A.H.); 2 Department of Clinical Pharmacy, Faculty of Pharmacy, Jordan University of Science and Technology, Irbid 22110, Jordan; asjarab@just.edu.jo; 3 Department of Psychology, School of Social Sciences, Humanities and Law, Teesside University, Borough Road, Middlesbrough TS1 3BX, UK; j.eberhardt@tees.ac.uk; 4 Department of Pharmacology and Toxicology, College of Pharmacy, King Saud University, Riyadh 4545, Saudi Arabia; ffalasmari@ksu.edu.sa; 5 Faculty of Medicine and Health Sciences, Medical School, University of Nottingham, Nottingham NG7 2QL, UK; alxna75@nottingham.ac.uk

**Keywords:** BMI-for-age, growth reference, height-for-age, Jordan, weight-for-age

## Abstract

**Background:** Growth standards and growth references serve different purposes. The WHO Child Growth Standards for ages 0–5 years describe growth under specified optimal conditions, whereas the WHO 5–19-year curves are a reference reconstructed from historical NCHS data. This study aimed to develop sex-specific growth reference curves for Jordanian children and adolescents and compare selected centiles descriptively with WHO growth standards and references. **Methods:** Retrospective anthropometric records collected between 2019 and 2025 through the Ministry of Health’s Hakeem National E-Health Program were used. Following medical eligibility screening, 63,187 measurement records were selected before analytical cleaning using proportionate stratified random sampling by sex and governorate, with age balancing across four broad age groups. Analytical cleaning excluded 506 boys’ and 1084 girls’ records, producing a final height-for-age and BMI-for-age sample of 61,597 measurement occasions from 33,170 Jordanian children and adolescents (27,933 occasions from 14,594 boys and 33,664 occasions from 18,576 girls). Weight-for-age used 16,071 occasions from 9537 boys and 16,185 occasions from 10,214 girls aged 2–10 years. LMS/BCPE-based centile modeling was conducted within GAMLSS, and the 3rd, 15th, 50th, 85th, and 97th centiles were compared with WHO values. **Results:** Separate sex-specific models were developed, and the Jordanian curves differed descriptively from WHO curves at several ages and centiles. **Conclusions:** The population-proportionately stratified Jordanian curves provide direct local context for describing attained anthropometric distributions. Future studies should compare both the Jordanian and WHO curves against Jordan-specific outcomes and classification decisions to determine their respective clinical roles.

## 1. Introduction

Comparing a child’s anthropometric measurements, such as weight, head circumference, and height, to standardized growth charts for the same age and sex is crucial for assessing healthy growth and identifying deviations from normal development. Weight, which readily reflects acute changes, is the most widely used of these measurements. Meanwhile, height is a critical indicator for diagnosing and potentially preventing early-life stunting [1].

Numerous countries use the World Health Organization (WHO) and the Centers for Disease Control and Prevention (CDC) international growth references or standards for monitoring child growth despite variations in ethnicity and socioeconomic factors [2]. The WHO Child Growth Standards for ages 0–5 years are prescriptive standards describing growth under specified optimal environmental conditions, whereas the WHO 5–19-year curves are a growth reference derived from observed historical data. Population-derived curves based on routine healthcare records, such as those developed in the present study, are appropriately interpreted as growth references rather than prescriptive standards. Differences between populations may reflect genetic, nutritional, socioeconomic, environmental, and healthcare-related factors, but observed differences do not establish the clinical superiority of either a local or an international reference. Some countries have developed their own population-specific growth standards. For instance, the United Kingdom has integrated WHO growth standards with national data to create tailored UK–WHO growth charts that reflect its population’s genetic, socioeconomic, and cultural backgrounds [3]. Countries such as China, Bolivia, Denmark, Norway, and Belgium have not extensively utilized the WHO growth charts, citing discrepancies between the growth trajectories of their populations and those outlined in the standard growth charts [4]. The WHO Child Growth Standards describe growth under specified optimal conditions, whereas the WHO 5–19 and CDC charts are descriptive references; social, economic, and environmental conditions may affect growth and development across regions [5].

Anthropometric percentiles are influenced by external factors such as environment, country of origin, and ethnicity [6]. Therefore, tailoring these parameters to each nation, by sex and age, is crucial. Growth-reference data are useful for monitoring growth and development, as they assist healthcare providers in diagnosing and managing metabolic and growth-related issues, such as poor nutritional status and obesity [7].

In Jordan, WHO and CDC growth charts are commonly used by healthcare centers, pediatricians, and parents to monitor children’s growth. A previous study using anthropometric records from the Hakeem electronic health-record system evaluated the applicability of WHO and CDC references and identified systematic differences between the international and Jordanian distributions [8]. Because both studies drew records from Hakeem, some row-level overlap is possible, although the exact overlap could not be quantified from the retained study files. The earlier study evaluated the applicability of international references; it did not derive the sex-specific Jordanian GAMLSS height-for-age, weight-for-age, and BMI-for-age models reported here. The present study therefore aimed to derive sex-specific Jordanian growth reference curves and to compare selected centiles descriptively with WHO growth standards and references.

## 2. Materials and Methods

This multicenter retrospective study analyzed records of Jordanian children and adolescents collected between 2019 and 2025. All eligible measurements recorded during this period were pooled to estimate age- and sex-specific attained anthropometric distributions. Data extraction and initial statistical analysis were conducted between March and November 2025. The data were retrieved from the Ministry of Health’s Hakeem National E-Health Program (Electronic Health Solutions, Amman, Jordan) and comprised cross-sectional observations and repeated measurement occasions from participating governmental hospitals and polyclinics. Ethical approval was granted by the research ethics committees of Al-Zaytoonah University of Jordan (IRB #2025-2024/12/30; approved on 30 December 2024) and the Jordanian Ministry of Health (REF#2025/2-02/531/2024; approved on 22 March 2025). Parents or legal guardians had granted permission to the participating healthcare facilities and the Hakeem program for the research use of their children’s routinely collected health records. The study involved retrospective analysis of de-identified records without direct participant contact or additional data collection. Reporting was guided by the RECORD statement for observational studies using routinely collected health data [9].

Anthropometric measurements were obtained during routine clinical encounters at Ministry of Health hospitals and polyclinics participating in the Hakeem National E-Health Program between 2019 and 2025. Measurements were performed by qualified and professionally licensed nurses working under Ministry of Health procedures aligned with internationally accepted anthropometric practice, including ISAK recommendations [10]. Footwear and heavy outer clothing were removed before measurement. Height and weight were recorded in centimeters and kilograms, respectively, and BMI was calculated as weight in kilograms divided by height in meters squared. Because this was a retrospective multisite electronic health-record study, measurements were not obtained using a single study-specific instrument; device manufacturers’ names and locations, device models, and facility-specific calibration records were not included in the analytical extract.

Eligible participants were Jordanian children and adolescents aged 2–18 years. Eligibility screening used the diagnosis and medication fields available in the Hakeem extract. Records were excluded when they contained any prespecified condition, treatment, or medication expected to materially affect growth, as listed in Table 1. The screening relied on routinely recorded electronic information rather than an independent clinical assessment; conditions not documented in the available record could not be assessed. A recorded clinical diagnosis of childhood obesity was exclusionary, whereas BMI above the 95th percentile alone was not. The underlying coding dictionary and row-level clinical records are confidential and are not reproduced.

Records were processed in three sequential stages. First, the prespecified medical eligibility criteria were applied. Second, 63,187 eligible measurement records were selected before analytical cleaning through proportionate stratified random sampling in R, comprising 28,439 boys’ and 34,748 girls’ records. Because the growth models were fitted separately by sex, the sampling totals were defined independently for boys and girls, and governorate proportionality was applied within each sex. Separate strata were defined by sex, governorate, and broad age group (2.00 to <6.00, 6.00 to <10.00, 10.00 to <14.00, and 14.00 to 18.00 years). Within each sex, governorate quotas were proportional to the estimated distribution of Jordanian children aged 2–18 years across all 12 governorates. The rounded governorate percentages summed to 100.6%; therefore, they were normalized to 100% before allocation, and integer quotas were assigned using the largest-remainder method. The sex–governorate quotas were then distributed across the broad age groups to maintain coverage throughout the modeled age range, and records were randomly selected within each joint stratum. Third, the selected records underwent analytical cleaning. This procedure aligned the governorate distribution within each sex with the national target distribution while maintaining age coverage.

Analytical cleaning produced 61,597 height-for-age and BMI-for-age measurement occasions from 33,170 unique children: 27,933 occasions from 14,594 boys and 33,664 occasions from 18,576 girls. Among boys, 5266 children (36.1%) contributed more than one measurement; among girls, 5965 (32.1%) did so. The median number of measurements per child was 1 (interquartile range 1–2) in both sexes. Repeated observations were identified using the anonymized child identifier. Exact duplicates were defined by child identifier, age in days, modeled height, and modeled weight; none remained in the final height-for-age/BMI-for-age frame. All retained, genuinely distinct measurement occasions were treated as independent contributions to the age-specific attained-growth distributions in the GAMLSS centile models; no patient-level random effect, clustering term, or cluster-adjusted uncertainty estimate was applied. This decision follows an established WHO methodological precedent for attained-centile construction. The WHO Multicentre Growth Reference Study combined a longitudinal component containing repeated measurements from birth to 24 months with a cross-sectional component at older ages, and its attained-growth methodology addressed the use of longitudinal and cross-sectional data. The WHO 5–19-year reference subsequently applied the same BCPE/GAMLSS statistical framework used to construct the WHO Child Growth Standards [11,12,13]. Because repeated measurements in the present study arose from routine clinical encounters rather than scheduled research follow-up, the numbers of unique children and measurement occasions and the distribution of repeated contributions are reported separately in Table 2 and Supplementary Table S1.

After sampling, records were screened for missing or invalid values, exact duplicates, impossible combinations, and internally inconsistent anthropometric measurements. Age- and sex-specific *z* scores for height-for-age, weight-for-age, and BMI-for-age were calculated in accordance with WHO growth-reference methodology, and observations beyond ±5 SD were excluded on an outcome-specific basis [2]. The full-age cleaning stage excluded 506 boys’ records (1.78%) and 1084 girls’ records (3.12%), corresponding to 1590 records overall (2.52%), and yielded the final 61,597-record height-for-age/BMI-for-age frame. These criteria were used as plausibility screens to limit the influence of invalid or internally inconsistent records rather than to characterize every extreme measurement as erroneous. Initial statistical analysis and modeling were completed using IBM SPSS Statistics version 26.0 (IBM Corp., Armonk, NY, USA) [14] and R version 4.5.2 (R Foundation for Statistical Computing, Vienna, Austria) [15]. R analyses were conducted within RStudio version 2023.06.2+561 (Posit Software, PBC, Boston, MA, USA) [16]. GAMLSS with the Box–Cox power exponential distribution and LMS-related centile methodology was used as a conditional distributional framework to derive smooth centile curves [17,18]. The conventional LMS/BCCG formulation models location, scale, and skewness, whereas BCPE includes a fourth parameter for kurtosis; BCCG/LMS is the special case of BCPE in which tau is fixed at 2. The four parameters were mu (μ), the conditional location or median; sigma (σ), the scale parameter; nu (ν), which governs skewness; and tau (τ), which controls kurtosis. Age was transformed as age*^p^*, where *p* denotes the age-transformation exponent and is distinct from ν. Outcome-specific age–power transformations were evaluated, and model adequacy was assessed using global and age-group Q statistics, residual distributions, worm plots, and empirical-versus-fitted centile comparisons. Subsequent validation of final model objects and publication outputs was performed in 2026 using R version 4.5.3 and the gamlss (version 5.5-0) and gamlss.dist (version 6.1-1) packages (Mikis Stasinopoulos and Robert Rigby, University of Greenwich, London, UK).

For each sex and anthropometric indicator, dozens of candidate GAMLSS models were evaluated through a sequential model-selection procedure. Alternative age transformations and spline complexities were assessed for the location (mu), scale (sigma), skewness (nu), and kurtosis (tau) parameters. Model selection was guided primarily by the generalized Akaike information criterion (GAIC), with preference given to the more parsimonious specification when competing models differed by less than two GAIC units. Convergence, residual diagnostics, Q statistics, worm plots, and the numerical validity and ordering of the fitted centile curves were also examined before selecting the final model. Model parameters and centiles are reported in Tables S2–S13, detailed age-group Q statistics in Tables S14–S19, the specifications and fit statistics of the six selected models in Table S20, the six-model diagnostic summary in Table S21, and empirical-versus-fitted centile comparisons in Table S22. Fitted percentiles were evaluated for height-for-age and BMI-for-age across ages 2–18 years and for weight-for-age across ages 2–10 years. The 3rd, 15th, 50th, 85th, and 97th centiles were selected to provide a balanced description of the lower, intermediate, median, and upper portions of the fitted distributions and to permit direct comparison with the corresponding WHO centiles. These centiles were treated as descriptive reference points rather than prescriptive clinical thresholds.

Official WHO 2006 daily expanded percentile tables were used through age day 1856. From day 1857 onward, official WHO 2007 monthly LMS tables were used, with age in months calculated as age in days divided by 30.4375 and linear interpolation between the bracketing integer months. No additional smoothing was applied at the source-defined transition. Height-for-age and BMI-for-age comparisons covered ages 2–18 years, whereas the weight-for-age comparison ended at age 10 years. Signed differences were calculated as the Jordanian centile minus the WHO centile. Numerical selected-age differences are reported in Table S23; confidence intervals, reclassification, concordance, and outcome-based clinical validation were not estimated.

## 3. Results

### 3.1. Sex-Specific Growth-Reference Models and Analytic Denominators

**Figure 1:**
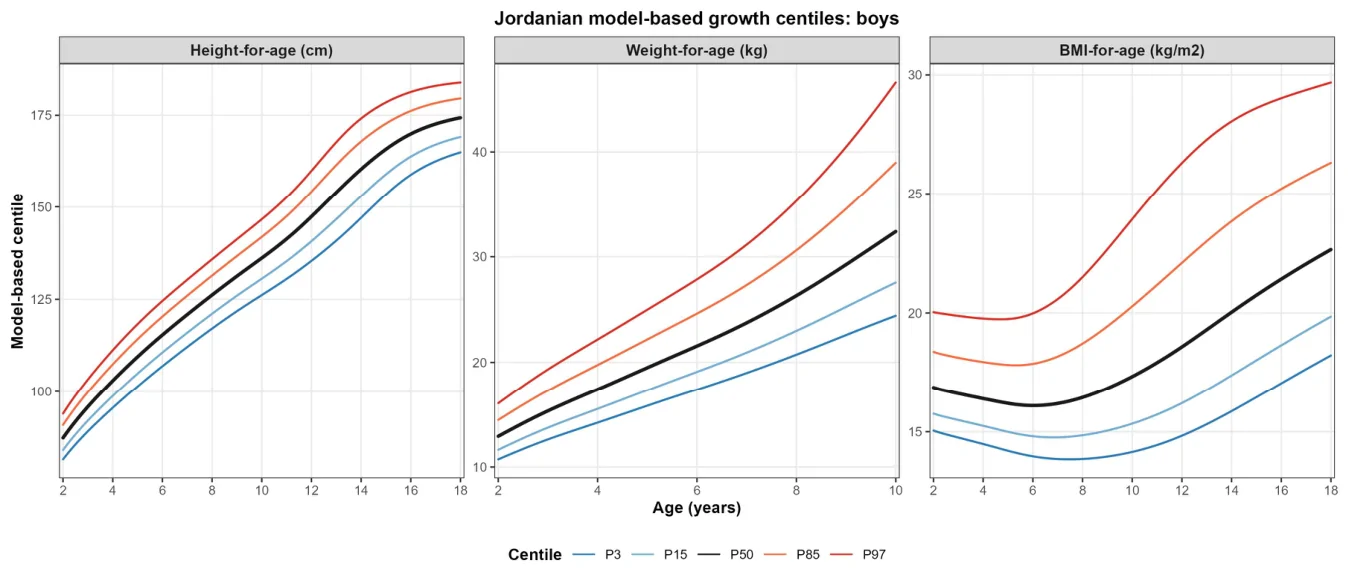
Jordanian height-for-age and BMI-for-age centiles from ages 2 to 18 years and weight-for-age centiles from ages 2 to 10 years in boys. Curves show the 3rd, 15th, 50th, 85th, and 97th centiles.

**Figure 2:**
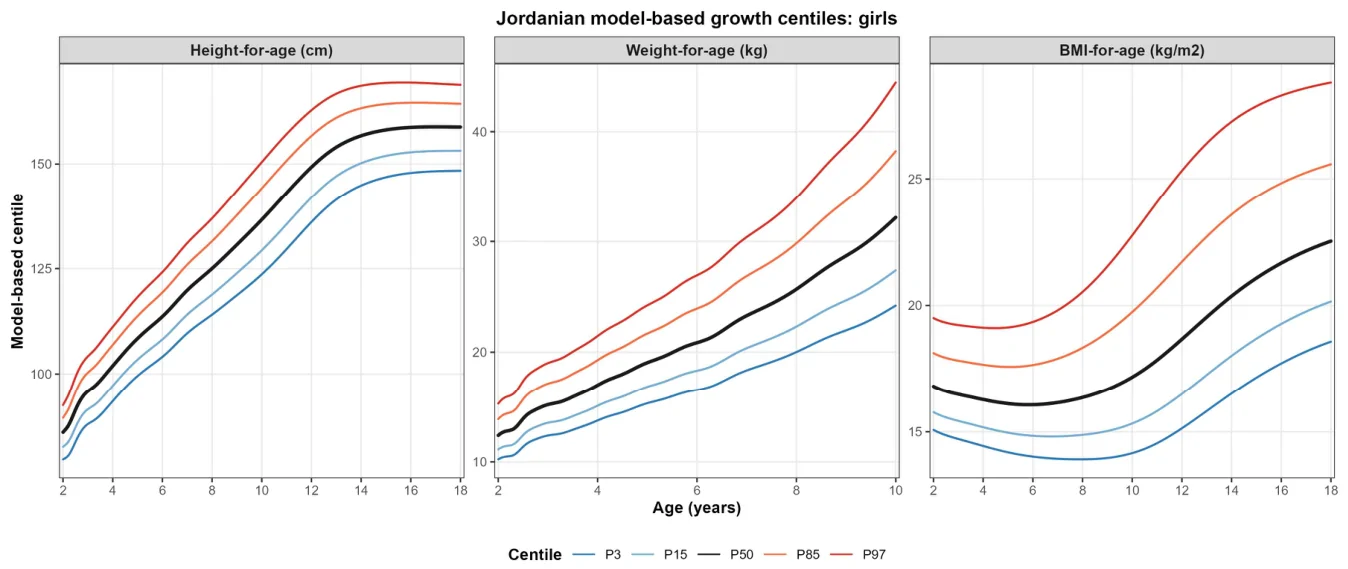
Jordanian height-for-age and BMI-for-age centiles from ages 2 to 18 years and weight-for-age centiles from ages 2 to 10 years in girls. Curves show the 3rd, 15th, 50th, 85th, and 97th centiles.

After eligibility assessment, the proportionate stratified sampling stage comprised 63,187 measurement records (28,439 boys and 34,748 girls), with the governorate distribution reported in Table 3. Analytical cleaning removed 506 boys’ and 1084 girls’ records and produced a final height-for-age and BMI-for-age sample of 61,597 measurement occasions from 33,170 children: 27,933 occasions from 14,594 boys and 33,664 occasions from 18,576 girls. The weight-for-age models used 16,071 occasions from 9537 boys and 16,185 occasions from 10,214 girls aged 2–10 years. The repeated-measurement structure is reported in Table 2 and Table S1, and the complete sampling and analytical flow is presented in Figure S1. Separate sex-specific BCPE models were fitted for height-for-age and BMI-for-age from ages 2 to 18 years and weight-for-age from ages 2 to 10 years. Model parameters and centiles are reported in Tables S2–S13, detailed Q statistics in Tables S14–S19, and selected-model specifications in Table S20. The resulting growth-reference curves for boys and girls are presented in Figure 1 and Figure 2, respectively.

All six selected models converged and met the prespecified global Q-statistic criteria; the global *p* values for Z1–Z4 and D’Agostino K^2^ were at least 0.05 for every model. Isolated local Q-statistic signals were observed in the boys’ weight-for-age and BMI-for-age models and the girls’ weight-for-age and BMI-for-age models, but none was accompanied by global lack of fit. Residual distributions, worm plots, centile geometry, and empirical-versus-fitted centile comparisons supported the retention of all six models. Summary diagnostics are reported in Table S21, Figures S2 and S3, with age-group empirical-versus-fitted comparisons in Table S22.

### 3.2. Comparison of Jordanian Centiles with WHO Growth Standards and References

**Figure 3:**
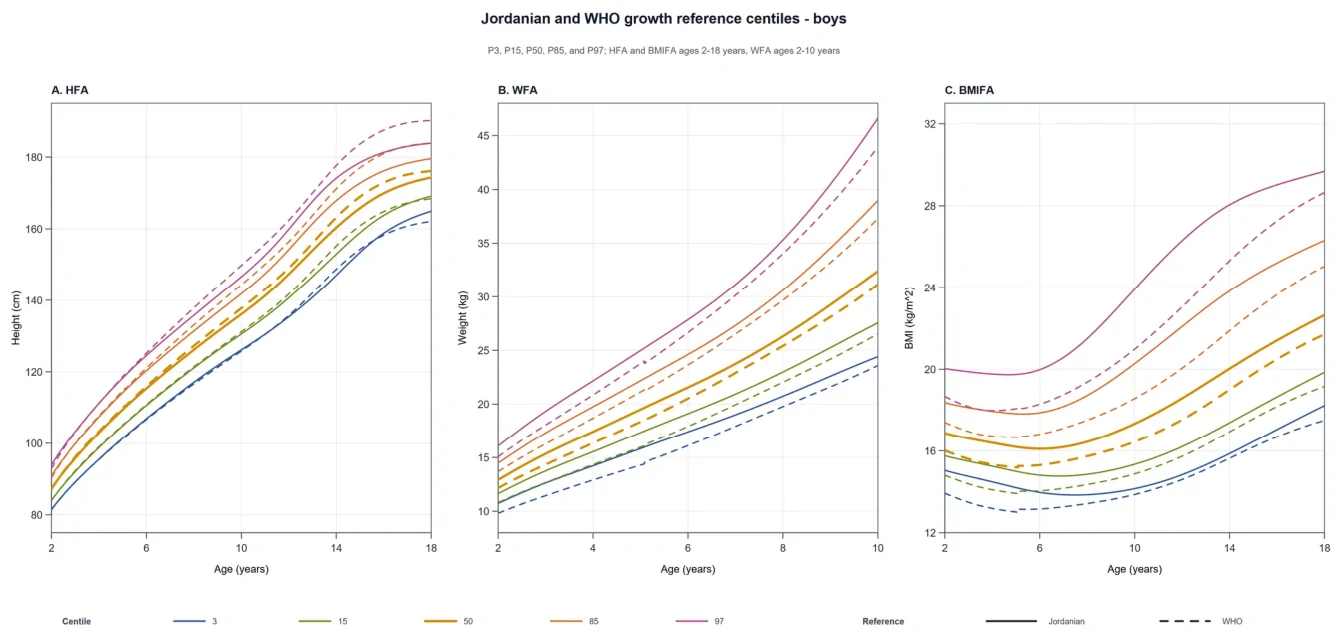
Comparison of Jordanian (solid) and WHO (dashed) centiles in boys. Height-for-age and BMI-for-age are shown from ages 2 to 18 years; weight-for-age is shown from ages 2 to 10 years. The overlays are descriptive and do not establish diagnostic superiority.

The Jordanian height-for-age percentile curves for males were closely aligned with the WHO curves at younger ages. From around age 9, a clear divergence emerged, with the Jordanian percentiles falling below the WHO reference, indicating shorter stature among Jordanian males. Within the 2–10-year comparison domain, the Jordanian weight-for-age percentiles were consistently higher than the WHO percentiles across most ages; however, weight-for-age becomes difficult to interpret during pubertal growth because weight reflects both stature and body mass. The BMI-for-age percentiles also diverged substantially from the WHO curves, with differences becoming more pronounced after age 10, particularly at the upper percentiles. These findings describe differences in BMI distributions and do not establish a higher prevalence of overweight or obesity or greater adiposity. Numerical selected-age differences are reported in Table S23 (Figure 3).

**Figure 4:**
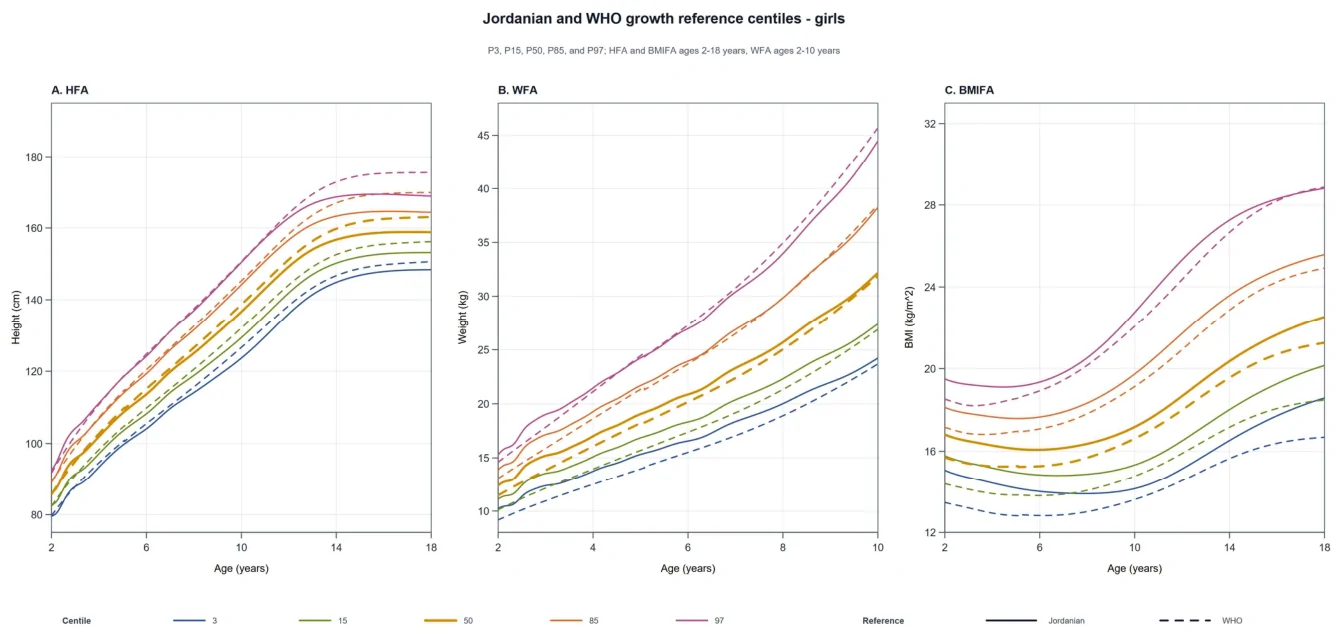
Comparison of Jordanian (solid) and WHO (dashed) centiles in girls. Height-for-age and BMI-for-age are shown from ages 2 to 18 years; weight-for-age is shown from ages 2 to 10 years. The overlays are descriptive and do not establish diagnostic superiority.

For Jordanian females, the height-for-age percentile curves showed a similar pattern to males, with the Jordanian percentiles lying below the WHO reference across most older ages. Within the 2–10-year comparison domain, the Jordanian weight-for-age percentiles were generally higher than the WHO percentiles outside the ages in which the curves overlapped. The BMI-for-age percentiles also showed consistent differences from the WHO curves, with higher BMI values observed among Jordanian females. These findings indicate differences in BMI distributions rather than a directly measured prevalence of increased adiposity. Numerical selected-age differences are reported in Table S23 (Figure 4).

## 4. Discussion

The present study generated sex-specific growth reference curves for height-for-age and BMI-for-age from ages 2 to 18 years and weight-for-age from ages 2 to 10 years using a large, multicenter, proportionately stratified sample of Jordanian children and adolescents. Governorate allocation was matched to the national target distribution within each sex, with broad age balancing across the full modeled range. The final frame comprised 61,597 measurement occasions from 33,170 children and covered all 12 governorates. Using BCPE/LMS-based centile modeling, the study provides a direct Jordanian description of attained anthropometric distributions and compares selected centiles with WHO growth standards and references. The observed differences from the WHO curves describe differences between reference distributions and do not, by themselves, determine the clinical validity of either approach. The 3rd, 15th, 50th, 85th, and 97th centiles are presented as descriptive reference points rather than prescriptive diagnostic thresholds [19].

Anthropometric measures reflect the combined influence of genetic, nutritional, socioeconomic, environmental, and healthcare-related factors, all of which play an important role in shaping child and adolescent growth. While some studies have suggested that genetic factors may have a relatively modest influence in early childhood [20,21], ethnic and population-level variability in growth patterns remains well documented [22]. In addition, environmental influences such as nutritional status, recurrent illness, physical inactivity, and broader lifestyle factors can substantially affect growth trajectories, particularly in low- and middle-income settings [23]. In Jordan, rising rates of overweight and obesity have been linked to dietary transitions toward energy-dense, processed foods, reduced fruit and vegetable intake, and increasingly sedentary lifestyles [24,25]. Similar patterns have been observed in neighboring countries, where improvements in socioeconomic conditions have coincided with shifts in nutritional status and body composition [26].

With respect to linear growth, the height-for-age centiles in the Jordanian reference closely aligned with the WHO values at younger ages but fell below the WHO centiles during later childhood and adolescence. The early-age agreement provides direct evidence of concordance in Jordanian children rather than making a locally derived reference redundant, while the later divergence documents population-specific differences in attained height. Neither pattern establishes pathological growth, population-wide nutritional adversity, or the diagnostic superiority of either reference. Comparative performance should be evaluated against Jordan-specific clinical outcomes and classification decisions.

In contrast, the weight-for-age and BMI-for-age percentiles were consistently higher in the Jordanian growth reference than in the WHO reference/standards across most age groups and percentiles. The combination of lower height-for-age and higher weight-for-age values describes a population distribution with greater body weight relative to stature, but it does not by itself establish the prevalence of overweight or obesity or increased adiposity. This distinction is particularly important because children with a recorded diagnosis of childhood obesity were included among the exclusion criteria. BMI-for-age centiles should therefore be interpreted as descriptive distributions rather than as diagnostic cutoffs. Neither the Jordanian nor WHO curves have been validated against Jordan-specific clinical outcomes, and their comparative diagnostic performance requires external and clinical validation.

Beyond lifestyle factors, differences in growth patterns may also reflect unmeasured clinical, biological, socioeconomic, or environmental factors. These factors were not directly assessed in the present analysis and therefore cannot be established as explanations for the observed curve differences.

The present findings are consistent with evidence from other countries showing that population growth references may differ from international references. For example, a 2019 study from Iran developed BMI growth charts for children and adolescents aged 6–18 years and reported substantial differences from the WHO and CDC references [27]. The close alignment between the Jordanian and WHO curves in early childhood and the divergence observed at later ages are descriptive findings. Neither similarity nor divergence establishes the diagnostic superiority of either approach. Comparative diagnostic performance requires Jordan-specific external and clinical validation.

The Jordanian reference curves were derived exclusively from Jordanian measurements and from a sampling design that proportionately represented all 12 governorates within each sex. Jordanian children were not represented in the construction datasets for either the WHO Child Growth Standards or the WHO 5–19-year reference [2]. Accordingly, the present curves have greater direct population-context relevance for describing attained anthropometric distributions in Jordan. WHO products remain useful for international comparability and established screening frameworks, whereas the Jordanian curves provide a national descriptive benchmark across a continuous age range. These functions are complementary, and neither source should be presumed clinically superior without validation against Jordan-specific outcomes.

Similarly, a national study from the Syrian Arab Republic produced updated growth reference charts for children and adolescents aged 2–20 years using a large population sample. Those charts captured local growth patterns that differed from international references, supporting the value of population-specific descriptive curves while retaining WHO standards for younger children where appropriate [28].

Comparable findings have also been reported among Chinese American children and adolescents. When weight status was classified using Chinese national standards rather than CDC standards, higher prevalence estimates of overweight and obesity were observed, particularly among females, indicating that CDC criteria may underestimate adiposity in this population [29]. In Saudi Arabia, locally developed growth charts likewise revealed substantial deviations from CDC standards, with the potential for the overestimation of short stature and malnutrition when international standards are applied [26].

A systematic review highlighted limitations in the use of international growth charts, including those from the CDC, WHO, and International Obesity Task Force (IOTF), for assessing the nutritional status of children and adolescents aged 5 years and older. Developing universally applicable growth charts that can be used over a long period of growth while accounting for genetic, cultural, socioeconomic, and body composition diversity among multiethnic children and adolescents is challenging. For instance, studies from Middle Eastern countries such as Iran and Saudi Arabia demonstrated notable variations between WHO standards and national growth charts, leading the authors to suggest the use of the national growth charts instead of the WHO’s [4]. The differences between Jordanian growth patterns and WHO standards may reflect a combination of genetic, dietary, socioeconomic, and environmental factors. Differences in height and weight between populations are known to persist across generations, indicating a role for inherited characteristics in childhood growth [20,28,30,31]. This is one hypothesis for why Jordanian children tend to have lower height-for-age percentiles than those defined by WHO standards, a pattern also reported in other Middle Eastern populations, such as Saudi Arabia [26] and Iran [27].

At the same time, dietary shifts toward energy-dense, processed foods and reduced physical activity among Jordanian children may contribute to higher BMI-for-age percentiles, mirroring trends reported in other regions undergoing nutritional transition [24,29]. Socioeconomic inequalities, including disparities in household income, parental education, and access to healthcare, may further influence growth outcomes, particularly across urban and rural settings [4,21].

In Jordan, disparities in access to healthcare and variations in living conditions between urban and rural populations may contribute to the observed variability in growth patterns. Similar findings have been documented in developing regions where socioeconomic inequality impacts childhood malnutrition rates [4]. Environmental factors, such as prenatal exposures and early-life living conditions, also play an important role and are not accounted for in international standards [5]. These external influences may contribute to population differences and support examining local distributions, although they were not measured in the present cohort.

Population-specific growth references can be useful for describing local growth patterns and for research and population surveillance. The Jordanian curves provide greater direct population-context relevance for these descriptive purposes, while neither the Jordanian nor WHO references have been validated against Jordan-specific clinical outcomes. Additional external and clinical validation is required to establish their comparative diagnostic performance.

Anthropometric measurements were obtained during routine clinical care across multiple Ministry of Health facilities rather than under a single prospective research protocol. Measurements were performed by licensed nurses following Ministry procedures aligned with accepted anthropometric practice [10], and the analytical cleaning process removed implausible or internally inconsistent records. Device-specific calibration records and inter-observer reliability data were not included in the extract. Nevertheless, the large multisite design and the small overall proportion removed during cleaning reduce the likelihood that isolated measurement variation materially determined the fitted national centiles.

Distinct measurement occasions were retained to estimate age- and sex-specific attained anthropometric distributions rather than individual growth trajectories or growth velocity. The inclusion of repeated measurement occasions is not inherently inconsistent with established growth-reference methodology: the WHO Child Growth Standards were constructed using data that included a longitudinal component with repeated observations, and the WHO 5–19-year reference subsequently used the same BCPE/GAMLSS centile-modeling framework [11,12,13]. Nevertheless, the repeated measurements in the present study arose from routine healthcare encounters rather than a fixed research schedule, and the models did not adjust uncertainty estimates for within-child clustering. Residual within-child dependence may therefore have modestly affected the precision of the estimates. However, the median contribution was one measurement occasion per child, with an interquartile range of one to two in both sexes, and most children contributed a single occasion, indicating that repeated contributions were generally limited.

The sampling frame comprised eligible Jordanian children recorded across governmental Hakeem hospitals and polyclinics. Proportionate stratification across all 12 governorates within each sex, together with broad age balancing, aligned the selected sample with the target geographic distribution of Jordanian children aged 2–18 years. This sampling structure should be distinguished from healthcare-sector coverage: the study estimates attained distributions among Jordanian children in the Hakeem frame rather than the volume of healthcare use in individual facilities. Eligibility was determined from recorded clinical information, and conditions not documented in the available electronic record could not be independently assessed.

The principal strengths of the study are the large final analytical sample, coverage of all governorates, explicit probability sampling, separate sex-specific models, transparent sampling and analytical denominators, and extensive global and local diagnostic assessment. The resulting curves provide direct Jordanian population-context information for research and population surveillance. Future studies should evaluate both the Jordanian and WHO curves against Jordan-specific clinical outcomes and classification decisions.

## 5. Conclusions

This study generated sex-specific Jordanian growth reference curves from a large, multicenter, proportionately stratified sample spanning all 12 governorates. The height-for-age and BMI-for-age references cover ages 2–18 years, whereas weight-for-age is limited to ages 2–10 years. Because the curves were derived directly from Jordanian children and adolescents, they provide a national descriptive benchmark for attained anthropometric distributions and greater direct population-context relevance than references constructed without Jordanian data. Future studies should compare both the Jordanian and WHO curves against Jordan-specific outcomes and classification decisions to determine their respective roles in routine clinical practice.

## Figures and Tables

**Table 1:** Medical conditions used as exclusion criteria.

Category	Medical Conditions
Endocrine Disorders	Hypothyroidism
	Hyperthyroidism
	Cushing’s Syndrome
	Addison’s Disease
	Growth Hormone Deficiency
	Growth Hormone Excess (Gigantism)
Genetic Disorders	Turner Syndrome
	Down Syndrome
	Achondroplasia
	Noonan Syndrome
	Prader–Willi Syndrome
	Russell–Silver Syndrome
	Marfan Syndrome
	Klinefelter Syndrome
Chronic Diseases	Type 1 Diabetes Mellitus
	Celiac Disease
	Inflammatory Bowel Disease
	Chronic Kidney Disease
	Congenital Heart Disease
	Recorded Clinical Diagnosis of Childhood Obesity
	Cystic Fibrosis
	Chronic Malnutrition
Nutritional/Environmental	Anorexia Nervosa
	Psychosocial Short Stature
Medications/Treatments	Psychostimulants for ADHD
	Long-Term Glucocorticoid Therapy
	Atypical Antipsychotics
	Cancer Therapies (Chemotherapy and Radiation)

**Table 2:** Repeated-measurement structure in the final height-for-age/BMI-for-age analytic sample by sex.

Sex	Measurement Occasions	Unique Children	One Measurement, *n*	Multiple Measurements, *n* (%)	Occasions from Repeat Children, *n* (%)	Measurements/Child, Median (IQR)
Boys	27,933	14,594	9328	5266 (36.1)	18,605 (66.6)	1 (1–2)
Girls	33,664	18,576	12,611	5965 (32.1)	21,053 (62.5)	1 (1–2)

Note: IQR = interquartile range. Outcome-specific analytic denominators are reported in Table S1; measurements and unique children must not be summed across outcomes.

**Table 3:** Proportionate stratified sampling-stage distribution by governorate and sex.

Governorate	Boys, *n*	Girls, *n*	Total, *n*	Boys, %	Girls, %	Target Population, % (2025)
Amman	11,873	14,507	26,380	41.7	41.7	41.75
Balqa	1583	1934	3517	5.6	5.6	5.57
Zarqa	4014	4905	8919	14.1	14.1	14.12
Madaba	565	691	1256	2.0	2.0	1.99
Irbid	5428	6632	12,060	19.1	19.1	19.09
Mafraq	1527	1865	3392	5.4	5.4	5.37
Jerash	792	967	1759	2.8	2.8	2.78
Ajloun	452	553	1005	1.6	1.6	1.59
Karak	848	1036	1884	3.0	3.0	2.98
Tafila	311	380	691	1.1	1.1	1.09
Ma’an	537	656	1193	1.9	1.9	1.89
Aqaba	509	622	1131	1.8	1.8	1.79
Total	28,439	34,748	63,187	100.0	100.0	100.00

Note: Counts represent the stratified sampling stage before analytical cleaning (28,439 boys and 34,748 girls; 63,187 measurement records). Governorate quotas were proportional to the distribution of Jordanian children aged 2–18 years within each sex. Because the rounded target-population percentages summed to 100.6%, they were normalized to 100% before allocation; integer counts were assigned using the largest-remainder method. Final analytical and model-specific denominators are reported separately. Population distribution source: Jordan Department of Statistics population estimates for Jordanian children aged 2–18 years by governorate, 2025 (official portal: https://dosweb.dos.gov.jo/; accessed on 8 June 2026).

## Data Availability

A disclosure-reviewed, nonconfidential reproducibility package is publicly available in Zenodo: Growth chart. Zenodo. https://doi.org/10.5281/zenodo.22007492. The package contains aggregate sex-specific parameter and centile tables, selected-model specifications, diagnostic summaries, figure source data, WHO source documentation, WHO comparison data and reproduction codes, a README, and a data dictionary. Individual-level Hakeem records, confidential coding information, unsanitized fitted model objects, and other restricted source data are not publicly shared; qualified researchers may seek access through the relevant Ministry of Health, Hakeem, institutional, and ethics procedures.

## Supplementary Materials

The following supporting information can be downloaded at: https://www.mdpi.com/article/10.3390/healthcare14182898/s1.

## Abbreviations

The following abbreviations are used in this manuscript:

WHO	World Health Organization
CDC	Centers for Disease Control and Prevention
GAMLSS	Generalized Additive Model for Location, Scale, and Shape
LMS	Lambda, mu, sigma
BCPE	Box–Cox power exponential distribution
GAIC	Generalized Akaike Information Criterion
