# Supplementary material for: Developing Growth Reference Curves for Jordanian Children and Adolescents: A Comparative Analysis with WHO Growth Standards and References
Source: Healthcare (Basel). 2026 Sep 8;14(18):2898. doi: 10.3390/healthcare14182898 (PMC13606406; doi:10.3390/healthcare14182898)

Table S1. Analytic denominators and repeated-measurement structure by sex and outcome.

Sex / analysis	Age (years)	Measurements	Unique children	Children with >1 measurement, *n* (%)	Records from repeat children, *n* (%)	Measurements/child, median (Q1-Q3)
Boys full-age analytic frame	2-18	27,933	14,594	5,266 (36.1%)	18,605 (66.6%)	1 (1-2)
Girls full-age analytic frame	2-18	33,664	18,576	5,965 (32.1%)	21,053 (62.5%)	1 (1-2)
Boys HFA model	2-18	27,933	14,594	-	-	-
Boys WFA model	2-10	16,071	9,537	-	-	-
Boys BMIFA model	2-18	27,933	14,594	-	-	-
Girls HFA model	2-18	33,664	18,576	-	-	-
Girls WFA model	2-10	16,185	10,214	-	-	-
Girls BMIFA model	2-18	33,664	18,576	-	-	-

Note. The sex-specific full-age analytic frames comprised 61,597 measurements in total (27,933 for boys and 33,664 for girls). HFA = height-for-age; WFA = weight-for-age; BMIFA = BMI-for-age; Q1-Q3 = interquartile range. Distinct repeated measurement occasions were retained. A child may contribute to more than one outcome-specific model; model denominators must not be summed.

Table S2. Height-for-age model parameters for boys aged 2-18 years (0.25-year grid).

Age (years)	L (nu)	M (mu)	S (sigma)	K (tau)
**2**	-0.7665	87.32	0.03777	2.0000
**2.25**	-0.7134	89.64	0.03811	2.0000
**2.5**	-0.6620	91.80	0.03849	2.0000
**2.75**	-0.6123	93.84	0.03888	2.0000
**3**	-0.5635	95.80	0.03927	2.0000
**3.25**	-0.5167	97.66	0.03964	2.0000
**3.5**	-0.4713	99.46	0.03997	2.0000
**3.75**	-0.4266	101.24	0.04026	2.0000
**4**	-0.3836	102.95	0.04050	2.0000
**4.25**	-0.3418	104.63	0.04069	2.0000
**4.5**	-0.3007	106.28	0.04083	2.0000
**4.75**	-0.2612	107.88	0.04092	2.0000
**5**	-0.2227	109.44	0.04096	2.0000
**5.25**	-0.1849	110.98	0.04094	2.0000
**5.5**	-0.1485	112.47	0.04087	2.0000
**5.75**	-0.1131	113.93	0.04076	2.0000
**6**	-0.0787	115.36	0.04062	2.0000
**6.25**	-0.0449	116.77	0.04045	2.0000
**6.5**	-0.0125	118.15	0.04028	2.0000
**6.75**	0.0190	119.52	0.04010	2.0000
**7**	0.0499	120.88	0.03992	2.0000
**7.25**	0.0795	122.21	0.03976	2.0000
**7.5**	0.1082	123.53	0.03962	2.0000
**7.75**	0.1363	124.86	0.03950	2.0000
**8**	0.1631	126.16	0.03940	2.0000
**8.25**	0.1891	127.44	0.03934	2.0000
**8.5**	0.2144	128.73	0.03931	2.0000
**8.75**	0.2385	129.99	0.03932	2.0000
**9**	0.2618	131.23	0.03937	2.0000
**9.25**	0.2844	132.48	0.03946	2.0000
**9.5**	0.3059	133.69	0.03960	2.0000
**9.75**	0.3265	134.90	0.03978	2.0000
**10**	0.3464	136.12	0.04002	2.0000
**10.25**	0.3656	137.37	0.04032	2.0000

Age (years)	L (nu)	M (mu)	S (sigma)	K (tau)
**10.5**	0.3839	138.63	0.04067	2.0000
**10.75**	0.4015	139.94	0.04110	2.0000
**11**	0.4186	141.31	0.04159	2.0000
**11.25**	0.4349	142.72	0.04216	2.0000
**11.5**	0.4505	144.20	0.04281	2.0000
**11.75**	0.4657	145.77	0.04353	2.0000
**12**	0.4802	147.36	0.04425	2.0000
**12.25**	0.4942	148.99	0.04494	2.0000
**12.5**	0.5078	150.66	0.04555	2.0000
**12.75**	0.5207	152.32	0.04603	2.0000
**13**	0.5332	153.98	0.04633	2.0000
**13.25**	0.5454	155.64	0.04640	2.0000
**13.5**	0.5570	157.24	0.04620	2.0000
**13.75**	0.5683	158.81	0.04569	2.0000
**14**	0.5792	160.33	0.04491	2.0000
**14.25**	0.5898	161.80	0.04391	2.0000
**14.5**	0.6000	163.20	0.04276	2.0000
**14.75**	0.6099	164.52	0.04151	2.0000
**15**	0.6197	165.79	0.04019	2.0000
**15.25**	0.6290	166.96	0.03888	2.0000
**15.5**	0.6381	168.04	0.03759	2.0000
**15.75**	0.6471	169.04	0.03636	2.0000
**16**	0.6557	169.93	0.03523	2.0000
**16.25**	0.6642	170.73	0.03419	2.0000
**16.5**	0.6726	171.44	0.03323	2.0000
**16.75**	0.6807	172.07	0.03235	2.0000
**17**	0.6887	172.63	0.03155	2.0000
**17.25**	0.6967	173.14	0.03080	2.0000
**17.5**	0.7045	173.58	0.03012	2.0000
**17.75**	0.7122	173.97	0.02949	2.0000
**18**	0.7198	174.33	0.02892	2.0000

Note. L = nu (skewness/Box-Cox power); M = mu (median/location); S = sigma (coefficient of variation/scale); K = tau (kurtosis/tail shape). K (tau) was fixed at 2.0000.

Table S3. Model-based height-for-age centiles for boys aged 2-18 years (0.25-year grid).

Age (years)	1st	3rd	5th	10th	15th	25th	50th	75th	85th	90th	95th	97th	99th
**2**	80.19	81.47	82.17	83.26	84.00	85.13	87.31	89.58	90.85	91.72	93.04	93.92	95.62
**2.25**	82.25	83.58	84.30	85.43	86.21	87.38	89.63	91.99	93.30	94.20	95.57	96.48	98.23
**2.5**	84.16	85.54	86.28	87.46	88.26	89.47	91.81	94.24	95.59	96.53	97.94	98.88	100.68
**2.75**	85.94	87.37	88.14	89.36	90.19	91.44	93.85	96.37	97.76	98.72	100.18	101.14	103.00
**3**	87.63	89.11	89.91	91.16	92.02	93.31	95.80	98.39	99.82	100.81	102.31	103.30	105.22
**3.25**	89.25	90.77	91.60	92.89	93.77	95.10	97.66	100.33	101.80	102.82	104.36	105.38	107.34
**3.5**	90.82	92.39	93.23	94.56	95.47	96.84	99.47	102.21	103.72	104.76	106.34	107.38	109.39
**3.75**	92.35	93.96	94.83	96.19	97.13	98.53	101.23	104.03	105.58	106.65	108.26	109.33	111.38
**4**	93.85	95.51	96.40	97.79	98.75	100.19	102.95	105.82	107.40	108.49	110.14	111.23	113.32
**4.25**	95.33	97.02	97.93	99.36	100.34	101.81	104.63	107.56	109.17	110.29	111.96	113.07	115.20
**4.5**	96.77	98.51	99.44	100.90	101.90	103.40	106.28	109.26	110.90	112.03	113.74	114.86	117.03
**4.75**	98.20	99.96	100.92	102.40	103.42	104.95	107.88	110.91	112.58	113.73	115.46	116.60	118.80
**5**	99.60	101.40	102.37	103.88	104.92	106.47	109.45	112.52	114.22	115.38	117.13	118.29	120.51
**5.25**	100.98	102.81	103.79	105.33	106.38	107.96	110.98	114.09	115.81	116.98	118.76	119.93	122.17
**5.5**	102.34	104.19	105.19	106.75	107.82	109.42	112.47	115.62	117.35	118.54	120.33	121.51	123.77
**5.75**	103.68	105.56	106.57	108.15	109.23	110.85	113.93	117.11	118.86	120.06	121.86	123.05	125.33
**6**	105.00	106.90	107.93	109.52	110.62	112.25	115.36	118.57	120.33	121.54	123.36	124.55	126.84
**6.25**	106.30	108.23	109.26	110.88	111.98	113.63	116.77	120.00	121.78	122.99	124.82	126.02	128.32
**6.5**	107.59	109.54	110.59	112.21	113.33	114.99	118.16	121.41	123.19	124.42	126.25	127.46	129.77
**6.75**	108.87	110.83	111.89	113.53	114.66	116.33	119.52	122.80	124.59	125.82	127.67	128.88	131.20
**7**	110.13	112.11	113.18	114.84	115.97	117.66	120.87	124.17	125.97	127.21	129.06	130.28	132.61
**7.25**	111.38	113.38	114.46	116.13	117.27	118.97	122.21	125.53	127.34	128.59	130.45	131.67	134.01
**7.5**	112.61	114.63	115.72	117.41	118.56	120.28	123.54	126.88	128.70	129.95	131.83	133.05	135.40
**7.75**	113.83	115.87	116.97	118.67	119.83	121.57	124.85	128.22	130.06	131.31	133.20	134.43	136.79
**8**	115.03	117.09	118.20	119.92	121.09	122.84	126.16	129.55	131.40	132.67	134.56	135.80	138.18
**8.25**	116.21	118.30	119.41	121.15	122.34	124.10	127.45	130.87	132.73	134.01	135.92	137.17	139.55
**8.5**	117.37	119.48	120.61	122.37	123.56	125.35	128.73	132.18	134.06	135.34	137.26	138.53	140.93
**8.75**	118.51	120.64	121.79	123.56	124.77	126.58	129.99	133.47	135.37	136.67	138.61	139.88	142.30
**9**	119.62	121.78	122.94	124.74	125.96	127.79	131.24	134.76	136.67	137.98	139.94	141.22	143.67
**9.25**	120.70	122.90	124.07	125.89	127.13	128.98	132.47	136.03	137.97	139.29	141.27	142.57	145.04
**9.5**	121.76	123.99	125.18	127.02	128.28	130.15	133.69	137.29	139.25	140.59	142.59	143.90	146.40
**9.75**	122.80	125.06	126.27	128.14	129.42	131.32	134.90	138.56	140.54	141.90	143.93	145.25	147.78
**10**	123.83	126.13	127.36	129.26	130.56	132.48	136.13	139.83	141.85	143.23	145.28	146.63	149.19
**10.25**	124.86	127.20	128.45	130.38	131.70	133.66	137.37	141.13	143.18	144.58	146.67	148.04	150.64
**10.5**	125.90	128.28	129.55	131.53	132.87	134.87	138.64	142.47	144.56	145.98	148.10	149.49	152.14
**10.75**	126.95	129.38	130.68	132.69	134.06	136.10	139.95	143.86	145.98	147.43	149.60	151.01	153.71
**11**	128.02	130.50	131.83	133.89	135.29	137.37	141.31	145.30	147.47	148.95	151.17	152.61	155.36
**11.25**	129.11	131.66	133.02	135.13	136.56	138.70	142.72	146.82	149.04	150.55	152.82	154.30	157.11
**11.5**	130.24	132.85	134.25	136.42	137.89	140.08	144.21	148.41	150.69	152.24	154.56	156.08	158.97

Age (years)	1st	3rd	5th	10th	15th	25th	50th	75th	85th	90th	95th	97th	99th
**11.75**	131.40	134.09	135.53	137.75	139.27	141.52	145.76	150.07	152.42	154.01	156.40	157.96	160.92
**12**	132.60	135.36	136.84	139.13	140.68	143.00	147.36	151.79	154.20	155.84	158.29	159.89	162.94
**12.25**	133.83	136.67	138.19	140.54	142.14	144.52	149.00	153.55	156.02	157.71	160.22	161.86	164.99
**12.5**	135.11	138.02	139.58	141.99	143.63	146.06	150.66	155.32	157.85	159.58	162.15	163.84	167.04
**12.75**	136.43	139.41	141.00	143.46	145.14	147.63	152.32	157.09	159.67	161.44	164.07	165.78	169.05
**13**	137.81	140.84	142.46	144.97	146.67	149.21	153.98	158.83	161.46	163.25	165.93	167.68	171.00
**13.25**	139.24	142.32	143.96	146.50	148.23	150.79	155.63	160.53	163.19	165.01	167.71	169.48	172.84
**13.5**	140.75	143.84	145.50	148.06	149.79	152.38	157.24	162.18	164.85	166.67	169.39	171.17	174.54
**13.75**	142.32	145.42	147.07	149.63	151.37	153.95	158.81	163.74	166.41	168.23	170.94	172.71	176.08
**14**	143.95	147.03	148.68	151.22	152.94	155.51	160.33	165.22	167.87	169.67	172.36	174.12	177.45
**14.25**	145.62	148.66	150.28	152.80	154.50	157.03	161.80	166.62	169.23	171.01	173.66	175.38	178.67
**14.5**	147.29	150.29	151.88	154.35	156.03	158.52	163.20	167.93	170.49	172.24	174.84	176.53	179.75
**14.75**	148.94	151.88	153.45	155.87	157.51	159.95	164.53	169.16	171.66	173.37	175.91	177.56	180.71
**15**	150.56	153.43	154.96	157.33	158.93	161.31	165.78	170.30	172.74	174.41	176.88	178.49	181.56
**15.25**	152.11	154.92	156.41	158.72	160.28	162.60	166.96	171.35	173.73	175.35	177.76	179.33	182.31
**15.5**	153.58	156.32	157.77	160.02	161.54	163.80	168.04	172.32	174.63	176.21	178.55	180.07	182.97
**15.75**	154.95	157.61	159.03	161.22	162.71	164.91	169.03	173.20	175.45	176.98	179.25	180.73	183.55
**16**	156.20	158.80	160.18	162.32	163.76	165.91	169.93	173.98	176.17	177.66	179.87	181.32	184.05
**16.25**	157.33	159.87	161.22	163.30	164.72	166.81	170.73	174.68	176.81	178.26	180.42	181.82	184.49
**16.5**	158.36	160.84	162.15	164.19	165.57	167.61	171.44	175.30	177.38	178.79	180.89	182.26	184.86
**16.75**	159.28	161.71	163.00	164.99	166.34	168.33	172.07	175.84	177.87	179.26	181.31	182.65	185.18
**17**	160.11	162.49	163.75	165.70	167.02	168.98	172.64	176.32	178.31	179.66	181.67	182.97	185.45
**17.25**	160.86	163.19	164.43	166.34	167.63	169.55	173.14	176.74	178.69	180.01	181.97	183.25	185.68
**17.5**	161.54	163.83	165.04	166.92	168.18	170.06	173.58	177.12	179.02	180.32	182.24	183.49	185.87
**17.75**	162.16	164.40	165.59	167.43	168.68	170.52	173.97	177.44	179.31	180.58	182.47	183.70	186.03
**18**	162.71	164.92	166.09	167.90	169.13	170.94	174.33	177.74	179.57	180.82	182.67	183.88	186.16

Note. Height is reported in centimeters.

Table S4. Weight-for-age model parameters for boys aged 2-10 years (0.25-year grid).

Age (years)	L (nu)	M (mu)	S (sigma)	K (tau)
**2**	-0.7879	12.9224	0.10616	1.9135
**2.25**	-0.7879	13.5583	0.10755	1.9135
**2.5**	-0.7879	14.1774	0.10894	1.9135
**2.75**	-0.7879	14.7727	0.11030	1.9135
**3**	-0.7879	15.3446	0.11162	1.9135
**3.25**	-0.7879	15.8812	0.11287	1.9135
**3.5**	-0.7879	16.3988	0.11405	1.9135
**3.75**	-0.7879	16.9123	0.11516	1.9135
**4**	-0.7879	17.4184	0.11615	1.9135
**4.25**	-0.7879	17.9293	0.11704	1.9135
**4.5**	-0.7879	18.4510	0.11786	1.9135
**4.75**	-0.7879	18.9695	0.11863	1.9135
**5**	-0.7879	19.4878	0.11940	1.9135
**5.25**	-0.7879	20.0094	0.12022	1.9135
**5.5**	-0.7879	20.5208	0.12109	1.9135
**5.75**	-0.7879	21.0298	0.12206	1.9135
**6**	-0.7879	21.5424	0.12316	1.9135
**6.25**	-0.7879	22.0701	0.12441	1.9135
**6.5**	-0.7879	22.6064	0.12583	1.9135
**6.75**	-0.7879	23.1617	0.12744	1.9135
**7**	-0.7879	23.7466	0.12930	1.9135
**7.25**	-0.7879	24.3496	0.13137	1.9135
**7.5**	-0.7879	24.9770	0.13365	1.9135
**7.75**	-0.7879	25.6357	0.13617	1.9135
**8**	-0.7879	26.3114	0.13887	1.9135
**8.25**	-0.7879	27.0109	0.14177	1.9135
**8.5**	-0.7879	27.7421	0.14490	1.9135
**8.75**	-0.7879	28.4869	0.14819	1.9135
**9**	-0.7879	29.2485	0.15167	1.9135
**9.25**	-0.7879	30.0307	0.15538	1.9135
**9.5**	-0.7879	30.8124	0.15923	1.9135
**9.75**	-0.7879	31.5980	0.16327	1.9135
**10**	-0.7879	32.3838	0.16748	1.9135

Note. L = nu (skewness/Box-Cox power); M = mu (median/location); S = sigma (coefficient of variation/scale); K = tau (kurtosis/tail shape). L (nu) and K (tau) were estimated as constants across age.

Table S5. Model-based weight-for-age centiles for boys aged 2-10 years (0.25-year grid).

Age (years)	1st	3rd	5th	10th	15th	25th	50th	75th	85th	90th	95th	97th	99th
**2**	10.29	10.73	10.97	11.36	11.64	12.06	12.92	13.90	14.48	14.91	15.60	16.08	17.06
**2.25**	10.77	11.23	11.49	11.90	12.19	12.65	13.56	14.59	15.22	15.68	16.41	16.92	17.98
**2.5**	11.23	11.72	11.99	12.43	12.73	13.21	14.18	15.28	15.94	16.43	17.20	17.75	18.87
**2.75**	11.67	12.18	12.47	12.93	13.25	13.76	14.77	15.93	16.64	17.15	17.97	18.55	19.74
**3**	12.09	12.62	12.92	13.41	13.75	14.28	15.34	16.56	17.31	17.85	18.72	19.33	20.59
**3.25**	12.48	13.04	13.35	13.86	14.21	14.76	15.88	17.16	17.94	18.50	19.42	20.06	21.39
**3.5**	12.86	13.44	13.76	14.29	14.66	15.23	16.40	17.73	18.55	19.14	20.10	20.77	22.16
**3.75**	13.23	13.84	14.17	14.72	15.10	15.70	16.91	18.30	19.15	19.77	20.77	21.47	22.93
**4**	13.60	14.23	14.58	15.14	15.54	16.16	17.42	18.86	19.75	20.39	21.43	22.16	23.69
**4.25**	13.98	14.62	14.98	15.57	15.98	16.62	17.93	19.43	20.34	21.02	22.10	22.86	24.45
**4.5**	14.36	15.03	15.40	16.01	16.43	17.10	18.45	20.01	20.96	21.65	22.78	23.57	25.22
**4.75**	14.74	15.43	15.82	16.44	16.88	17.57	18.97	20.58	21.56	22.29	23.45	24.28	25.99
**5**	15.12	15.83	16.23	16.88	17.33	18.04	19.49	21.15	22.17	22.92	24.13	24.98	26.76
**5.25**	15.50	16.24	16.65	17.31	17.78	18.52	20.01	21.73	22.79	23.56	24.82	25.70	27.55
**5.5**	15.87	16.63	17.05	17.74	18.22	18.98	20.52	22.30	23.39	24.20	25.49	26.41	28.33
**5.75**	16.24	17.01	17.45	18.16	18.66	19.44	21.03	22.87	24.00	24.83	26.17	27.13	29.12
**6**	16.60	17.40	17.85	18.58	19.10	19.90	21.55	23.45	24.62	25.48	26.88	27.87	29.93
**6.25**	16.96	17.79	18.25	19.00	19.54	20.37	22.07	24.04	25.25	26.15	27.60	28.62	30.78
**6.5**	17.33	18.18	18.66	19.43	19.99	20.85	22.61	24.65	25.91	26.84	28.35	29.41	31.66
**6.75**	17.70	18.58	19.07	19.88	20.45	21.34	23.16	25.28	26.60	27.56	29.13	30.25	32.60
**7**	18.08	18.99	19.50	20.33	20.93	21.85	23.75	25.96	27.33	28.34	29.98	31.15	33.62
**7.25**	18.47	19.41	19.94	20.80	21.42	22.38	24.35	26.66	28.09	29.15	30.87	32.10	34.69
**7.5**	18.86	19.84	20.39	21.28	21.92	22.92	24.98	27.39	28.89	30.00	31.81	33.10	35.84
**7.75**	19.27	20.28	20.85	21.78	22.45	23.49	25.64	28.16	29.73	30.90	32.81	34.18	37.08
**8**	19.68	20.73	21.32	22.29	22.98	24.07	26.31	28.96	30.61	31.84	33.86	35.30	38.38
**8.25**	20.09	21.18	21.80	22.81	23.53	24.66	27.01	29.79	31.53	32.83	34.96	36.49	39.76
**8.5**	20.51	21.65	22.29	23.34	24.10	25.28	27.74	30.67	32.50	33.88	36.14	37.76	41.25
**8.75**	20.94	22.12	22.79	23.88	24.67	25.91	28.49	31.56	33.51	34.96	37.36	39.09	42.81
**9**	21.36	22.59	23.28	24.43	25.25	26.54	29.25	32.49	34.54	36.08	38.63	40.47	44.46
**9.25**	21.78	23.06	23.79	24.98	25.84	27.19	30.03	33.45	35.62	37.26	39.96	41.94	46.20
**9.5**	22.19	23.52	24.28	25.52	26.42	27.83	30.81	34.41	36.71	38.45	41.33	43.44	48.02
**9.75**	22.59	23.97	24.76	26.05	26.99	28.47	31.60	35.40	37.83	39.67	42.74	44.99	49.91
**10**	22.98	24.41	25.23	26.58	27.56	29.11	32.38	36.39	38.96	40.92	44.20	46.60	51.89

Note. Weight is reported in kilograms.

Table S6. BMI-for-age model parameters for boys aged 2-18 years (0.25-year grid).

Age (years)	L (nu)	M (mu)	S (sigma)	K (tau)
**2**	-2.8583	16.88	0.07239	2.0687
**2.25**	-2.8535	16.80	0.07302	2.0290
**2.5**	-2.8395	16.73	0.07362	1.9989
**2.75**	-2.8190	16.67	0.07422	1.9761
**3**	-2.7932	16.61	0.07485	1.9587
**3.25**	-2.7642	16.56	0.07552	1.9459
**3.5**	-2.7325	16.50	0.07625	1.9366
**3.75**	-2.6984	16.45	0.07708	1.9302
**4**	-2.6632	16.40	0.07798	1.9261
**4.25**	-2.6269	16.34	0.07897	1.9241
**4.5**	-2.5895	16.29	0.08009	1.9237
**4.75**	-2.5519	16.24	0.08132	1.9247
**5**	-2.5140	16.20	0.08268	1.9270
**5.25**	-2.4754	16.16	0.08421	1.9303
**5.5**	-2.4372	16.13	0.08587	1.9345
**5.75**	-2.3990	16.11	0.08770	1.9396
**6**	-2.3609	16.10	0.08968	1.9453
**6.25**	-2.3225	16.11	0.09182	1.9518
**6.5**	-2.2848	16.12	0.09407	1.9587
**6.75**	-2.2472	16.15	0.09642	1.9661
**7**	-2.2096	16.19	0.09889	1.9741
**7.25**	-2.1727	16.24	0.10143	1.9823
**7.5**	-2.1360	16.30	0.10404	1.9909
**7.75**	-2.0994	16.37	0.10676	2.0000
**8**	-2.0634	16.45	0.10951	2.0093
**8.25**	-2.0278	16.53	0.11229	2.0188
**8.5**	-1.9922	16.63	0.11510	2.0287
**8.75**	-1.9574	16.73	0.11788	2.0387
**9**	-1.9229	16.84	0.12061	2.0489
**9.25**	-1.8884	16.95	0.12332	2.0594
**9.5**	-1.8547	17.08	0.12591	2.0699
**9.75**	-1.8213	17.20	0.12842	2.0807
**10**	-1.7883	17.33	0.13081	2.0915
**10.25**	-1.7553	17.47	0.13309	2.1027

Age (years)	L (nu)	M (mu)	S (sigma)	K (tau)
**10.5**	-1.7230	17.62	0.13521	2.1138
**10.75**	-1.6911	17.76	0.13717	2.1251
**11**	-1.6592	17.92	0.13898	2.1366
**11.25**	-1.6280	18.08	0.14061	2.1481
**11.5**	-1.5971	18.24	0.14207	2.1596
**11.75**	-1.5663	18.41	0.14336	2.1714
**12**	-1.5361	18.58	0.14447	2.1832
**12.25**	-1.5063	18.76	0.14539	2.1950
**12.5**	-1.4765	18.94	0.14614	2.2070
**12.75**	-1.4473	19.12	0.14671	2.2190
**13**	-1.4184	19.30	0.14709	2.2310
**13.25**	-1.3896	19.48	0.14730	2.2432
**13.5**	-1.3613	19.67	0.14733	2.2553
**13.75**	-1.3334	19.85	0.14718	2.2675
**14**	-1.3058	20.03	0.14687	2.2798
**14.25**	-1.2782	20.21	0.14639	2.2922
**14.5**	-1.2512	20.39	0.14578	2.3046
**14.75**	-1.2244	20.57	0.14504	2.3169
**15**	-1.1977	20.74	0.14420	2.3295
**15.25**	-1.1715	20.91	0.14327	2.3420
**15.5**	-1.1456	21.08	0.14227	2.3545
**15.75**	-1.1197	21.25	0.14119	2.3671
**16**	-1.0943	21.41	0.14008	2.3797
**16.25**	-1.0692	21.57	0.13894	2.3923
**16.5**	-1.0441	21.74	0.13775	2.4050
**16.75**	-1.0195	21.89	0.13656	2.4177
**17**	-0.9952	22.05	0.13536	2.4303
**17.25**	-0.9708	22.20	0.13415	2.4432
**17.5**	-0.9470	22.35	0.13295	2.4559
**17.75**	-0.9233	22.50	0.13177	2.4687
**18**	-0.9000	22.65	0.13060	2.4814

Note. L = nu (skewness/Box-Cox power); M = mu (median/location); S = sigma (coefficient of variation/scale); K = tau (kurtosis/tail shape). All four parameters were age-varying.

Table S7. Model-based BMI-for-age centiles for boys aged 2-18 years (0.25-year grid).

Age (years)	1st	3rd	5th	10th	15th	25th	50th	75th	85th	90th	95th	97th	99th
**2**	14.72	15.05	15.23	15.54	15.76	16.11	16.88	17.80	18.37	18.80	19.52	20.04	21.18
**2.25**	14.63	14.96	15.15	15.46	15.68	16.04	16.80	17.72	18.30	18.73	19.46	19.99	21.17
**2.5**	14.55	14.89	15.08	15.39	15.62	15.97	16.73	17.65	18.23	18.67	19.41	19.95	21.16
**2.75**	14.48	14.82	15.01	15.33	15.55	15.91	16.67	17.59	18.18	18.62	19.36	19.91	21.14
**3**	14.41	14.75	14.95	15.26	15.49	15.85	16.61	17.54	18.13	18.57	19.32	19.88	21.13
**3.25**	14.34	14.68	14.88	15.20	15.43	15.79	16.56	17.48	18.08	18.53	19.28	19.85	21.11
**3.5**	14.27	14.62	14.82	15.14	15.37	15.74	16.50	17.43	18.03	18.48	19.25	19.82	21.09
**3.75**	14.20	14.55	14.75	15.08	15.31	15.68	16.45	17.39	17.99	18.44	19.21	19.79	21.08
**4**	14.13	14.48	14.68	15.01	15.25	15.62	16.40	17.34	17.95	18.41	19.19	19.77	21.08
**4.25**	14.05	14.41	14.61	14.95	15.18	15.56	16.34	17.30	17.91	18.38	19.16	19.75	21.07
**4.5**	13.98	14.34	14.55	14.88	15.12	15.50	16.29	17.26	17.87	18.35	19.14	19.74	21.08
**4.75**	13.90	14.27	14.48	14.81	15.06	15.44	16.24	17.22	17.85	18.32	19.13	19.74	21.10
**5**	13.83	14.20	14.41	14.75	14.99	15.38	16.20	17.19	17.82	18.31	19.13	19.75	21.14
**5.25**	13.76	14.13	14.34	14.69	14.94	15.33	16.16	17.16	17.81	18.31	19.15	19.77	21.19
**5.5**	13.69	14.07	14.28	14.63	14.89	15.29	16.13	17.15	17.82	18.32	19.18	19.82	21.27
**5.75**	13.63	14.01	14.23	14.59	14.84	15.25	16.11	17.16	17.84	18.35	19.23	19.89	21.38
**6**	13.58	13.97	14.19	14.55	14.81	15.22	16.10	17.18	17.87	18.40	19.31	19.98	21.52
**6.25**	13.53	13.93	14.15	14.52	14.78	15.21	16.11	17.21	17.93	18.47	19.40	20.10	21.69
**6.5**	13.49	13.89	14.12	14.50	14.77	15.20	16.12	17.26	17.99	18.56	19.52	20.24	21.88
**6.75**	13.46	13.87	14.10	14.48	14.76	15.20	16.15	17.32	18.08	18.66	19.65	20.40	22.11
**7**	13.43	13.85	14.09	14.48	14.76	15.22	16.19	17.39	18.18	18.78	19.81	20.58	22.35
**7.25**	13.42	13.84	14.08	14.48	14.77	15.24	16.24	17.48	18.29	18.91	19.98	20.78	22.63
**7.5**	13.41	13.84	14.09	14.49	14.79	15.27	16.30	17.58	18.42	19.07	20.17	21.00	22.93
**7.75**	13.40	13.84	14.09	14.51	14.82	15.31	16.37	17.70	18.56	19.23	20.38	21.24	23.25
**8**	13.40	13.85	14.11	14.54	14.85	15.35	16.45	17.82	18.72	19.41	20.60	21.50	23.59
**8.25**	13.41	13.87	14.13	14.57	14.89	15.40	16.53	17.95	18.88	19.60	20.84	21.77	23.95
**8.5**	13.42	13.89	14.16	14.61	14.94	15.47	16.63	18.10	19.06	19.81	21.09	22.06	24.33
**8.75**	13.44	13.92	14.20	14.65	14.99	15.53	16.73	18.25	19.25	20.02	21.35	22.36	24.72
**9**	13.47	13.95	14.24	14.70	15.05	15.60	16.84	18.41	19.44	20.24	21.61	22.66	25.12
**9.25**	13.50	13.99	14.28	14.76	15.11	15.68	16.95	18.58	19.64	20.47	21.89	22.97	25.52
**9.5**	13.53	14.04	14.33	14.82	15.18	15.77	17.08	18.75	19.85	20.70	22.17	23.29	25.92
**9.75**	13.58	14.09	14.39	14.89	15.26	15.86	17.20	18.93	20.06	20.94	22.45	23.61	26.32
**10**	13.62	14.15	14.45	14.96	15.34	15.95	17.34	19.11	20.28	21.18	22.74	23.92	26.71
**10.25**	13.68	14.21	14.52	15.04	15.43	16.06	17.47	19.30	20.50	21.42	23.02	24.24	27.10
**10.5**	13.74	14.28	14.60	15.13	15.52	16.16	17.62	19.49	20.72	21.67	23.31	24.55	27.47
**10.75**	13.80	14.36	14.68	15.22	15.62	16.28	17.77	19.69	20.94	21.92	23.59	24.86	27.83
**11**	13.88	14.44	14.77	15.32	15.73	16.40	17.92	19.89	21.17	22.16	23.87	25.16	28.18
**11.25**	13.96	14.53	14.86	15.42	15.84	16.52	18.08	20.09	21.40	22.41	24.14	25.46	28.51
**11.5**	14.04	14.62	14.96	15.53	15.95	16.65	18.24	20.29	21.63	22.66	24.42	25.75	28.82

Age (years)	1st	3rd	5th	10th	15th	25th	50th	75th	85th	90th	95th	97th	99th
**11.75**	14.14	14.72	15.07	15.65	16.08	16.79	18.41	20.50	21.86	22.90	24.68	26.03	29.12
**12**	14.23	14.83	15.18	15.77	16.21	16.93	18.58	20.71	22.09	23.15	24.95	26.30	29.39
**12.25**	14.34	14.95	15.30	15.90	16.34	17.08	18.76	20.92	22.32	23.39	25.20	26.56	29.65
**12.5**	14.45	15.06	15.42	16.03	16.48	17.23	18.94	21.14	22.55	23.63	25.45	26.81	29.90
**12.75**	14.57	15.19	15.55	16.17	16.62	17.38	19.12	21.35	22.78	23.86	25.69	27.05	30.12
**13**	14.69	15.32	15.68	16.31	16.77	17.54	19.30	21.56	23.00	24.09	25.92	27.28	30.32
**13.25**	14.81	15.45	15.82	16.45	16.92	17.70	19.48	21.77	23.22	24.31	26.14	27.49	30.50
**13.5**	14.94	15.59	15.96	16.60	17.07	17.86	19.67	21.97	23.43	24.53	26.35	27.69	30.66
**13.75**	15.07	15.72	16.10	16.75	17.23	18.02	19.85	22.18	23.64	24.73	26.55	27.88	30.80
**14**	15.21	15.87	16.25	16.90	17.38	18.19	20.03	22.37	23.84	24.93	26.74	28.05	30.92
**14.25**	15.35	16.01	16.40	17.05	17.54	18.35	20.21	22.57	24.03	25.12	26.91	28.21	31.02
**14.5**	15.49	16.16	16.55	17.21	17.70	18.52	20.39	22.75	24.22	25.30	27.07	28.35	31.11
**14.75**	15.63	16.31	16.70	17.36	17.86	18.68	20.57	22.94	24.40	25.47	27.23	28.48	31.18
**15**	15.78	16.45	16.85	17.52	18.02	18.84	20.74	23.12	24.57	25.64	27.37	28.61	31.24
**15.25**	15.92	16.60	17.00	17.67	18.18	19.01	20.91	23.29	24.74	25.80	27.51	28.72	31.29
**15.5**	16.07	16.75	17.15	17.83	18.33	19.17	21.08	23.46	24.90	25.95	27.64	28.83	31.34
**15.75**	16.21	16.90	17.30	17.98	18.49	19.33	21.25	23.63	25.06	26.10	27.76	28.93	31.38
**16**	16.36	17.05	17.45	18.14	18.65	19.49	21.41	23.79	25.21	26.24	27.87	29.02	31.41
**16.25**	16.51	17.20	17.61	18.29	18.80	19.64	21.58	23.95	25.36	26.37	27.99	29.11	31.44
**16.5**	16.65	17.35	17.76	18.44	18.95	19.80	21.74	24.10	25.50	26.51	28.10	29.20	31.47
**16.75**	16.80	17.50	17.91	18.59	19.11	19.95	21.89	24.25	25.64	26.64	28.20	29.28	31.50
**17**	16.94	17.65	18.05	18.74	19.26	20.11	22.05	24.40	25.78	26.76	28.30	29.37	31.53
**17.25**	17.09	17.79	18.20	18.89	19.41	20.26	22.20	24.55	25.92	26.89	28.40	29.45	31.56
**17.5**	17.23	17.94	18.35	19.04	19.56	20.41	22.35	24.69	26.05	27.01	28.50	29.53	31.59
**17.75**	17.38	18.08	18.49	19.19	19.70	20.56	22.50	24.84	26.18	27.13	28.60	29.60	31.62
**18**	17.52	18.22	18.64	19.33	19.85	20.70	22.65	24.98	26.31	27.25	28.70	29.68	31.66

Note. BMI is reported in kg/m².

Table S8. Height-for-age model parameters for girls aged 2-18 years (0.25-year grid).

Age (years)	L (nu)	M (mu)	S (sigma)	K (tau)
**2**	0.9217	85.96	0.03968	1.8194
**2.25**	0.7230	87.96	0.04391	1.8489
**2.5**	0.5566	91.30	0.04517	1.8751
**2.75**	0.4173	94.12	0.04473	1.8984
**3**	0.2994	95.81	0.04489	1.9193
**3.25**	0.2023	96.94	0.04592	1.9377
**3.5**	0.1217	98.41	0.04674	1.9540
**3.75**	0.0549	100.16	0.04671	1.9685
**4**	0.0015	101.90	0.04624	1.9813
**4.25**	-0.0410	103.63	0.04580	1.9926
**4.5**	-0.0740	105.34	0.04563	2.0026
**4.75**	-0.0979	106.95	0.04569	2.0112
**5**	-0.1141	108.43	0.04590	2.0187
**5.25**	-0.1234	109.80	0.04619	2.0252
**5.5**	-0.1263	111.08	0.04651	2.0307
**5.75**	-0.1236	112.32	0.04681	2.0353
**6**	-0.1157	113.62	0.04705	2.0391
**6.25**	-0.1031	115.09	0.04722	2.0422
**6.5**	-0.0864	116.70	0.04732	2.0446
**6.75**	-0.0662	118.32	0.04739	2.0464
**7**	-0.0425	119.87	0.04748	2.0476
**7.25**	-0.0165	121.23	0.04764	2.0484
**7.5**	0.0117	122.49	0.04788	2.0487
**7.75**	0.0423	123.75	0.04824	2.0487
**8**	0.0742	125.03	0.04867	2.0483
**8.25**	0.1075	126.38	0.04916	2.0476
**8.5**	0.1424	127.80	0.04969	2.0467
**8.75**	0.1779	129.23	0.05022	2.0455
**9**	0.2143	130.67	0.05075	2.0441
**9.25**	0.2518	132.13	0.05127	2.0425
**9.5**	0.2896	133.60	0.05171	2.0408
**9.75**	0.3278	135.09	0.05205	2.0388
**10**	0.3663	136.61	0.05225	2.0368
**10.25**	0.4056	138.20	0.05229	2.0346

Age (years)	L (nu)	M (mu)	S (sigma)	K (tau)
**10.5**	0.4447	139.81	0.05214	2.0323
**10.75**	0.4840	141.43	0.05178	2.0299
**11**	0.5238	143.07	0.05122	2.0274
**11.25**	0.5631	144.67	0.05050	2.0249
**11.5**	0.6025	146.25	0.04966	2.0223
**11.75**	0.6423	147.79	0.04872	2.0196
**12**	0.6816	149.25	0.04773	2.0169
**12.25**	0.7207	150.61	0.04673	2.0141
**12.5**	0.7601	151.88	0.04571	2.0113
**12.75**	0.7989	153.01	0.04472	2.0084
**13**	0.8376	154.00	0.04376	2.0055
**13.25**	0.8764	154.86	0.04283	2.0026
**13.5**	0.9146	155.59	0.04196	1.9997
**13.75**	0.9525	156.22	0.04114	1.9967
**14**	0.9902	156.75	0.04036	1.9937
**14.25**	1.0280	157.20	0.03963	1.9907
**14.5**	1.0651	157.57	0.03896	1.9878
**14.75**	1.1019	157.89	0.03834	1.9848
**15**	1.1389	158.15	0.03777	1.9817
**15.25**	1.1751	158.36	0.03725	1.9788
**15.5**	1.2110	158.52	0.03679	1.9758
**15.75**	1.2470	158.65	0.03637	1.9727
**16**	1.2822	158.75	0.03600	1.9697
**16.25**	1.3172	158.81	0.03567	1.9668
**16.5**	1.3522	158.85	0.03537	1.9638
**16.75**	1.3864	158.87	0.03510	1.9608
**17**	1.4204	158.88	0.03487	1.9578
**17.25**	1.4544	158.87	0.03466	1.9548
**17.5**	1.4877	158.86	0.03447	1.9519
**17.75**	1.5206	158.84	0.03430	1.9490
**18**	1.5532	158.83	0.03415	1.9461

Note. L = nu (skewness/Box-Cox power); M = mu (median/location); S = sigma (coefficient of variation/scale); K = tau (kurtosis/tail shape). All four parameters were age-varying.

Table S9. Model-based height-for-age centiles for girls aged 2-18 years (0.25-year grid).

**Age (years)**	**1st**	**3rd**	**5th**	**10th**	**15th**	**25th**	**50th**	**75th**	**85th**	**90th**	**95th**	**97th**	**99th**
**2**	77.87	79.50	80.35	81.64	82.49	83.73	85.95	88.18	89.43	90.29	91.59	92.44	94.09
**2.25**	78.93	80.71	81.65	83.08	84.03	85.42	87.95	90.50	91.92	92.90	94.38	95.36	97.24
**2.5**	81.79	83.65	84.62	86.12	87.13	88.60	91.31	94.05	95.57	96.63	98.21	99.26	101.28
**2.75**	84.52	86.37	87.35	88.85	89.87	91.36	94.13	96.95	98.51	99.59	101.22	102.29	104.36
**3**	86.07	87.93	88.91	90.43	91.46	92.98	95.81	98.70	100.31	101.41	103.08	104.18	106.30
**3.25**	86.95	88.83	89.83	91.39	92.44	94.01	96.94	99.95	101.62	102.77	104.50	105.64	107.84
**3.5**	88.16	90.07	91.10	92.69	93.77	95.38	98.42	101.54	103.27	104.46	106.25	107.43	109.71
**3.75**	89.77	91.70	92.73	94.33	95.43	97.06	100.15	103.34	105.10	106.31	108.14	109.34	111.66
**4**	91.49	93.41	94.44	96.04	97.14	98.78	101.90	105.12	106.90	108.12	109.96	111.17	113.50
**4.25**	93.17	95.09	96.12	97.73	98.84	100.48	103.63	106.88	108.67	109.90	111.75	112.97	115.32
**4.5**	94.77	96.70	97.74	99.36	100.47	102.14	105.33	108.63	110.44	111.69	113.56	114.80	117.17
**4.75**	96.23	98.18	99.23	100.88	102.01	103.70	106.95	110.30	112.15	113.42	115.32	116.58	118.99
**5**	97.54	99.51	100.58	102.25	103.40	105.12	108.43	111.86	113.74	115.03	116.97	118.25	120.70
**5.25**	98.71	100.72	101.80	103.50	104.67	106.42	109.80	113.29	115.21	116.53	118.50	119.80	122.30
**5.5**	99.80	101.84	102.94	104.67	105.85	107.64	111.08	114.64	116.60	117.94	119.95	121.27	123.81
**5.75**	100.85	102.92	104.04	105.79	107.00	108.82	112.32	115.95	117.94	119.30	121.35	122.70	125.28
**6**	101.97	104.07	105.21	106.99	108.22	110.06	113.63	117.32	119.35	120.73	122.81	124.18	126.80
**6.25**	103.24	105.37	106.53	108.34	109.59	111.46	115.09	118.85	120.90	122.31	124.42	125.81	128.46
**6.5**	104.65	106.82	108.00	109.84	111.10	113.01	116.70	120.52	122.60	124.03	126.17	127.58	130.27
**6.75**	106.08	108.29	109.48	111.36	112.65	114.59	118.33	122.21	124.32	125.77	127.94	129.37	132.09
**7**	107.42	109.67	110.88	112.78	114.09	116.06	119.86	123.79	125.94	127.41	129.61	131.05	133.81
**7.25**	108.59	110.87	112.11	114.04	115.37	117.37	121.23	125.22	127.40	128.88	131.11	132.57	135.37
**7.5**	109.65	111.97	113.22	115.19	116.54	118.57	122.50	126.55	128.76	130.27	132.52	134.00	136.84
**7.75**	110.65	113.02	114.29	116.30	117.68	119.75	123.74	127.86	130.11	131.64	133.94	135.44	138.31
**8**	111.66	114.08	115.39	117.44	118.84	120.96	125.03	129.23	131.51	133.08	135.41	136.94	139.86
**8.25**	112.72	115.20	116.53	118.63	120.06	122.23	126.39	130.67	133.00	134.59	136.97	138.53	141.51
**8.5**	113.81	116.35	117.71	119.86	121.33	123.54	127.79	132.17	134.55	136.17	138.60	140.19	143.23
**8.75**	114.91	117.51	118.92	121.11	122.62	124.88	129.23	133.70	136.13	137.79	140.26	141.89	144.98
**9**	116.02	118.69	120.12	122.37	123.92	126.23	130.68	135.24	137.72	139.41	141.94	143.59	146.75
**9.25**	117.13	119.87	121.34	123.64	125.22	127.59	132.13	136.79	139.32	141.04	143.62	145.30	148.52
**9.5**	118.27	121.07	122.58	124.93	126.55	128.96	133.60	138.34	140.92	142.68	145.30	147.01	150.28
**9.75**	119.46	122.32	123.86	126.26	127.91	130.38	135.09	139.92	142.54	144.32	146.98	148.73	152.04
**10**	120.72	123.64	125.20	127.65	129.33	131.83	136.62	141.52	144.17	145.98	148.68	150.44	153.80
**10.25**	122.06	125.03	126.62	129.11	130.81	133.35	138.20	143.15	145.83	147.66	150.38	152.16	155.55
**10.5**	123.48	126.49	128.11	130.63	132.35	134.92	139.81	144.80	147.50	149.34	152.08	153.87	157.27
**10.75**	124.98	128.03	129.66	132.20	133.93	136.52	141.44	146.45	149.15	151.00	153.74	155.54	158.94
**11**	126.55	129.62	131.26	133.81	135.55	138.14	143.06	148.07	150.78	152.62	155.36	157.14	160.54

Age (years)	1st	3rd	5th	10th	15th	25th	50th	75th	85th	90th	95th	97th	99th
**11.25**	128.16	131.24	132.88	135.43	137.17	139.76	144.67	149.66	152.35	154.18	156.90	158.68	162.05
**11.5**	129.79	132.87	134.51	137.06	138.79	141.37	146.25	151.20	153.87	155.69	158.39	160.15	163.49
**11.75**	131.42	134.48	136.12	138.66	140.38	142.94	147.79	152.69	155.33	157.13	159.80	161.53	164.83
**12**	133.00	136.06	137.68	140.20	141.91	144.46	149.25	154.09	156.70	158.47	161.11	162.83	166.08
**12.25**	134.53	137.56	139.17	141.67	143.37	145.88	150.62	155.40	157.97	159.72	162.31	164.00	167.21
**12.5**	135.96	138.97	140.57	143.04	144.72	147.20	151.88	156.59	159.12	160.84	163.40	165.06	168.21
**12.75**	137.28	140.26	141.84	144.29	145.95	148.40	153.01	157.65	160.14	161.83	164.35	165.98	169.07
**13**	138.47	141.42	142.99	145.41	147.04	149.47	154.00	158.56	161.02	162.68	165.15	166.76	169.80
**13.25**	139.53	142.45	144.00	146.39	148.00	150.39	154.86	159.34	161.76	163.39	165.82	167.40	170.38
**13.5**	140.47	143.36	144.89	147.25	148.84	151.19	155.59	160.00	162.37	163.98	166.36	167.91	170.84
**13.75**	141.30	144.15	145.67	147.99	149.57	151.89	156.22	160.55	162.88	164.46	166.81	168.33	171.21
**14**	142.03	144.85	146.35	148.65	150.20	152.49	156.75	161.01	163.30	164.86	167.16	168.66	171.48
**14.25**	142.67	145.46	146.94	149.21	150.74	153.00	157.20	161.39	163.65	165.17	167.44	168.91	171.69
**14.5**	143.23	145.99	147.45	149.70	151.21	153.44	157.57	161.70	163.92	165.42	167.65	169.10	171.83
**14.75**	143.71	146.45	147.90	150.12	151.61	153.81	157.89	161.95	164.14	165.62	167.81	169.24	171.93
**15**	144.13	146.84	148.28	150.47	151.95	154.12	158.15	162.16	164.31	165.77	167.93	169.34	171.99
**15.25**	144.49	147.18	148.60	150.77	152.23	154.38	158.36	162.31	164.44	165.88	168.01	169.40	172.01
**15.5**	144.79	147.46	148.86	151.02	152.47	154.59	158.52	162.43	164.53	165.95	168.06	169.43	172.01
**15.75**	145.04	147.69	149.08	151.22	152.66	154.76	158.65	162.51	164.59	165.99	168.08	169.43	171.98
**16**	145.24	147.87	149.26	151.38	152.81	154.90	158.75	162.57	164.62	166.01	168.07	169.41	171.94
**16.25**	145.40	148.02	149.40	151.51	152.92	155.00	158.81	162.60	164.63	166.01	168.05	169.37	171.87
**16.5**	145.52	148.13	149.51	151.60	153.01	155.07	158.85	162.60	164.62	165.98	168.01	169.32	171.80
**16.75**	145.62	148.22	149.59	151.67	153.07	155.12	158.87	162.59	164.59	165.94	167.95	169.25	171.71
**17**	145.69	148.28	149.64	151.72	153.11	155.15	158.88	162.57	164.55	165.90	167.89	169.18	171.62
**17.25**	145.74	148.33	149.69	151.76	153.14	155.16	158.87	162.54	164.51	165.84	167.82	169.10	171.52
**17.5**	145.78	148.36	149.71	151.78	153.16	155.17	158.86	162.50	164.46	165.79	167.75	169.03	171.43
**17.75**	145.81	148.39	149.74	151.80	153.17	155.18	158.84	162.47	164.41	165.73	167.68	168.95	171.34
**18**	145.83	148.41	149.76	151.81	153.18	155.18	158.83	162.43	164.37	165.68	167.62	168.89	171.27

Note. Height is reported in centimeters.

Table S10. Weight-for-age model parameters for girls aged 2-10 years (0.25-year grid).

Age (years)	L (nu)	M (mu)	S (sigma)	K (tau)
**2**	-0.4288	12.3983	0.10673	2.0000
**2.25**	-0.4288	12.8753	0.11176	2.0000
**2.5**	-0.4288	13.9160	0.11251	2.0000
**2.75**	-0.4288	14.7412	0.11255	2.0000
**3**	-0.4288	15.1991	0.11354	2.0000
**3.25**	-0.4288	15.4507	0.11532	2.0000
**3.5**	-0.4288	15.9142	0.11655	2.0000
**3.75**	-0.4288	16.4258	0.11715	2.0000
**4**	-0.4288	17.0035	0.11784	2.0000
**4.25**	-0.4288	17.5532	0.11884	2.0000
**4.5**	-0.4288	17.9965	0.11988	2.0000
**4.75**	-0.4288	18.5377	0.12062	2.0000
**5**	-0.4288	19.0514	0.12142	2.0000
**5.25**	-0.4288	19.4662	0.12268	2.0000
**5.5**	-0.4288	19.9602	0.12452	2.0000
**5.75**	-0.4288	20.4809	0.12670	2.0000
**6**	-0.4288	20.8579	0.12881	2.0000
**6.25**	-0.4288	21.2620	0.13058	2.0000
**6.5**	-0.4288	21.9056	0.13180	2.0000
**6.75**	-0.4288	22.6539	0.13270	2.0000
**7**	-0.4288	23.2967	0.13359	2.0000
**7.25**	-0.4288	23.8294	0.13467	2.0000
**7.5**	-0.4288	24.3676	0.13608	2.0000
**7.75**	-0.4288	24.9819	0.13788	2.0000
**8**	-0.4288	25.6818	0.14000	2.0000
**8.25**	-0.4288	26.4721	0.14239	2.0000
**8.5**	-0.4288	27.2863	0.14500	2.0000
**8.75**	-0.4288	28.0249	0.14764	2.0000
**9**	-0.4288	28.6937	0.15028	2.0000
**9.25**	-0.4288	29.3971	0.15294	2.0000
**9.5**	-0.4288	30.2088	0.15555	2.0000
**9.75**	-0.4288	31.1496	0.15814	2.0000
**10**	-0.4288	32.1661	0.16070	2.0000

Note. L = nu (skewness/Box-Cox power); M = mu (median/location); S = sigma (coefficient of variation/scale); K = tau (kurtosis/tail shape). L (nu) was estimated as a constant across age and K (tau) was fixed at 2.0000.

Table S11. Model-based weight-for-age centiles for girls aged 2-10 years (0.25-year grid).

Age (years)	1st	3rd	5th	10th	15th	25th	50th	75th	85th	90th	95th	97th	99th
**2**	9.79	10.23	10.47	10.86	11.13	11.55	12.40	13.34	13.89	14.28	14.88	15.29	16.12
**2.25**	10.06	10.53	10.79	11.20	11.50	11.95	12.88	13.90	14.50	14.93	15.59	16.05	16.96
**2.5**	10.86	11.36	11.65	12.10	12.42	12.91	13.92	15.03	15.68	16.15	16.88	17.37	18.37
**2.75**	11.50	12.04	12.34	12.82	13.16	13.68	14.74	15.92	16.62	17.11	17.88	18.40	19.46
**3**	11.83	12.39	12.70	13.20	13.55	14.10	15.20	16.43	17.15	17.66	18.47	19.01	20.12
**3.25**	11.99	12.56	12.87	13.39	13.75	14.31	15.45	16.72	17.47	18.00	18.83	19.40	20.55
**3.5**	12.31	12.91	13.24	13.77	14.15	14.73	15.91	17.24	18.02	18.57	19.44	20.03	21.23
**3.75**	12.69	13.31	13.65	14.20	14.59	15.20	16.43	17.80	18.61	19.18	20.09	20.70	21.95
**4**	13.12	13.76	14.11	14.69	15.10	15.73	17.00	18.44	19.28	19.88	20.82	21.46	22.76
**4.25**	13.52	14.18	14.55	15.15	15.57	16.22	17.55	19.04	19.92	20.55	21.53	22.20	23.56
**4.5**	13.83	14.51	14.89	15.51	15.94	16.62	18.00	19.54	20.45	21.10	22.11	22.81	24.22
**4.75**	14.22	14.93	15.32	15.96	16.41	17.11	18.54	20.14	21.08	21.75	22.81	23.53	25.00
**5**	14.59	15.32	15.73	16.39	16.85	17.58	19.05	20.71	21.68	22.38	23.47	24.23	25.74
**5.25**	14.87	15.62	16.04	16.72	17.20	17.95	19.47	21.18	22.19	22.91	24.04	24.82	26.39
**5.5**	15.19	15.97	16.40	17.11	17.60	18.38	19.96	21.74	22.79	23.55	24.73	25.55	27.20
**5.75**	15.52	16.32	16.77	17.51	18.02	18.83	20.48	22.34	23.44	24.23	25.48	26.33	28.07
**6**	15.74	16.57	17.03	17.79	18.32	19.16	20.86	22.79	23.94	24.76	26.05	26.94	28.75
**6.25**	15.98	16.83	17.31	18.09	18.64	19.50	21.26	23.26	24.44	25.29	26.64	27.56	29.44
**6.5**	16.42	17.31	17.80	18.61	19.18	20.08	21.91	23.98	25.22	26.10	27.50	28.47	30.43
**6.75**	16.95	17.87	18.39	19.22	19.82	20.75	22.65	24.82	26.10	27.03	28.49	29.50	31.55
**7**	17.40	18.35	18.88	19.75	20.36	21.33	23.30	25.54	26.87	27.83	29.34	30.39	32.52
**7.25**	17.76	18.74	19.28	20.17	20.81	21.80	23.83	26.14	27.52	28.51	30.07	31.16	33.36
**7.5**	18.11	19.11	19.68	20.60	21.25	22.27	24.37	26.76	28.18	29.21	30.83	31.96	34.24
**7.75**	18.50	19.54	20.12	21.07	21.75	22.80	24.98	27.47	28.95	30.02	31.71	32.89	35.28
**8**	18.93	20.01	20.62	21.61	22.31	23.41	25.68	28.28	29.83	30.95	32.73	33.96	36.47
**8.25**	19.42	20.54	21.18	22.21	22.94	24.09	26.47	29.20	30.83	32.01	33.88	35.18	37.84
**8.5**	19.91	21.08	21.74	22.82	23.59	24.79	27.29	30.15	31.87	33.12	35.09	36.47	39.28
**8.75**	20.34	21.56	22.24	23.36	24.16	25.42	28.02	31.03	32.83	34.14	36.22	37.67	40.63
**9**	20.72	21.97	22.69	23.85	24.68	25.98	28.69	31.83	33.71	35.08	37.26	38.78	41.91
**9.25**	21.11	22.41	23.15	24.35	25.22	26.57	29.40	32.67	34.64	36.08	38.36	39.96	43.25
**9.5**	21.58	22.93	23.70	24.95	25.85	27.26	30.21	33.63	35.70	37.21	39.61	41.29	44.76
**9.75**	22.14	23.54	24.34	25.65	26.59	28.06	31.15	34.74	36.92	38.51	41.04	42.82	46.49
**10**	22.74	24.21	25.04	26.41	27.39	28.93	32.17	35.94	38.23	39.91	42.58	44.46	48.34

Note. Weight is reported in kilograms.

Table S12. BMI-for-age model parameters for girls aged 2-18 years (0.25-year grid).

Age (years)	L (nu)	M (mu)	S (sigma)	K (tau)
**2**	-2.4070	16.81	0.06640	2.0000
**2.25**	-2.3656	16.70	0.06703	2.0000
**2.5**	-2.3267	16.62	0.06768	2.0000
**2.75**	-2.2898	16.55	0.06837	2.0000
**3**	-2.2544	16.49	0.06910	2.0000
**3.25**	-2.2210	16.44	0.06988	2.0000
**3.5**	-2.1889	16.38	0.07073	2.0000
**3.75**	-2.1577	16.33	0.07164	2.0000
**4**	-2.1280	16.28	0.07262	2.0000
**4.25**	-2.0992	16.23	0.07366	2.0000
**4.5**	-2.0711	16.18	0.07479	2.0000
**4.75**	-2.0442	16.15	0.07598	2.0000
**5**	-2.0180	16.11	0.07724	2.0000
**5.25**	-1.9922	16.09	0.07859	2.0000
**5.5**	-1.9674	16.08	0.08001	2.0000
**5.75**	-1.9432	16.07	0.08151	2.0000
**6**	-1.9195	16.07	0.08310	2.0000
**6.25**	-1.8961	16.08	0.08480	2.0000
**6.5**	-1.8735	16.10	0.08658	2.0000
**6.75**	-1.8514	16.12	0.08846	2.0000
**7**	-1.8295	16.16	0.09048	2.0000
**7.25**	-1.8082	16.20	0.09259	2.0000
**7.5**	-1.7874	16.24	0.09482	2.0000
**7.75**	-1.7666	16.30	0.09721	2.0000
**8**	-1.7465	16.36	0.09970	2.0000
**8.25**	-1.7267	16.43	0.10229	2.0000
**8.5**	-1.7071	16.51	0.10499	2.0000
**8.75**	-1.6879	16.59	0.10769	2.0000
**9**	-1.6691	16.69	0.11039	2.0000
**9.25**	-1.6503	16.79	0.11308	2.0000
**9.5**	-1.6320	16.91	0.11566	2.0000
**9.75**	-1.6140	17.04	0.11813	2.0000
**10**	-1.5963	17.18	0.12045	2.0000
**10.25**	-1.5785	17.33	0.12261	2.0000

Age (years)	L (nu)	M (mu)	S (sigma)	K (tau)
**10.5**	-1.5612	17.49	0.12453	2.0000
**10.75**	-1.5442	17.66	0.12620	2.0000
**11**	-1.5271	17.85	0.12761	2.0000
**11.25**	-1.5105	18.04	0.12873	2.0000
**11.5**	-1.4941	18.25	0.12961	2.0000
**11.75**	-1.4776	18.46	0.13027	2.0000
**12**	-1.4616	18.67	0.13069	2.0000
**12.25**	-1.4457	18.89	0.13093	2.0000
**12.5**	-1.4298	19.11	0.13098	2.0000
**12.75**	-1.4143	19.33	0.13087	2.0000
**13**	-1.3990	19.54	0.13061	2.0000
**13.25**	-1.3836	19.76	0.13021	2.0000
**13.5**	-1.3686	19.96	0.12971	2.0000
**13.75**	-1.3537	20.17	0.12910	2.0000
**14**	-1.3389	20.36	0.12841	2.0000
**14.25**	-1.3242	20.55	0.12763	2.0000
**14.5**	-1.3097	20.74	0.12680	2.0000
**14.75**	-1.2954	20.91	0.12592	2.0000
**15**	-1.2811	21.07	0.12500	2.0000
**15.25**	-1.2670	21.23	0.12406	2.0000
**15.5**	-1.2531	21.38	0.12310	2.0000
**15.75**	-1.2391	21.53	0.12213	2.0000
**16**	-1.2254	21.66	0.12117	2.0000
**16.25**	-1.2119	21.79	0.12022	2.0000
**16.5**	-1.1983	21.92	0.11928	2.0000
**16.75**	-1.1849	22.03	0.11837	2.0000
**17**	-1.1717	22.14	0.11749	2.0000
**17.25**	-1.1584	22.25	0.11663	2.0000
**17.5**	-1.1454	22.35	0.11583	2.0000
**17.75**	-1.1325	22.44	0.11508	2.0000
**18**	-1.1197	22.53	0.11437	2.0000

Note. L = nu (skewness/Box-Cox power); M = mu (median/location); S = sigma (coefficient of variation/scale); K = tau (kurtosis/tail shape). K (tau) was fixed at 2.0000.

Table S13. Model-based BMI-for-age centiles for girls aged 2-18 years (0.25-year grid).

Age (years)	1st	3rd	5th	10th	15th	25th	50th	75th	85th	90th	95th	97th	99th
**2**	14.74	15.08	15.26	15.56	15.78	16.11	16.81	17.63	18.13	18.49	19.09	19.51	20.40
**2.25**	14.63	14.96	15.14	15.44	15.66	16.00	16.70	17.52	18.02	18.38	18.98	19.40	20.29
**2.5**	14.53	14.86	15.05	15.35	15.57	15.91	16.62	17.44	17.94	18.31	18.90	19.32	20.22
**2.75**	14.45	14.78	14.97	15.28	15.50	15.84	16.55	17.37	17.88	18.25	18.84	19.27	20.17
**3**	14.38	14.71	14.90	15.21	15.43	15.77	16.49	17.32	17.83	18.20	18.80	19.23	20.13
**3.25**	14.31	14.65	14.84	15.15	15.37	15.71	16.44	17.27	17.79	18.16	18.77	19.20	20.11
**3.5**	14.23	14.58	14.77	15.08	15.31	15.66	16.38	17.23	17.74	18.12	18.74	19.17	20.09
**3.75**	14.16	14.51	14.70	15.02	15.24	15.59	16.33	17.18	17.70	18.09	18.71	19.15	20.07
**4**	14.09	14.44	14.63	14.95	15.18	15.54	16.28	17.14	17.67	18.06	18.68	19.13	20.07
**4.25**	14.02	14.37	14.57	14.89	15.12	15.48	16.23	17.10	17.64	18.03	18.66	19.11	20.07
**4.5**	13.95	14.30	14.50	14.83	15.06	15.43	16.18	17.07	17.61	18.01	18.65	19.11	20.08
**4.75**	13.88	14.24	14.45	14.77	15.01	15.38	16.15	17.04	17.59	18.00	18.65	19.12	20.10
**5**	13.82	14.19	14.39	14.72	14.96	15.34	16.11	17.03	17.58	18.00	18.66	19.14	20.14
**5.25**	13.77	14.14	14.34	14.68	14.92	15.30	16.09	17.02	17.59	18.01	18.68	19.17	20.20
**5.5**	13.72	14.09	14.30	14.64	14.89	15.27	16.08	17.02	17.60	18.03	18.72	19.22	20.27
**5.75**	13.67	14.05	14.26	14.61	14.86	15.25	16.07	17.03	17.62	18.06	18.77	19.28	20.36
**6**	13.63	14.02	14.23	14.59	14.84	15.24	16.07	17.05	17.66	18.10	18.83	19.35	20.46
**6.25**	13.60	13.99	14.21	14.57	14.82	15.23	16.08	17.08	17.70	18.16	18.91	19.44	20.59
**6.5**	13.57	13.97	14.19	14.55	14.82	15.23	16.10	17.12	17.76	18.23	19.00	19.55	20.73
**6.75**	13.54	13.95	14.17	14.55	14.81	15.24	16.12	17.18	17.83	18.31	19.10	19.67	20.89
**7**	13.52	13.93	14.16	14.54	14.82	15.25	16.16	17.24	17.91	18.40	19.22	19.81	21.07
**7.25**	13.50	13.92	14.16	14.55	14.82	15.27	16.20	17.31	18.00	18.51	19.35	19.96	21.28
**7.5**	13.49	13.91	14.16	14.55	14.84	15.29	16.24	17.39	18.10	18.63	19.50	20.14	21.50
**7.75**	13.47	13.91	14.16	14.56	14.85	15.32	16.30	17.47	18.21	18.76	19.67	20.32	21.75
**8**	13.46	13.91	14.16	14.58	14.88	15.35	16.36	17.57	18.33	18.90	19.85	20.53	22.02
**8.25**	13.46	13.91	14.17	14.60	14.90	15.39	16.43	17.68	18.47	19.06	20.04	20.76	22.32
**8.5**	13.46	13.92	14.19	14.62	14.94	15.44	16.51	17.80	18.62	19.23	20.25	21.00	22.64
**8.75**	13.46	13.94	14.21	14.66	14.98	15.49	16.59	17.93	18.78	19.42	20.48	21.26	22.98
**9**	13.48	13.96	14.24	14.70	15.03	15.56	16.69	18.07	18.95	19.61	20.72	21.53	23.34
**9.25**	13.50	14.00	14.28	14.75	15.09	15.63	16.79	18.22	19.13	19.82	20.97	21.82	23.71
**9.5**	13.53	14.04	14.33	14.81	15.16	15.71	16.91	18.38	19.32	20.04	21.24	22.12	24.10
**9.75**	13.57	14.09	14.39	14.88	15.24	15.81	17.04	18.55	19.53	20.27	21.51	22.43	24.50
**10**	13.62	14.16	14.46	14.96	15.33	15.91	17.18	18.74	19.74	20.51	21.80	22.75	24.90
**10.25**	13.69	14.23	14.54	15.06	15.43	16.03	17.33	18.93	19.97	20.76	22.09	23.08	25.31
**10.5**	13.77	14.32	14.64	15.17	15.55	16.16	17.49	19.14	20.20	21.01	22.38	23.41	25.72
**10.75**	13.86	14.43	14.75	15.29	15.68	16.30	17.66	19.35	20.44	21.27	22.68	23.74	26.12
**11**	13.97	14.55	14.88	15.42	15.82	16.46	17.85	19.57	20.69	21.54	22.99	24.07	26.51

Age (years)	1st	3rd	5th	10th	15th	25th	50th	75th	85th	90th	95th	97th	99th
**11.25**	14.09	14.68	15.02	15.57	15.98	16.63	18.04	19.80	20.94	21.81	23.29	24.39	26.88
**11.5**	14.23	14.82	15.17	15.73	16.15	16.81	18.25	20.04	21.20	22.09	23.59	24.71	27.24
**11.75**	14.37	14.98	15.32	15.90	16.32	17.00	18.46	20.28	21.46	22.36	23.88	25.02	27.59
**12**	14.52	15.14	15.49	16.08	16.50	17.19	18.67	20.52	21.72	22.63	24.17	25.32	27.92
**12.25**	14.68	15.30	15.66	16.26	16.69	17.38	18.89	20.76	21.97	22.89	24.45	25.61	28.22
**12.5**	14.84	15.47	15.84	16.44	16.88	17.58	19.11	21.00	22.22	23.15	24.72	25.89	28.51
**12.75**	15.00	15.65	16.02	16.63	17.07	17.78	19.33	21.24	22.47	23.40	24.98	26.15	28.78
**13**	15.17	15.82	16.20	16.81	17.26	17.98	19.54	21.47	22.71	23.65	25.23	26.41	29.03
**13.25**	15.34	16.00	16.38	17.00	17.46	18.18	19.76	21.70	22.94	23.88	25.47	26.64	29.26
**13.5**	15.51	16.17	16.56	17.19	17.65	18.38	19.96	21.91	23.16	24.11	25.69	26.86	29.46
**13.75**	15.68	16.35	16.73	17.37	17.83	18.57	20.17	22.13	23.38	24.32	25.91	27.07	29.65
**14**	15.84	16.52	16.91	17.55	18.02	18.76	20.36	22.33	23.58	24.52	26.10	27.26	29.82
**14.25**	16.00	16.69	17.08	17.73	18.20	18.95	20.55	22.52	23.77	24.72	26.29	27.44	29.97
**14.5**	16.16	16.85	17.24	17.90	18.37	19.12	20.74	22.70	23.95	24.89	26.46	27.60	30.11
**14.75**	16.31	17.01	17.40	18.06	18.53	19.29	20.91	22.88	24.12	25.06	26.61	27.74	30.22
**15**	16.46	17.16	17.56	18.22	18.69	19.45	21.07	23.04	24.28	25.21	26.75	27.88	30.32
**15.25**	16.60	17.31	17.71	18.37	18.85	19.61	21.23	23.20	24.43	25.36	26.89	28.00	30.41
**15.5**	16.74	17.45	17.85	18.52	19.00	19.76	21.38	23.34	24.57	25.49	27.01	28.11	30.49
**15.75**	16.88	17.59	17.99	18.66	19.14	19.90	21.53	23.48	24.70	25.62	27.12	28.21	30.55
**16**	17.00	17.72	18.12	18.79	19.27	20.04	21.66	23.61	24.83	25.73	27.22	28.30	30.61
**16.25**	17.13	17.85	18.25	18.92	19.40	20.17	21.79	23.73	24.94	25.84	27.32	28.38	30.66
**16.5**	17.25	17.97	18.37	19.05	19.53	20.30	21.92	23.85	25.05	25.95	27.41	28.46	30.71
**16.75**	17.36	18.08	18.49	19.16	19.65	20.41	22.03	23.96	25.16	26.04	27.49	28.53	30.75
**17**	17.47	18.19	18.60	19.28	19.76	20.53	22.14	24.06	25.25	26.13	27.57	28.60	30.78
**17.25**	17.57	18.30	18.71	19.38	19.87	20.63	22.25	24.16	25.34	26.22	27.64	28.66	30.81
**17.5**	17.67	18.40	18.81	19.48	19.97	20.74	22.35	24.25	25.43	26.29	27.70	28.71	30.84
**17.75**	17.76	18.49	18.90	19.58	20.06	20.83	22.44	24.34	25.51	26.37	27.77	28.76	30.87
**18**	17.85	18.58	18.99	19.67	20.15	20.92	22.53	24.42	25.58	26.44	27.83	28.81	30.89

Note. *BMI is reported in* kg/m^2.

Table S14. Age-group Q-statistic diagnostics for the selected height-for-age model in boys aged 2-18 years.

Q-statistics (goodness of fit), BCPE

Age interval	Z1 (mean)	Z2 (variance)	Z3 (skewness)	Z4 (kurtosis)	D'Agostino K²	*N*
2.0000 to 2.3942	-0.562	0.629	0.547	-0.376	0.440	2,332
2.3942 to 3.5660	1.327	-0.797	0.673	0.072	0.458	2,324
3.5660 to 4.9349	-1.127	0.507	-1.054	-0.427	1.293	2,328
4.9349 to 6.3175	0.412	-0.602	1.138	-1.188	2.706	2,327
6.3175 to 7.3333	0.251	0.293	-1.400	0.290	2.044	2,331
7.3333 to 8.8172	-0.940	0.679	-0.255	-0.776	0.667	2,326
8.8172 to 10.1122	0.950	-0.132	0.585	-0.488	0.580	2,330
10.1122 to 11.3223	-0.723	-0.579	-0.861	0.331	0.850	2,326
11.3223 to 12.3408	0.577	-0.135	-0.094	-0.637	0.415	2,335
12.3408 to 13.3785	-0.182	0.175	0.501	-0.794	0.881	2,323
13.3785 to 14.7227	-0.154	0.445	0.770	0.361	0.724	2,325
14.7227 to 18.0000	0.180	-0.487	-0.435	-1.529	2.527	2,326
**TOTAL Q stats**	**6.310**	**3.035**	**7.310**	**6.277**	**13.587**	**27,933**
**df for Q stats**	**2.412**	**7.293**	**9.435**	**12.000**	**21.435**	**0**
***p*-val for Q stats**	**0.062**	**0.899**	**0.646**	**0.901**	**0.900**	**0**

Table S15. Age-group Q-statistic diagnostics for the selected weight-for-age model in boys aged 2-10 years.

Q-statistics (goodness of fit), BCPE

Age interval	Z1 (mean)	Z2 (variance)	Z3 (skewness)	Z4 (kurtosis)	D'Agostino K^2	*N*
730.5 to 737.5	-0.504	1.085	0.639	-0.905	1.228	436
737.5 to 746.5	1.297	-0.809	-1.236	-0.494	1.772	392
746.5 to 761.5	0.685	0.084	-0.119	-0.410	0.182	378
761.5 to 788.5	-1.418	-1.149	-0.632	-1.345	2.209	405
788.5 to 830.5	-0.060	0.634	-0.470	0.259	0.288	406
830.5 to 889.5	0.019	1.033	0.187	-0.621	0.420	395
889.5 to 969.5	-0.298	-0.590	-1.876	0.505	3.775	407
969.5 to 1073.5	0.381	0.082	-0.698	-0.397	0.645	397
1073.5 to 1106.5	-0.207	-0.701	0.596	0.796	0.988	404
1106.5 to 1161.5	0.004	-1.015	-0.890	-0.998	1.788	401
1161.5 to 1252.5	0.268	0.228	-0.311	-0.637	0.502	400
1252.5 to 1341.5	0.233	0.028	0.016	0.239	0.057	401
1341.5 to 1439.5	-1.783	0.607	-1.606	1.410	4.568	405
1439.5 to 1495.5	0.267	-0.842	-0.988	0.972	1.920	398
1495.5 to 1579.5	-0.126	1.559	-0.206	-0.555	0.350	405
1579.5 to 1674.5	1.104	0.653	-0.340	1.486	2.323	400
1674.5 to 1768.5	-1.002	-0.242	-1.258	-0.696	2.066	402
1768.5 to 1850.5	1.424	0.028	-1.125	0.320	1.367	402
1850.5 to 1966.5	0.113	-0.566	1.270	-0.021	1.613	404
1966.5 to 2086.5	0.079	0.924	2.093	1.437	6.449	397
2086.5 to 2196.5	-1.375	0.670	-0.902	-0.300	0.904	413
2196.5 to 2245.5	2.353	-1.118	1.226	2.300	6.791	394
2245.5 to 2295.5	-2.660	-0.828	0.561	0.088	0.323	405
2295.5 to 2346.5	0.502	-1.383	1.362	1.056	2.970	404
2346.5 to 2398.5	0.777	0.166	-0.136	-0.857	0.753	400
2398.5 to 2455.5	-0.761	-0.060	-1.399	-0.439	2.149	399
2455.5 to 2517.5	0.126	-0.595	0.851	1.295	2.401	398
2517.5 to 2587.5	0.236	0.899	-0.247	-1.837	3.434	402
2587.5 to 2680.5	0.412	0.233	0.723	-0.347	0.644	401
2680.5 to 2785.5	-0.819	0.159	-0.485	-0.142	0.256	404
2785.5 to 2875.5	0.459	-0.280	-0.160	0.361	0.156	402
2875.5 to 2967.5	0.852	-0.339	0.814	0.136	0.682	404
2967.5 to 3069.5	-0.517	0.055	0.514	0.993	1.250	406
3069.5 to 3155.5	-0.982	1.758	1.060	-1.062	2.251	393

**Age interval**	**Z1 (mean)**	**Z2 (variance)**	**Z3 (skewness)**	**Z4 (kurtosis)**	**D'Agostino K^2**	***N***
3155.5 to 3241.5	-0.569	-0.411	0.260	-2.023	4.158	405
3241.5 to 3318.5	0.265	0.166	1.173	0.972	2.320	402
3318.5 to 3407.5	1.300	-1.821	1.267	-0.409	1.772	401
3407.5 to 3493.5	0.450	-0.116	-0.374	0.314	0.238	405
3493.5 to 3577.5	-1.344	-2.111	-2.538	0.016	6.443	396
3577.5 to 3652.5	0.710	2.706	1.157	-1.418	3.348	402
**TOTAL Q stats**	**35.976**	**35.888**	**41.459**	**36.295**	**77.754**	**16,071**
**df for Q stats**	**33.347**	**37.441**	**39.000**	**39.000**	**78.000**	**0**
***p*-val for Q stats**	**0.346**	**0.542**	**0.364**	**0.594**	**0.487**	**0**

Table S16. Age-group Q-statistic diagnostics for the selected BMI-for-age model in boys aged 2-18 years.

Q-statistics (goodness of fit), BCPE

Age interval	Z1 (mean)	Z2 (variance)	Z3 (skewness)	Z4 (kurtosis)	D'Agostino K²	*N*
2.0000 to 2.3942	0.019	-0.257	0.082	-0.738	0.552	2,332
2.3942 to 3.5660	0.474	0.134	-0.895	-1.380	2.704	2,324
3.5660 to 4.9349	-0.572	-0.022	1.071	0.582	1.487	2,328
4.9349 to 6.3175	-0.244	-0.081	1.418	1.027	3.066	2,327
6.3175 to 7.3333	0.394	-0.018	-1.105	-0.486	1.456	2,331
7.3333 to 8.8172	0.041	0.205	1.226	-2.178	6.247	2,326
8.8172 to 10.1122	0.047	0.124	0.004	0.449	0.202	2,330
10.1122 to 11.3223	-0.916	0.315	0.100	0.903	0.825	2,326
11.3223 to 12.3408	-0.988	-1.300	-0.514	-1.545	2.652	2,335
12.3408 to 13.3785	1.540	0.307	-1.172	-1.312	3.095	2,323
13.3785 to 14.7227	-0.786	1.929	0.619	-1.044	1.473	2,325
14.7227 to 18.0000	0.764	-1.449	0.791	0.140	0.646	2,326
**TOTAL Q stats**	**6.159**	**7.853**	**9.346**	**15.056**	**24.403**	**27,933**
**df for Q stats**	**3.105**	**7.961**	**8.864**	**8.862**	**17.726**	**0**
***p*-val for Q stats**	**0.112**	**0.444**	**0.393**	**0.085**	**0.133**	**0**

Table S17. Age-group Q-statistic diagnostics for the selected height-for-age model in girls aged 2-18 years.

Q-statistics (goodness of fit), BCPE

Age Interval	Z_1 (Mean)	Z_2 (Variance)	Z_3 (Skewness)	Z_4 (Kurtosis)	D'Agostino K^2	*N*
2.00 to 2.22	0.542	0.035	-0.484	-0.733	0.772	1,763
2.22 to 3.04	-0.930	-1.070	0.179	0.297	0.120	1,780
3.04 to 4.09	0.452	1.151	-0.257	0.208	0.109	1,771
4.09 to 5.27	-0.027	-0.128	0.913	-1.032	1.900	1,773
5.27 to 6.28	0.490	0.414	-0.422	-0.746	0.735	1,766
6.28 to 6.92	-0.037	-1.494	0.955	0.799	1.552	1,777
6.92 to 7.83	-0.041	0.827	-0.379	-0.199	0.183	1,769
7.82 to 8.64	-0.166	0.354	-0.522	0.399	0.431	1,767
8.64 to 9.35	-0.440	-0.200	0.349	-0.496	0.367	1,772
9.35 to 10.03	0.005	-0.419	-0.853	-0.394	0.883	1,773
10.02 to 10.63	-0.335	0.226	-0.439	-1.378	2.092	1,779
10.63 to 11.22	1.245	0.385	0.054	-0.903	0.819	1,765
11.22 to 11.84	-1.001	1.022	1.373	-0.812	2.544	1,772
11.84 to 12.50	0.133	-1.101	0.534	0.647	0.705	1,774
12.49 to 13.34	0.122	0.243	-0.542	-0.670	0.742	1,772
13.33 to 14.78	0.045	-0.608	-0.004	0.235	0.055	1,775
14.78 to 16.48	-0.336	-0.431	-0.568	-0.820	0.994	1,769
16.47 to 17.37	0.132	0.552	0.131	-0.848	0.737	1,772
17.37 to 18.00	0.132	0.300	-0.281	-1.160	1.425	1,775
**TOTAL Q stats**	**4.675**	**9.382**	**6.600**	**10.566**	**17.166**	**33,664**
**df for Q stats**	**2.065**	**13.127**	**15.639**	**15.977**	**31.617**	**0**
***p*-val for Q stats**	**0.102**	**0.752**	**0.976**	**0.834**	**0.983**	**0**

Table S18. Age-group Q-statistic diagnostics for the selected weight-for-age model in girls aged 2-10 years.

Q-statistics (goodness of fit), BCPE

Age interval	Z1 (mean)	Z2 (variance)	Z3 (skewness)	Z4 (kurtosis)	D'Agostino K^2	*N*
730.5 to 736.5	-0.619	-0.879	0.099	0.729	0.541	411
736.5 to 748.5	-0.031	-0.693	-0.335	0.487	0.350	427
748.5 to 767.5	0.560	0.226	0.419	-0.693	0.656	390
767.5 to 799.5	0.717	1.209	-1.966	1.272	5.483	393
799.5 to 852.5	0.409	2.222	-1.406	-0.297	2.065	407
852.5 to 924.5	-1.680	-2.212	-0.486	-0.837	0.936	403
924.5 to 1023.5	1.200	-0.417	1.099	-0.475	1.434	401
1023.5 to 1098.5	-1.659	0.156	-0.188	-0.589	0.382	412
1098.5 to 1131.5	1.304	-1.632	0.220	0.544	0.345	399
1131.5 to 1211.5	0.623	1.403	-0.524	1.706	3.184	407
1211.5 to 1330.5	-0.345	0.503	0.731	-0.534	0.819	401
1330.5 to 1454.5	-0.860	0.504	-0.212	1.709	2.965	408
1454.5 to 1513.5	0.377	-2.740	1.130	-0.004	1.277	404
1513.5 to 1607.5	0.285	1.437	-1.886	-0.587	3.901	405
1607.5 to 1710.5	-0.440	1.088	-0.752	-0.109	0.578	405
1710.5 to 1819.5	0.444	0.028	-0.399	0.621	0.544	408
1819.5 to 1932.5	-0.366	-0.658	-0.020	-1.147	1.316	398
1932.5 to 2062.5	-0.346	-1.446	-0.356	-0.771	0.721	406
2062.5 to 2179.5	0.879	0.148	-0.063	0.168	0.032	405
2179.5 to 2260.5	-0.019	0.450	0.848	0.211	0.763	408
2260.5 to 2338.5	-0.288	1.476	-0.576	0.808	0.984	399
2338.5 to 2422.5	-1.158	0.219	1.476	0.565	2.498	409
2422.5 to 2511.5	1.412	0.373	-0.859	0.770	1.331	403
2511.5 to 2591.5	-0.338	-1.308	0.198	1.827	3.376	403
2591.5 to 2672.5	0.209	-0.137	-0.081	-1.556	2.427	406
2672.5 to 2755.5	-0.561	0.222	0.840	0.930	1.570	404
2755.5 to 2835.5	1.188	-0.882	-1.078	-0.050	1.164	405
2835.5 to 2913.5	-0.768	0.037	-0.813	-0.818	1.329	407
2913.5 to 2983.5	0.024	-0.646	-0.060	-1.092	1.197	404
2983.5 to 3050.5	-0.780	0.964	-0.205	-0.193	0.079	404
3050.5 to 3123.5	-0.738	-0.388	1.297	-1.468	3.837	402
3123.5 to 3181.5	1.587	0.063	-0.994	0.566	1.308	407
3181.5 to 3247.5	0.467	1.301	-1.113	0.577	1.571	404
3247.5 to 3309.5	0.391	-1.002	0.116	0.910	0.841	409

Age interval	Z1 (mean)	Z2 (variance)	Z3 (skewness)	Z4 (kurtosis)	D'Agostino K²	*N*
3309.5 to 3367.5	0.776	-0.171	0.397	1.238	1.692	404
3367.5 to 3422.5	-1.912	-0.593	1.149	-1.221	2.812	411
3422.5 to 3478.5	-1.848	0.453	1.873	2.183	8.274	394
3478.5 to 3537.5	1.351	-0.919	2.562	-0.402	6.726	412
3537.5 to 3595.5	0.496	1.870	0.986	1.474	3.145	398
3595.5 to 3652.5	0.142	-0.872	-1.713	0.966	3.866	402
**TOTAL Q stats**	**32.675**	**46.245**	**40.176**	**38.145**	**78.320**	**16,185**
**df for Q stats**	**22.000**	**35.500**	**39.000**	**40.000**	**79.000**	**0**
***p*-val for Q stats**	**0.067**	**0.107**	**0.418**	**0.554**	**0.500**	**0**

Table S19. Age-group Q-statistic diagnostics for the selected BMI-for-age model in girls aged 2-18 years.

Q-statistics (goodness of fit), BCPE

Age Interval	Z_1 (Mean)	Z_2 (Variance)	Z_3 (Skewness)	Z_4 (Kurtosis)	D'Agostino K^2	*N*
2.00 to 3.01	-0.05383984	0.05145944	-0.68129876	-0.37427114	0.6042469	3,375
3.01 to 5.00	0.12218802	0.21697584	-1.48787784	0.25753308	2.2801037	3,363
5.00 to 6.74	-0.45929220	-0.15973992	1.18901457	0.01371781	1.4139438	3,363
6.74 to 8.33	0.06035841	-0.10359299	-1.41066944	-0.40231841	2.1518484	3,369
8.33 to 9.69	0.63329304	-0.75991306	1.31294336	-0.39729932	1.8816670	3,364
9.69 to 10.87	-0.95726114	0.26144650	2.16871920	-0.18660232	4.7381634	3,366
10.87 to 12.03	0.55966168	0.36096220	0.85203848	-0.87589251	1.4931573	3,373
12.03 to 13.55	-0.46156311	0.56707353	-0.76587671	-0.91340701	1.4208795	3,363
13.55 to 16.59	1.43074517	-0.41554354	-0.22605726	-2.04662409	4.2397720	3,363
16.59 to 18.00	-0.87083124	-0.01032029	-1.16614816	-0.07850097	1.3660639	3,365
TOTAL Q stats	4.88147056	1.35644611	15.23239574	6.35745026	21.5898460	33,664
df for Q stats	2.03713683	6.11403438	7.99999931	10.00000000	17.9999993	0
p-val for Q stats	0.08999738	0.97136419	0.05478109	0.78439124	0.2507046	0

Table S20. Selected GAMLSS model specifications for the six sex-specific growth-reference models.

Model	Age domain	Measurements / children	Family / links	Age transformation / smoother	mu specification (EDF)	sigma specification (EDF)	nu specification (EDF)	tau specification (EDF)	Global deviance / GAIC(k=2)	Convergence / software
Boys HFA	2-18 years	27,933 / 14,594	BCPE; identity/log/identity/log	(age in days/365.25)^0.80; penalized B-spline, pb()	height ~ pb(age_tr, inter = 8) (EDF 9.59)	~pb(age_tr, inter = 8) (EDF 8.41)	~pb(age_tr, inter = 2) (EDF 2.57)	fixed at 2 (EDF 0.00)	170884.43 / 170925.57	TRUE (6 iterations); R 4.5.3; gamlss 5.5-0; gamlss.dist 6.1-1
Boys WFA	2-10 years	16,071 / 9,537	BCPE; identity/log/identity/log	age in days^0.60; penalized B-spline, pb()	weight ~ pb(age_power_060, inter = 6) (EDF 6.65)	~pb(age_power_060, inter = 3) (EDF 4.12)	~1 (EDF 1.00)	~1 (EDF 1.00)	75654.93 / 75680.47	TRUE (3 iterations); R 4.5.3; gamlss 5.5-0; gamlss.dist 6.1-1
Boys BMIFA	2-18 years	27,933 / 14,594	BCPE; identity/log/identity/log	age in days^0.15; penalized B-spline, pb()	BMI ~ pb(age_day_trans, inter = 9) (EDF 8.89)	~pb(age_day_trans, inter = 7) (EDF 7.08)	~pb(age_day_trans, inter = 1) (EDF 3.14)	~pb(age_day_trans, inter = 1) (EDF 3.14)	118073.96 / 118118.45	TRUE (5 iterations); R 4.5.3; gamlss 5.5-0; gamlss.dist 6.1-1
Girls HFA	2-18 years	33,664 / 18,576	BCPE; identity/log/identity/log	age in days^0.05; penalized B-spline, pb()	new_height ~ pb(Age2, inter = 18) (EDF 16.94)	~pb(Age2, inter = 12) (EDF 10.75)	~pb(Age2, inter = 2) (EDF 3.36)	~pb(Age2, inter = 2) (EDF 3.02)	213995.63 / 214063.76	TRUE (4 iterations); R 4.5.3; gamlss 5.5-0; gamlss.dist 6.1-1
Girls WFA	2-10 years	16,185 / 10,214	BCPE; identity/log/identity/log	age in days^0.05; penalized B-spline, pb()	weight ~ pb(age_power, df = 16, inter = 20) (EDF 18.00)	~pb(age_power, df = 6, inter = 20) (EDF 8.00)	~1 (EDF 1.00)	fixed at 2 (EDF 0.00)	77623.11 / 77677.11	TRUE (6 iterations); R 4.5.3; gamlss 5.5-0; gamlss.dist 6.1-1
Girls BMIFA	2-18 years	33,664 / 18,576	BCPE; identity/log/identity/log	age in days^0.45; penalized B-spline, pb()	new_bmi_sim ~ pb(age_days_pwr, inter = 6) (EDF 7.96)	~pb(age_days_pwr, inter = 6) (EDF 6.77)	~pb(age_days_pwr, inter = 2) (EDF 2.00)	fixed at 2 (EDF 0.00)	138132.42 / 138165.89	TRUE (7 iterations); R 4.5.3; gamlss 5.5-0; gamlss.dist 6.1-1

Note. EDF = effective degrees of freedom; GAIC(k=2) is the Akaike information criterion. Parameter links are ordered mu/sigma/nu/tau. Only the six selected models are reported; the model-selection procedure evaluated multiple candidate structures for each parameter by sex. The software versions shown are those used for final model validation in 2026; R denotes the R statistical environment (R Foundation for Statistical Computing, Vienna, Austria). The gamlss and gamlss.dist packages were developed by Mikis Stasinopoulos and Robert Rigby (University of Greenwich, London, UK).

Table S21. Diagnostic summary for the six selected growth-reference models.

Model	Age / *N*	Global Q *p*-values (Z1 / Z2 / Z3 / Z4)	Maximum local |Z|	Residual mean / SD / skewness / excess kurtosis	Worm diagnostic	Convergence	Diagnostic conclusion
Boys HFA	2-18 years / 27,933	0.062 / 0.899 / 0.646 / 0.901	1.529 (Z4; 14.7227 to 18.0000)	0.000 / 1.000 / -0.000 / -0.047	12 bands; full-*N* coverage=TRUE	TRUE (6 iterations)	Accepted: global and local diagnostics pass. Global Q tests pass; no local |Z| exceeds 1.96.
Boys WFA	2-10 years / 16,071	0.346 / 0.542 / 0.364 / 0.594	2.706 (Z2; 3577.5 to 3652.5)	-0.000 / 1.000 / -0.005 / -0.012	8 bands; full-*N* coverage=TRUE	TRUE (3 iterations)	Accepted: global diagnostics pass; isolated local signal noted. All global Q tests and final diagnostic criteria were met; isolated local deviations are reported in the detailed Q-statistic table.
Boys BMIFA	2-18 years / 27,933	0.112 / 0.444 / 0.393 / 0.085	2.178 (Z4; 7.3333 to 8.8172)	-0.000 / 1.000 / 0.007 / -0.048	12 bands; full-*N* coverage=TRUE	TRUE (5 iterations)	Accepted: all prespecified global Q-statistic, residual, convergence, and worm-plot criteria were met.
Girls HFA	2-18 years / 33,664	0.102 / 0.752 / 0.976 / 0.834	1.494 (Z2; 6.28 to 6.92 years)	-0.000 / 1.000 / -0.001 / -0.053	16 bands; full-*N* coverage=TRUE	TRUE (4 iterations)	Accepted: global and local diagnostics pass. Global Q tests pass; no local |Z| exceeds 2.
Girls WFA	2-10 years / 16,185	0.067 / 0.107 / 0.418 / 0.554	2.740 (Z2; 1454.5 to 1513.5)	0.000 / 1.000 / -0.005 / 0.047	8 bands; full-*N* coverage=TRUE	TRUE (6 iterations)	Accepted: global diagnostics pass; isolated local signal noted. All prespecified Q-statistic, residual, centile-geometry, and worm-plot criteria were met; isolated local deviations are reported in the detailed Q-statistic table.
Girls BMIFA	2-18 years / 33,664	0.090 / 0.971 / 0.055 / 0.784	2.169 (Z3; approximately 9.69-10.87 years)	0.000 / 1.000 / -0.001 / -0.045	16 bands; full-*N* coverage=TRUE	TRUE (7 iterations)	Accepted: global diagnostics pass; isolated local signal noted. Model accepted: all global Q, residual, and worm diagnostics pass. Isolated local Z3 and Z4 signals were observed at approximately 9.69-10.87 and 13.55-16.59 years without global Q-statistic failure.

Note. All six selected models met the global Q-statistic, residual, convergence, and worm-plot acceptance criteria. Detailed local Q statistics are provided for completeness.

Table S22. Empirical and model-based percentile comparisons by age group for the six selected growth-reference models.

Model	Age band	*N*	Prediction age (years)	Centile	Empirical	Model-based	Model - empirical	Absolute difference / unit
Boys HFA	[2.0, 2.5)	2,543	2.094	P3	82.38	82.29	-0.10	0.10 cm
Boys HFA	[2.0, 2.5)	2,543	2.094	P50	88.60	88.21	-0.40	0.40 cm
Boys HFA	[2.0, 2.5)	2,543	2.094	P97	95.56	94.91	-0.65	0.65 cm
Boys HFA	[2.5, 3.0)	830	2.757	P3	87.58	87.42	-0.16	0.16 cm
Boys HFA	[2.5, 3.0)	830	2.757	P50	93.88	93.91	0.02	0.02 cm
Boys HFA	[2.5, 3.0)	830	2.757	P97	101.76	101.20	-0.56	0.56 cm
Boys HFA	[3.0, 3.5)	1,173	3.146	P3	90.09	90.09	0.00	0.00 cm
Boys HFA	[3.0, 3.5)	1,173	3.146	P50	97.08	96.89	-0.18	0.18 cm
Boys HFA	[3.0, 3.5)	1,173	3.146	P97	105.60	104.52	-1.08	1.08 cm
Boys HFA	[3.5, 4.0)	787	3.759	P3	93.26	94.02	0.76	0.76 cm
Boys HFA	[3.5, 4.0)	787	3.759	P50	100.97	101.29	0.33	0.33 cm
Boys HFA	[3.5, 4.0)	787	3.759	P97	108.93	109.40	0.47	0.47 cm
Boys HFA	[4.0, 4.5)	970	4.189	P3	96.83	96.65	-0.18	0.18 cm
Boys HFA	[4.0, 4.5)	970	4.189	P50	104.51	104.23	-0.28	0.28 cm
Boys HFA	[4.0, 4.5)	970	4.189	P97	113.15	112.62	-0.52	0.52 cm
Boys HFA	[4.5, 5.0)	802	4.758	P3	99.40	100.01	0.61	0.61 cm
Boys HFA	[4.5, 5.0)	802	4.758	P50	107.88	107.93	0.05	0.05 cm
Boys HFA	[4.5, 5.0)	802	4.758	P97	117.22	116.66	-0.56	0.56 cm
Boys HFA	[5.0, 5.5)	656	5.216	P3	102.62	102.62	0.00	0.00 cm
Boys HFA	[5.0, 5.5)	656	5.216	P50	110.62	110.77	0.15	0.15 cm
Boys HFA	[5.0, 5.5)	656	5.216	P97	119.61	119.70	0.10	0.10 cm
Boys HFA	[5.5, 6.0)	649	5.752	P3	105.63	105.57	-0.06	0.06 cm
Boys HFA	[5.5, 6.0)	649	5.752	P50	113.78	113.94	0.16	0.16 cm
Boys HFA	[5.5, 6.0)	649	5.752	P97	123.27	123.06	-0.21	0.21 cm
Boys HFA	[6.0, 6.5)	1,432	6.248	P3	108.61	108.22	-0.39	0.39 cm
Boys HFA	[6.0, 6.5)	1,432	6.248	P50	116.95	116.76	-0.19	0.19 cm
Boys HFA	[6.0, 6.5)	1,432	6.248	P97	126.35	126.01	-0.35	0.35 cm
Boys HFA	[6.5, 7.0)	1,289	6.746	P3	110.11	110.81	0.71	0.71 cm
Boys HFA	[6.5, 7.0)	1,289	6.746	P50	119.35	119.50	0.15	0.15 cm
Boys HFA	[6.5, 7.0)	1,289	6.746	P97	128.74	128.86	0.12	0.12 cm
Boys HFA	[7.0, 7.5)	741	7.236	P3	113.08	113.31	0.23	0.23 cm
Boys HFA	[7.0, 7.5)	741	7.236	P50	122.18	122.14	-0.04	0.04 cm
Boys HFA	[7.0, 7.5)	741	7.236	P97	131.89	131.59	-0.30	0.30 cm
Boys HFA	[7.5, 8.0)	766	7.737	P3	114.78	115.81	1.03	1.03 cm
Boys HFA	[7.5, 8.0)	766	7.737	P50	125.38	124.79	-0.59	0.59 cm
Boys HFA	[7.5, 8.0)	766	7.737	P97	134.21	134.36	0.15	0.15 cm
Boys HFA	[8.0, 8.5)	780	8.233	P3	118.16	118.21	0.05	0.05 cm
Boys HFA	[8.0, 8.5)	780	8.233	P50	127.27	127.36	0.09	0.09 cm

Model	Age band	*N*	Prediction age (years)	Centile	Empirical	Model-based	Model - empirical	Absolute difference / unit
Boys HFA	[8.0, 8.5)	780	8.233	P97	137.93	137.07	-0.86	0.86 cm
Boys HFA	[8.5, 9.0)	896	8.756	P3	119.99	120.67	0.68	0.68 cm
Boys HFA	[8.5, 9.0)	896	8.756	P50	129.94	130.02	0.07	0.07 cm
Boys HFA	[8.5, 9.0)	896	8.756	P97	140.71	139.91	-0.80	0.80 cm
Boys HFA	[9.0, 9.5)	834	9.248	P3	123.48	122.89	-0.59	0.59 cm
Boys HFA	[9.0, 9.5)	834	9.248	P50	132.76	132.46	-0.30	0.30 cm
Boys HFA	[9.0, 9.5)	834	9.248	P97	142.40	142.56	0.15	0.15 cm
Boys HFA	[9.5, 10.0)	923	9.758	P3	124.99	125.09	0.10	0.10 cm
Boys HFA	[9.5, 10.0)	923	9.758	P50	134.89	134.94	0.05	0.05 cm
Boys HFA	[9.5, 10.0)	923	9.758	P97	145.82	145.30	-0.53	0.53 cm
Boys HFA	[10.0, 10.5)	934	10.237	P3	126.84	127.14	0.31	0.31 cm
Boys HFA	[10.0, 10.5)	934	10.237	P50	137.11	137.30	0.19	0.19 cm
Boys HFA	[10.0, 10.5)	934	10.237	P97	147.58	147.96	0.38	0.38 cm
Boys HFA	[10.5, 11.0)	952	10.749	P3	129.37	129.37	0.00	0.00 cm
Boys HFA	[10.5, 11.0)	952	10.749	P50	139.81	139.94	0.13	0.13 cm
Boys HFA	[10.5, 11.0)	952	10.749	P97	150.67	151.01	0.34	0.34 cm
Boys HFA	[11.0, 11.5)	1,043	11.255	P3	131.33	131.68	0.36	0.36 cm
Boys HFA	[11.0, 11.5)	1,043	11.255	P50	142.83	142.75	-0.07	0.07 cm
Boys HFA	[11.0, 11.5)	1,043	11.255	P97	154.10	154.33	0.23	0.23 cm
Boys HFA	[11.5, 12.0)	1,109	11.762	P3	134.39	134.15	-0.24	0.24 cm
Boys HFA	[11.5, 12.0)	1,109	11.762	P50	146.32	145.84	-0.49	0.49 cm
Boys HFA	[11.5, 12.0)	1,109	11.762	P97	158.00	158.05	0.05	0.05 cm
Boys HFA	[12.0, 12.5)	1,237	12.249	P3	136.14	136.67	0.53	0.53 cm
Boys HFA	[12.0, 12.5)	1,237	12.249	P50	148.63	148.99	0.37	0.37 cm
Boys HFA	[12.0, 12.5)	1,237	12.249	P97	162.62	161.86	-0.77	0.77 cm
Boys HFA	[12.5, 13.0)	1,135	12.747	P3	139.40	139.40	-0.01	0.01 cm
Boys HFA	[12.5, 13.0)	1,135	12.747	P50	152.08	152.31	0.23	0.23 cm
Boys HFA	[12.5, 13.0)	1,135	12.747	P97	166.10	165.76	-0.34	0.34 cm
Boys HFA	[13.0, 13.5)	1,030	13.240	P3	141.75	142.26	0.51	0.51 cm
Boys HFA	[13.0, 13.5)	1,030	13.240	P50	155.44	155.57	0.12	0.12 cm
Boys HFA	[13.0, 13.5)	1,030	13.240	P97	169.55	169.41	-0.14	0.14 cm
Boys HFA	[13.5, 14.0)	894	13.744	P3	145.27	145.38	0.11	0.11 cm
Boys HFA	[13.5, 14.0)	894	13.744	P50	158.75	158.78	0.03	0.03 cm
Boys HFA	[13.5, 14.0)	894	13.744	P97	172.59	172.68	0.09	0.09 cm
Boys HFA	[14.0, 14.5)	864	14.220	P3	148.27	148.47	0.20	0.20 cm
Boys HFA	[14.0, 14.5)	864	14.220	P50	161.82	161.63	-0.19	0.19 cm
Boys HFA	[14.0, 14.5)	864	14.220	P97	175.70	175.24	-0.46	0.46 cm
Boys HFA	[14.5, 15.0)	698	14.732	P3	151.16	151.77	0.61	0.61 cm
Boys HFA	[14.5, 15.0)	698	14.732	P50	163.81	164.44	0.62	0.62 cm

Model	Age band	*N*	Prediction age (years)	Centile	Empirical	Model-based	Model - empirical	Absolute difference / unit
Boys HFA	[14.5, 15.0)	698	14.732	P97	177.15	177.49	0.34	0.34 cm
Boys HFA	[15.0, 15.5)	586	15.231	P3	154.88	154.80	-0.08	0.08 cm
Boys HFA	[15.0, 15.5)	586	15.231	P50	166.86	166.87	0.00	0.00 cm
Boys HFA	[15.0, 15.5)	586	15.231	P97	179.62	179.27	-0.36	0.36 cm
Boys HFA	[15.5, 16.0)	419	15.737	P3	158.35	157.55	-0.80	0.80 cm
Boys HFA	[15.5, 16.0)	419	15.737	P50	168.91	168.99	0.07	0.07 cm
Boys HFA	[15.5, 16.0)	419	15.737	P97	181.65	180.70	-0.95	0.95 cm
Boys HFA	[16.0, 16.5)	324	16.235	P3	160.98	159.81	-1.17	1.17 cm
Boys HFA	[16.0, 16.5)	324	16.235	P50	170.99	170.68	-0.31	0.31 cm
Boys HFA	[16.0, 16.5)	324	16.235	P97	181.84	181.80	-0.05	0.05 cm
Boys HFA	[16.5, 17.0)	268	16.715	P3	161.43	161.59	0.16	0.16 cm
Boys HFA	[16.5, 17.0)	268	16.715	P50	172.13	171.99	-0.14	0.14 cm
Boys HFA	[16.5, 17.0)	268	16.715	P97	182.99	182.59	-0.40	0.40 cm
Boys HFA	[17.0, 17.5)	224	17.235	P3	163.66	163.15	-0.51	0.51 cm
Boys HFA	[17.0, 17.5)	224	17.235	P50	172.71	173.11	0.39	0.39 cm
Boys HFA	[17.0, 17.5)	224	17.235	P97	182.68	183.24	0.56	0.56 cm
Boys HFA	[17.5, 18.0]	145	17.749	P3	163.06	164.40	1.34	1.34 cm
Boys HFA	[17.5, 18.0]	145	17.749	P50	174.61	173.97	-0.64	0.64 cm
Boys HFA	[17.5, 18.0]	145	17.749	P97	184.36	183.70	-0.66	0.66 cm
Boys WFA	731-822 days	1,957	2.059	P3	10.80	10.84	0.04	0.04 kg
Boys WFA	731-822 days	1,957	2.059	P50	13.16	13.07	-0.09	0.09 kg
Boys WFA	731-822 days	1,957	2.059	P97	16.36	16.27	-0.09	0.09 kg
Boys WFA	823-913 days	586	2.363	P3	11.43	11.45	0.02	0.02 kg
Boys WFA	823-913 days	586	2.363	P50	13.83	13.84	0.01	0.01 kg
Boys WFA	823-913 days	586	2.363	P97	17.39	17.30	-0.10	0.10 kg
Boys WFA	914-1004 days	410	2.612	P3	11.91	11.93	0.01	0.01 kg
Boys WFA	914-1004 days	410	2.612	P50	14.58	14.45	-0.13	0.13 kg
Boys WFA	914-1004 days	410	2.612	P97	18.09	18.11	0.02	0.02 kg
Boys WFA	1005-1096 days	464	2.919	P3	12.51	12.48	-0.03	0.03 kg
Boys WFA	1005-1096 days	464	2.919	P50	15.13	15.16	0.03	0.03 kg
Boys WFA	1005-1096 days	464	2.919	P97	18.91	19.08	0.16	0.16 kg
Boys WFA	1097-1187 days	754	3.075	P3	12.83	12.75	-0.08	0.08 kg
Boys WFA	1097-1187 days	754	3.075	P50	15.64	15.51	-0.13	0.13 kg
Boys WFA	1097-1187 days	754	3.075	P97	19.65	19.55	-0.10	0.10 kg
Boys WFA	1188-1278 days	375	3.381	P3	13.39	13.25	-0.14	0.14 kg
Boys WFA	1188-1278 days	375	3.381	P50	16.23	16.16	-0.07	0.07 kg
Boys WFA	1188-1278 days	375	3.381	P97	20.81	20.43	-0.38	0.38 kg
Boys WFA	1279-1370 days	385	3.614	P3	13.49	13.62	0.13	0.13 kg
Boys WFA	1279-1370 days	385	3.614	P50	16.63	16.63	-0.00	0.00 kg

Model	Age band	*N*	Prediction age (years)	Centile	Empirical	Model-based	Model - empirical	Absolute difference / unit
Boys WFA	1279-1370 days	385	3.614	P97	21.32	21.09	-0.23	0.23 kg
Boys WFA	1371-1461 days	422	3.896	P3	13.64	14.06	0.42	0.42 kg
Boys WFA	1371-1461 days	422	3.896	P50	17.31	17.21	-0.10	0.10 kg
Boys WFA	1371-1461 days	422	3.896	P97	21.19	21.88	0.69	0.69 kg
Boys WFA	1462-1552 days	566	4.101	P3	14.48	14.39	-0.09	0.09 kg
Boys WFA	1462-1552 days	566	4.101	P50	17.57	17.63	0.06	0.06 kg
Boys WFA	1462-1552 days	566	4.101	P97	22.60	22.45	-0.16	0.16 kg
Boys WFA	1553-1644 days	387	4.386	P3	14.91	14.84	-0.07	0.07 kg
Boys WFA	1553-1644 days	387	4.386	P50	18.38	18.21	-0.17	0.17 kg
Boys WFA	1553-1644 days	387	4.386	P97	23.82	23.25	-0.58	0.58 kg
Boys WFA	1645-1735 days	381	4.635	P3	15.13	15.25	0.12	0.12 kg
Boys WFA	1645-1735 days	381	4.635	P50	18.93	18.73	-0.20	0.20 kg
Boys WFA	1645-1735 days	381	4.635	P97	24.03	23.95	-0.08	0.08 kg
Boys WFA	1736-1826 days	418	4.873	P3	15.55	15.63	0.08	0.08 kg
Boys WFA	1736-1826 days	418	4.873	P50	19.36	19.23	-0.14	0.14 kg
Boys WFA	1736-1826 days	418	4.873	P97	24.47	24.63	0.15	0.15 kg
Boys WFA	1827-1918 days	380	5.114	P3	16.18	16.02	-0.16	0.16 kg
Boys WFA	1827-1918 days	380	5.114	P50	19.69	19.73	0.03	0.03 kg
Boys WFA	1827-1918 days	380	5.114	P97	26.10	25.31	-0.79	0.79 kg
Boys WFA	1919-2009 days	281	5.374	P3	16.69	16.43	-0.26	0.26 kg
Boys WFA	1919-2009 days	281	5.374	P50	20.09	20.26	0.17	0.17 kg
Boys WFA	1919-2009 days	281	5.374	P97	25.60	26.05	0.45	0.45 kg
Boys WFA	2010-2100 days	317	5.626	P3	16.50	16.82	0.32	0.32 kg
Boys WFA	2010-2100 days	317	5.626	P50	20.75	20.78	0.03	0.03 kg
Boys WFA	2010-2100 days	317	5.626	P97	28.36	26.77	-1.59	1.59 kg
Boys WFA	2101-2191 days	327	5.878	P3	17.18	17.21	0.03	0.03 kg
Boys WFA	2101-2191 days	327	5.878	P50	21.02	21.29	0.27	0.27 kg
Boys WFA	2101-2191 days	327	5.878	P97	27.54	27.50	-0.04	0.04 kg
Boys WFA	2192-2283 days	735	6.127	P3	17.77	17.60	-0.18	0.18 kg
Boys WFA	2192-2283 days	735	6.127	P50	21.87	21.81	-0.06	0.06 kg
Boys WFA	2192-2283 days	735	6.127	P97	27.90	28.24	0.34	0.34 kg
Boys WFA	2284-2374 days	697	6.379	P3	18.08	17.99	-0.09	0.09 kg
Boys WFA	2284-2374 days	697	6.379	P50	22.30	22.34	0.04	0.04 kg
Boys WFA	2284-2374 days	697	6.379	P97	29.51	29.03	-0.49	0.49 kg
Boys WFA	2375-2465 days	650	6.612	P3	18.29	18.36	0.07	0.07 kg
Boys WFA	2375-2465 days	650	6.612	P50	22.91	22.85	-0.05	0.05 kg
Boys WFA	2375-2465 days	650	6.612	P97	29.28	29.79	0.51	0.51 kg
Boys WFA	2466-2557 days	642	6.877	P3	18.60	18.79	0.19	0.19 kg
Boys WFA	2466-2557 days	642	6.877	P50	23.41	23.46	0.05	0.05 kg

Model	Age band	*N*	Prediction age (years)	Centile	Empirical	Model-based	Model - empirical	Absolute difference / unit
Boys WFA	2466-2557 days	642	6.877	P97	30.73	30.70	-0.03	0.03 kg
Boys WFA	2558-2648 days	385	7.132	P3	19.74	19.21	-0.52	0.52 kg
Boys WFA	2558-2648 days	385	7.132	P50	24.30	24.06	-0.24	0.24 kg
Boys WFA	2558-2648 days	385	7.132	P97	32.75	31.64	-1.11	1.11 kg
Boys WFA	2649-2739 days	353	7.368	P3	19.28	19.61	0.33	0.33 kg
Boys WFA	2649-2739 days	353	7.368	P50	24.19	24.64	0.46	0.46 kg
Boys WFA	2649-2739 days	353	7.368	P97	32.39	32.56	0.17	0.17 kg
Boys WFA	2740-2831 days	402	7.639	P3	19.79	20.08	0.29	0.29 kg
Boys WFA	2740-2831 days	402	7.639	P50	25.53	25.34	-0.19	0.19 kg
Boys WFA	2740-2831 days	402	7.639	P97	33.80	33.69	-0.11	0.11 kg
Boys WFA	2832-2922 days	367	7.874	P3	20.78	20.50	-0.27	0.27 kg
Boys WFA	2832-2922 days	367	7.874	P50	26.07	25.97	-0.11	0.11 kg
Boys WFA	2832-2922 days	367	7.874	P97	34.96	34.73	-0.24	0.24 kg
Boys WFA	2923-3013 days	414	8.118	P3	21.03	20.94	-0.08	0.08 kg
Boys WFA	2923-3013 days	414	8.118	P50	26.48	26.64	0.16	0.16 kg
Boys WFA	2923-3013 days	414	8.118	P97	35.25	35.86	0.60	0.60 kg
Boys WFA	3014-3105 days	371	8.389	P3	21.24	21.44	0.20	0.20 kg
Boys WFA	3014-3105 days	371	8.389	P50	27.29	27.41	0.13	0.13 kg
Boys WFA	3014-3105 days	371	8.389	P97	37.95	37.19	-0.76	0.76 kg
Boys WFA	3106-3196 days	432	8.630	P3	21.40	21.89	0.49	0.49 kg
Boys WFA	3106-3196 days	432	8.630	P50	27.87	28.12	0.25	0.25 kg
Boys WFA	3106-3196 days	432	8.630	P97	39.54	38.44	-1.10	1.10 kg
Boys WFA	3197-3287 days	456	8.884	P3	22.44	22.37	-0.07	0.07 kg
Boys WFA	3197-3287 days	456	8.884	P50	28.50	28.90	0.40	0.40 kg
Boys WFA	3197-3287 days	456	8.884	P97	39.61	39.83	0.22	0.22 kg
Boys WFA	3288-3379 days	427	9.125	P3	23.80	22.82	-0.98	0.98 kg
Boys WFA	3288-3379 days	427	9.125	P50	29.68	29.64	-0.04	0.04 kg
Boys WFA	3288-3379 days	427	9.125	P97	41.40	41.20	-0.20	0.20 kg
Boys WFA	3380-3470 days	418	9.380	P3	23.38	23.30	-0.08	0.08 kg
Boys WFA	3380-3470 days	418	9.380	P50	30.64	30.43	-0.21	0.21 kg
Boys WFA	3380-3470 days	418	9.380	P97	41.75	42.70	0.96	0.96 kg
Boys WFA	3471-3561 days	437	9.626	P3	23.42	23.75	0.33	0.33 kg
Boys WFA	3471-3561 days	437	9.626	P50	31.79	31.21	-0.58	0.58 kg
Boys WFA	3471-3561 days	437	9.626	P97	41.42	44.22	2.80	2.80 kg
Boys WFA	3562-3652 days	475	9.884	P3	23.98	24.21	0.24	0.24 kg
Boys WFA	3562-3652 days	475	9.884	P50	32.01	32.02	0.01	0.01 kg
Boys WFA	3562-3652 days	475	9.884	P97	49.74	45.85	-3.88	3.88 kg
Boys BMIFA	[2.0, 2.5)	2,543	2.094	P3	14.98	15.01	0.03	0.03 kg/m^2
Boys BMIFA	[2.0, 2.5)	2,543	2.094	P50	16.84	16.84	0.01	0.01 kg/m^2

Model	Age band	*N*	Prediction age (years)	Centile	Empirical	Model-based	Model - empirical	Absolute difference / unit
Boys BMIFA	[2.0, 2.5)	2,543	2.094	P97	20.02	20.02	-0.00	0.00 kg/m^2
Boys BMIFA	[2.5, 3.0)	830	2.757	P3	14.78	14.82	0.04	0.04 kg/m^2
Boys BMIFA	[2.5, 3.0)	830	2.757	P50	16.66	16.67	0.01	0.01 kg/m^2
Boys BMIFA	[2.5, 3.0)	830	2.757	P97	19.68	19.91	0.24	0.24 kg/m^2
Boys BMIFA	[3.0, 3.5)	1,173	3.146	P3	14.70	14.71	0.01	0.01 kg/m^2
Boys BMIFA	[3.0, 3.5)	1,173	3.146	P50	16.63	16.58	-0.05	0.05 kg/m^2
Boys BMIFA	[3.0, 3.5)	1,173	3.146	P97	19.79	19.86	0.07	0.07 kg/m^2
Boys BMIFA	[3.5, 4.0)	787	3.759	P3	14.70	14.55	-0.16	0.16 kg/m^2
Boys BMIFA	[3.5, 4.0)	787	3.759	P50	16.40	16.45	0.05	0.05 kg/m^2
Boys BMIFA	[3.5, 4.0)	787	3.759	P97	19.77	19.79	0.02	0.02 kg/m^2
Boys BMIFA	[4.0, 4.5)	970	4.189	P3	14.34	14.43	0.09	0.09 kg/m^2
Boys BMIFA	[4.0, 4.5)	970	4.189	P50	16.31	16.36	0.05	0.05 kg/m^2
Boys BMIFA	[4.0, 4.5)	970	4.189	P97	20.10	19.75	-0.34	0.34 kg/m^2
Boys BMIFA	[4.5, 5.0)	802	4.758	P3	14.34	14.27	-0.07	0.07 kg/m^2
Boys BMIFA	[4.5, 5.0)	802	4.758	P50	16.28	16.24	-0.04	0.04 kg/m^2
Boys BMIFA	[4.5, 5.0)	802	4.758	P97	19.87	19.74	-0.14	0.14 kg/m^2
Boys BMIFA	[5.0, 5.5)	656	5.216	P3	14.22	14.14	-0.08	0.08 kg/m^2
Boys BMIFA	[5.0, 5.5)	656	5.216	P50	16.22	16.16	-0.06	0.06 kg/m^2
Boys BMIFA	[5.0, 5.5)	656	5.216	P97	19.95	19.77	-0.18	0.18 kg/m^2
Boys BMIFA	[5.5, 6.0)	649	5.752	P3	13.96	14.01	0.05	0.05 kg/m^2
Boys BMIFA	[5.5, 6.0)	649	5.752	P50	16.10	16.11	0.00	0.00 kg/m^2
Boys BMIFA	[5.5, 6.0)	649	5.752	P97	20.52	19.89	-0.63	0.63 kg/m^2
Boys BMIFA	[6.0, 6.5)	1,432	6.248	P3	13.97	13.93	-0.05	0.05 kg/m^2
Boys BMIFA	[6.0, 6.5)	1,432	6.248	P50	16.10	16.11	0.00	0.00 kg/m^2
Boys BMIFA	[6.0, 6.5)	1,432	6.248	P97	20.27	20.10	-0.17	0.17 kg/m^2
Boys BMIFA	[6.5, 7.0)	1,289	6.746	P3	13.84	13.87	0.02	0.02 kg/m^2
Boys BMIFA	[6.5, 7.0)	1,289	6.746	P50	16.20	16.15	-0.05	0.05 kg/m^2
Boys BMIFA	[6.5, 7.0)	1,289	6.746	P97	20.20	20.40	0.19	0.19 kg/m^2
Boys BMIFA	[7.0, 7.5)	741	7.236	P3	13.86	13.84	-0.02	0.02 kg/m^2
Boys BMIFA	[7.0, 7.5)	741	7.236	P50	16.23	16.23	0.01	0.01 kg/m^2
Boys BMIFA	[7.0, 7.5)	741	7.236	P97	20.51	20.77	0.26	0.26 kg/m^2
Boys BMIFA	[7.5, 8.0)	766	7.737	P3	13.81	13.84	0.03	0.03 kg/m^2
Boys BMIFA	[7.5, 8.0)	766	7.737	P50	16.39	16.36	-0.02	0.02 kg/m^2
Boys BMIFA	[7.5, 8.0)	766	7.737	P97	21.71	21.23	-0.48	0.48 kg/m^2
Boys BMIFA	[8.0, 8.5)	780	8.233	P3	13.92	13.87	-0.05	0.05 kg/m^2
Boys BMIFA	[8.0, 8.5)	780	8.233	P50	16.44	16.53	0.09	0.09 kg/m^2
Boys BMIFA	[8.0, 8.5)	780	8.233	P97	21.87	21.75	-0.12	0.12 kg/m^2
Boys BMIFA	[8.5, 9.0)	896	8.756	P3	14.03	13.92	-0.11	0.11 kg/m^2
Boys BMIFA	[8.5, 9.0)	896	8.756	P50	16.62	16.73	0.11	0.11 kg/m^2

Model	Age band	*N*	Prediction age (years)	Centile	Empirical	Model-based	Model - empirical	Absolute difference / unit
Boys BMIFA	[8.5, 9.0)	896	8.756	P97	22.21	22.36	0.16	0.16 kg/m^2
Boys BMIFA	[9.0, 9.5)	834	9.248	P3	14.21	13.99	-0.22	0.22 kg/m^2
Boys BMIFA	[9.0, 9.5)	834	9.248	P50	17.11	16.95	-0.16	0.16 kg/m^2
Boys BMIFA	[9.0, 9.5)	834	9.248	P97	23.15	22.97	-0.18	0.18 kg/m^2
Boys BMIFA	[9.5, 10.0)	923	9.758	P3	14.04	14.09	0.05	0.05 kg/m^2
Boys BMIFA	[9.5, 10.0)	923	9.758	P50	17.38	17.21	-0.17	0.17 kg/m^2
Boys BMIFA	[9.5, 10.0)	923	9.758	P97	24.22	23.62	-0.60	0.60 kg/m^2
Boys BMIFA	[10.0, 10.5)	934	10.237	P3	14.09	14.21	0.12	0.12 kg/m^2
Boys BMIFA	[10.0, 10.5)	934	10.237	P50	17.46	17.47	0.01	0.01 kg/m^2
Boys BMIFA	[10.0, 10.5)	934	10.237	P97	24.41	24.22	-0.19	0.19 kg/m^2
Boys BMIFA	[10.5, 11.0)	952	10.749	P3	14.39	14.36	-0.03	0.03 kg/m^2
Boys BMIFA	[10.5, 11.0)	952	10.749	P50	17.73	17.76	0.04	0.04 kg/m^2
Boys BMIFA	[10.5, 11.0)	952	10.749	P97	24.74	24.86	0.11	0.11 kg/m^2
Boys BMIFA	[11.0, 11.5)	1,043	11.255	P3	14.55	14.53	-0.02	0.02 kg/m^2
Boys BMIFA	[11.0, 11.5)	1,043	11.255	P50	18.01	18.08	0.07	0.07 kg/m^2
Boys BMIFA	[11.0, 11.5)	1,043	11.255	P97	25.95	25.46	-0.48	0.48 kg/m^2
Boys BMIFA	[11.5, 12.0)	1,109	11.762	P3	14.78	14.73	-0.05	0.05 kg/m^2
Boys BMIFA	[11.5, 12.0)	1,109	11.762	P50	18.40	18.42	0.02	0.02 kg/m^2
Boys BMIFA	[11.5, 12.0)	1,109	11.762	P97	26.21	26.04	-0.17	0.17 kg/m^2
Boys BMIFA	[12.0, 12.5)	1,237	12.249	P3	14.87	14.95	0.08	0.08 kg/m^2
Boys BMIFA	[12.0, 12.5)	1,237	12.249	P50	18.72	18.76	0.04	0.04 kg/m^2
Boys BMIFA	[12.0, 12.5)	1,237	12.249	P97	26.29	26.56	0.27	0.27 kg/m^2
Boys BMIFA	[12.5, 13.0)	1,135	12.747	P3	15.28	15.19	-0.09	0.09 kg/m^2
Boys BMIFA	[12.5, 13.0)	1,135	12.747	P50	19.22	19.11	-0.10	0.10 kg/m^2
Boys BMIFA	[12.5, 13.0)	1,135	12.747	P97	27.68	27.05	-0.63	0.63 kg/m^2
Boys BMIFA	[13.0, 13.5)	1,030	13.240	P3	15.58	15.44	-0.14	0.14 kg/m^2
Boys BMIFA	[13.0, 13.5)	1,030	13.240	P50	19.47	19.48	0.00	0.00 kg/m^2
Boys BMIFA	[13.0, 13.5)	1,030	13.240	P97	27.96	27.48	-0.48	0.48 kg/m^2
Boys BMIFA	[13.5, 14.0)	894	13.744	P3	15.54	15.72	0.18	0.18 kg/m^2
Boys BMIFA	[13.5, 14.0)	894	13.744	P50	19.74	19.84	0.10	0.10 kg/m^2
Boys BMIFA	[13.5, 14.0)	894	13.744	P97	29.63	27.87	-1.75	1.75 kg/m^2
Boys BMIFA	[14.0, 14.5)	864	14.220	P3	15.74	15.99	0.25	0.25 kg/m^2
Boys BMIFA	[14.0, 14.5)	864	14.220	P50	19.99	20.19	0.20	0.20 kg/m^2
Boys BMIFA	[14.0, 14.5)	864	14.220	P97	28.67	28.19	-0.48	0.48 kg/m^2
Boys BMIFA	[14.5, 15.0)	698	14.732	P3	16.49	16.29	-0.19	0.19 kg/m^2
Boys BMIFA	[14.5, 15.0)	698	14.732	P50	20.56	20.55	-0.00	0.00 kg/m^2
Boys BMIFA	[14.5, 15.0)	698	14.732	P97	28.13	28.48	0.34	0.34 kg/m^2
Boys BMIFA	[15.0, 15.5)	586	15.231	P3	16.98	16.59	-0.39	0.39 kg/m^2
Boys BMIFA	[15.0, 15.5)	586	15.231	P50	21.03	20.90	-0.13	0.13 kg/m^2

**Model**	**Age band**	***N***	**Prediction age (years)**	**Centile**	**Empirical**	**Model-based**	**Model - empirical**	**Absolute difference / unit**
Boys BMIFA	[15.0, 15.5)	586	15.231	P97	29.07	28.71	-0.36	0.36 kg/m^2
Boys BMIFA	[15.5, 16.0)	419	15.737	P3	16.97	16.89	-0.07	0.07 kg/m^2
Boys BMIFA	[15.5, 16.0)	419	15.737	P50	20.99	21.24	0.26	0.26 kg/m^2
Boys BMIFA	[15.5, 16.0)	419	15.737	P97	28.86	28.92	0.06	0.06 kg/m^2
Boys BMIFA	[16.0, 16.5)	324	16.235	P3	17.32	17.19	-0.13	0.13 kg/m^2
Boys BMIFA	[16.0, 16.5)	324	16.235	P50	21.36	21.57	0.20	0.20 kg/m^2
Boys BMIFA	[16.0, 16.5)	324	16.235	P97	30.37	29.11	-1.27	1.27 kg/m^2
Boys BMIFA	[16.5, 17.0)	268	16.715	P3	17.35	17.48	0.12	0.12 kg/m^2
Boys BMIFA	[16.5, 17.0)	268	16.715	P50	22.04	21.87	-0.17	0.17 kg/m^2
Boys BMIFA	[16.5, 17.0)	268	16.715	P97	29.27	29.27	0.01	0.01 kg/m^2
Boys BMIFA	[17.0, 17.5)	224	17.235	P3	17.77	17.78	0.01	0.01 kg/m^2
Boys BMIFA	[17.0, 17.5)	224	17.235	P50	21.93	22.19	0.27	0.27 kg/m^2
Boys BMIFA	[17.0, 17.5)	224	17.235	P97	30.46	29.44	-1.02	1.02 kg/m^2
Boys BMIFA	[17.5, 18.0]	145	17.749	P3	17.52	18.08	0.56	0.56 kg/m^2
Boys BMIFA	[17.5, 18.0]	145	17.749	P50	22.58	22.50	-0.08	0.08 kg/m^2
Boys BMIFA	[17.5, 18.0]	145	17.749	P97	29.61	29.60	-0.01	0.01 kg/m^2
Girls HFA	[2.0, 2.5)	2,419	2.250	P3	80.06	80.71	0.65	0.65 cm
Girls HFA	[2.0, 2.5)	2,419	2.250	P50	86.99	87.95	0.96	0.96 cm
Girls HFA	[2.0, 2.5)	2,419	2.250	P97	94.66	95.36	0.70	0.70 cm
Girls HFA	[2.5, 3.0)	818	2.750	P3	86.58	86.37	-0.21	0.21 cm
Girls HFA	[2.5, 3.0)	818	2.750	P50	93.77	94.13	0.36	0.36 cm
Girls HFA	[2.5, 3.0)	818	2.750	P97	102.53	102.29	-0.24	0.24 cm
Girls HFA	[3.0, 3.5)	1,165	3.250	P3	88.35	88.83	0.48	0.48 cm
Girls HFA	[3.0, 3.5)	1,165	3.250	P50	96.74	96.94	0.20	0.20 cm
Girls HFA	[3.0, 3.5)	1,165	3.250	P97	104.86	105.64	0.78	0.78 cm
Girls HFA	[3.5, 4.0)	639	3.750	P3	90.74	91.70	0.95	0.95 cm
Girls HFA	[3.5, 4.0)	639	3.750	P50	100.39	100.15	-0.24	0.24 cm
Girls HFA	[3.5, 4.0)	639	3.750	P97	108.87	109.34	0.48	0.48 cm
Girls HFA	[4.0, 4.5)	961	4.250	P3	95.44	95.09	-0.35	0.35 cm
Girls HFA	[4.0, 4.5)	961	4.250	P50	103.27	103.63	0.36	0.36 cm
Girls HFA	[4.0, 4.5)	961	4.250	P97	112.52	112.97	0.46	0.46 cm
Girls HFA	[4.5, 5.0)	729	4.750	P3	97.51	98.18	0.67	0.67 cm
Girls HFA	[4.5, 5.0)	729	4.750	P50	107.17	106.95	-0.22	0.22 cm
Girls HFA	[4.5, 5.0)	729	4.750	P97	116.60	116.58	-0.02	0.02 cm
Girls HFA	[5.0, 5.5)	627	5.250	P3	100.70	100.72	0.03	0.03 cm
Girls HFA	[5.0, 5.5)	627	5.250	P50	109.43	109.80	0.37	0.37 cm
Girls HFA	[5.0, 5.5)	627	5.250	P97	119.98	119.80	-0.18	0.18 cm
Girls HFA	[5.5, 6.0)	659	5.750	P3	102.17	102.92	0.75	0.75 cm
Girls HFA	[5.5, 6.0)	659	5.750	P50	112.69	112.32	-0.37	0.37 cm

Model	Age band	*N*	Prediction age (years)	Centile	Empirical	Model-based	Model - empirical	Absolute difference / unit
Girls HFA	[5.5, 6.0)	659	5.750	P97	122.50	122.70	0.19	0.19 cm
Girls HFA	[6.0, 6.5)	1,451	6.250	P3	104.54	105.37	0.83	0.83 cm
Girls HFA	[6.0, 6.5)	1,451	6.250	P50	114.76	115.09	0.33	0.33 cm
Girls HFA	[6.0, 6.5)	1,451	6.250	P97	125.80	125.81	0.01	0.01 cm
Girls HFA	[6.5, 7.0)	1,383	6.750	P3	109.13	108.29	-0.84	0.84 cm
Girls HFA	[6.5, 7.0)	1,383	6.750	P50	118.99	118.33	-0.66	0.66 cm
Girls HFA	[6.5, 7.0)	1,383	6.750	P97	130.17	129.37	-0.80	0.80 cm
Girls HFA	[7.0, 7.5)	922	7.250	P3	110.56	110.87	0.31	0.31 cm
Girls HFA	[7.0, 7.5)	922	7.250	P50	120.91	121.23	0.32	0.32 cm
Girls HFA	[7.0, 7.5)	922	7.250	P97	132.17	132.57	0.40	0.40 cm
Girls HFA	[7.5, 8.0)	965	7.750	P3	113.36	113.02	-0.35	0.35 cm
Girls HFA	[7.5, 8.0)	965	7.750	P50	124.23	123.74	-0.48	0.48 cm
Girls HFA	[7.5, 8.0)	965	7.750	P97	135.22	135.44	0.21	0.21 cm
Girls HFA	[8.0, 8.5)	1,089	8.250	P3	114.71	115.20	0.48	0.48 cm
Girls HFA	[8.0, 8.5)	1,089	8.250	P50	126.01	126.39	0.38	0.38 cm
Girls HFA	[8.0, 8.5)	1,089	8.250	P97	137.93	138.53	0.60	0.60 cm
Girls HFA	[8.5, 9.0)	1,184	8.750	P3	117.94	117.51	-0.43	0.43 cm
Girls HFA	[8.5, 9.0)	1,184	8.750	P50	130.32	129.23	-1.09	1.09 cm
Girls HFA	[8.5, 9.0)	1,184	8.750	P97	142.08	141.89	-0.20	0.20 cm
Girls HFA	[9.0, 9.5)	1,329	9.250	P3	119.20	119.87	0.66	0.66 cm
Girls HFA	[9.0, 9.5)	1,329	9.250	P50	131.22	132.13	0.91	0.91 cm
Girls HFA	[9.0, 9.5)	1,329	9.250	P97	145.45	145.30	-0.14	0.14 cm
Girls HFA	[9.5, 10.0)	1,294	9.750	P3	122.64	122.32	-0.32	0.32 cm
Girls HFA	[9.5, 10.0)	1,294	9.750	P50	135.70	135.09	-0.61	0.61 cm
Girls HFA	[9.5, 10.0)	1,294	9.750	P97	148.56	148.73	0.17	0.17 cm
Girls HFA	[10.0, 10.5)	1,490	10.250	P3	124.54	125.03	0.49	0.49 cm
Girls HFA	[10.0, 10.5)	1,490	10.250	P50	137.80	138.20	0.40	0.40 cm
Girls HFA	[10.0, 10.5)	1,490	10.250	P97	151.83	152.16	0.33	0.33 cm
Girls HFA	[10.5, 11.0)	1,442	10.750	P3	127.95	128.03	0.08	0.08 cm
Girls HFA	[10.5, 11.0)	1,442	10.750	P50	141.96	141.44	-0.52	0.52 cm
Girls HFA	[10.5, 11.0)	1,442	10.750	P97	156.14	155.54	-0.60	0.60 cm
Girls HFA	[11.0, 11.5)	1,505	11.250	P3	131.25	131.24	-0.01	0.01 cm
Girls HFA	[11.0, 11.5)	1,505	11.250	P50	144.02	144.67	0.65	0.65 cm
Girls HFA	[11.0, 11.5)	1,505	11.250	P97	159.19	158.68	-0.51	0.51 cm
Girls HFA	[11.5, 12.0)	1,400	11.750	P3	133.56	134.48	0.92	0.92 cm
Girls HFA	[11.5, 12.0)	1,400	11.750	P50	147.58	147.79	0.21	0.21 cm
Girls HFA	[11.5, 12.0)	1,400	11.750	P97	161.67	161.53	-0.14	0.14 cm
Girls HFA	[12.0, 12.5)	1,349	12.250	P3	137.82	137.56	-0.26	0.26 cm
Girls HFA	[12.0, 12.5)	1,349	12.250	P50	150.46	150.62	0.16	0.16 cm

Model	Age band	*N*	Prediction age (years)	Centile	Empirical	Model-based	Model - empirical	Absolute difference / unit
Girls HFA	[12.0, 12.5)	1,349	12.250	P97	164.69	164.00	-0.69	0.69 cm
Girls HFA	[12.5, 13.0)	1,129	12.750	P3	139.82	140.26	0.44	0.44 cm
Girls HFA	[12.5, 13.0)	1,129	12.750	P50	152.86	153.01	0.15	0.15 cm
Girls HFA	[12.5, 13.0)	1,129	12.750	P97	166.07	165.98	-0.09	0.09 cm
Girls HFA	[13.0, 13.5)	881	13.250	P3	141.69	142.45	0.76	0.76 cm
Girls HFA	[13.0, 13.5)	881	13.250	P50	154.51	154.86	0.35	0.35 cm
Girls HFA	[13.0, 13.5)	881	13.250	P97	167.82	167.40	-0.43	0.43 cm
Girls HFA	[13.5, 14.0)	731	13.750	P3	144.67	144.15	-0.52	0.52 cm
Girls HFA	[13.5, 14.0)	731	13.750	P50	156.13	156.22	0.09	0.09 cm
Girls HFA	[13.5, 14.0)	731	13.750	P97	168.24	168.33	0.09	0.09 cm
Girls HFA	[14.0, 14.5)	541	14.250	P3	144.66	145.46	0.80	0.80 cm
Girls HFA	[14.0, 14.5)	541	14.250	P50	157.29	157.20	-0.09	0.09 cm
Girls HFA	[14.0, 14.5)	541	14.250	P97	168.70	168.91	0.21	0.21 cm
Girls HFA	[14.5, 15.0)	419	14.750	P3	147.18	146.45	-0.73	0.73 cm
Girls HFA	[14.5, 15.0)	419	14.750	P50	157.74	157.89	0.15	0.15 cm
Girls HFA	[14.5, 15.0)	419	14.750	P97	168.83	169.24	0.41	0.41 cm
Girls HFA	[15.0, 15.5)	440	15.250	P3	146.74	147.18	0.44	0.44 cm
Girls HFA	[15.0, 15.5)	440	15.250	P50	158.54	158.36	-0.19	0.19 cm
Girls HFA	[15.0, 15.5)	440	15.250	P97	169.32	169.40	0.07	0.07 cm
Girls HFA	[15.5, 16.0)	506	15.750	P3	146.30	147.69	1.39	1.39 cm
Girls HFA	[15.5, 16.0)	506	15.750	P50	158.90	158.65	-0.25	0.25 cm
Girls HFA	[15.5, 16.0)	506	15.750	P97	170.88	169.43	-1.45	1.45 cm
Girls HFA	[16.0, 16.5)	682	16.250	P3	148.97	148.02	-0.95	0.95 cm
Girls HFA	[16.0, 16.5)	682	16.250	P50	158.72	158.81	0.10	0.10 cm
Girls HFA	[16.0, 16.5)	682	16.250	P97	168.69	169.37	0.68	0.68 cm
Girls HFA	[16.5, 17.0)	929	16.750	P3	148.04	148.22	0.18	0.18 cm
Girls HFA	[16.5, 17.0)	929	16.750	P50	158.66	158.87	0.21	0.21 cm
Girls HFA	[16.5, 17.0)	929	16.750	P97	169.67	169.25	-0.41	0.41 cm
Girls HFA	[17.0, 17.5)	1,134	17.250	P3	148.73	148.33	-0.40	0.40 cm
Girls HFA	[17.0, 17.5)	1,134	17.250	P50	158.86	158.87	0.01	0.01 cm
Girls HFA	[17.0, 17.5)	1,134	17.250	P97	168.62	169.10	0.49	0.49 cm
Girls HFA	[17.5, 18.0)	1,452	17.750	P3	148.32	148.39	0.07	0.07 cm
Girls HFA	[17.5, 18.0)	1,452	17.750	P50	158.53	158.84	0.31	0.31 cm
Girls HFA	[17.5, 18.0)	1,452	17.750	P97	169.04	168.95	-0.08	0.08 cm
Girls WFA	731.0000-822.3125 days	1,822	2.127	P3	10.32	10.45	0.14	0.14 kg
Girls WFA	731.0000-822.3125 days	1,822	2.127	P50	12.61	12.74	0.13	0.13 kg
Girls WFA	731.0000-822.3125 days	1,822	2.127	P97	15.51	15.82	0.31	0.31 kg

Model	Age band	*N*	Prediction age (years)	Centile	Empirical	Model-based	Model - empirical	Absolute difference / unit
Girls WFA	822.3125-913.6250 days	542	2.376	P3	10.89	10.82	-0.07	0.07 kg
Girls WFA	822.3125-913.6250 days	542	2.376	P50	13.27	13.25	-0.02	0.02 kg
Girls WFA	822.3125-913.6250 days	542	2.376	P97	16.35	16.54	0.19	0.19 kg
Girls WFA	913.6250-1004.9375 days	397	2.626	P3	12.01	11.79	-0.22	0.22 kg
Girls WFA	913.6250-1004.9375 days	397	2.626	P50	14.55	14.43	-0.11	0.11 kg
Girls WFA	913.6250-1004.9375 days	397	2.626	P97	18.13	18.02	-0.12	0.12 kg
Girls WFA	1004.9375-1096.2500 days	427	2.877	P3	12.30	12.24	-0.05	0.05 kg
Girls WFA	1004.9375-1096.2500 days	427	2.877	P50	14.89	15.00	0.11	0.11 kg
Girls WFA	1004.9375-1096.2500 days	427	2.877	P97	18.71	18.75	0.03	0.03 kg
Girls WFA	1096.2500-1187.5625 days	757	3.127	P3	12.38	12.47	0.09	0.09 kg
Girls WFA	1096.2500-1187.5625 days	757	3.127	P50	15.33	15.32	-0.01	0.01 kg
Girls WFA	1096.2500-1187.5625 days	757	3.127	P97	19.50	19.21	-0.30	0.30 kg
Girls WFA	1187.5625-1278.8750 days	332	3.376	P3	12.38	12.71	0.33	0.33 kg
Girls WFA	1187.5625-1278.8750 days	332	3.376	P50	15.60	15.66	0.06	0.06 kg
Girls WFA	1187.5625-1278.8750 days	332	3.376	P97	20.10	19.69	-0.41	0.41 kg
Girls WFA	1278.8750-1370.1875 days	301	3.628	P3	13.24	13.11	-0.13	0.13 kg
Girls WFA	1278.8750-1370.1875 days	301	3.628	P50	16.26	16.18	-0.08	0.08 kg
Girls WFA	1278.8750-1370.1875 days	301	3.628	P97	20.00	20.38	0.38	0.38 kg
Girls WFA	1370.1875-1461.5000 days	331	3.877	P3	13.24	13.53	0.28	0.28 kg
Girls WFA	1370.1875-1461.5000 days	331	3.877	P50	16.65	16.70	0.06	0.06 kg
Girls WFA	1370.1875-1461.5000 days	331	3.877	P97	21.09	21.07	-0.02	0.02 kg
Girls WFA	1461.5000-1552.8125 days	534	4.126	P3	14.00	13.99	-0.01	0.01 kg
Girls WFA	1461.5000-1552.8125 days	534	4.126	P50	17.15	17.30	0.15	0.15 kg
Girls WFA	1461.5000-1552.8125 days	534	4.126	P97	21.69	21.86	0.17	0.17 kg

**Model**	**Age band**	***N***	**Prediction age (years)**	**Centile**	**Empirical**	**Model-based**	**Model - empirical**	**Absolute difference / unit**
Girls WFA	1552.8125-1644.1250 days	376	4.375	P3	14.10	14.34	0.25	0.25 kg
Girls WFA	1552.8125-1644.1250 days	376	4.375	P50	17.81	17.77	-0.04	0.04 kg
Girls WFA	1552.8125-1644.1250 days	376	4.375	P97	22.41	22.51	0.09	0.09 kg
Girls WFA	1644.1250-1735.4375 days	355	4.627	P3	14.43	14.71	0.28	0.28 kg
Girls WFA	1644.1250-1735.4375 days	355	4.627	P50	18.23	18.26	0.03	0.03 kg
Girls WFA	1644.1250-1735.4375 days	355	4.627	P97	23.28	23.16	-0.12	0.12 kg
Girls WFA	1735.4375-1826.7500 days	342	4.876	P3	15.32	15.14	-0.18	0.18 kg
Girls WFA	1735.4375-1826.7500 days	342	4.876	P50	18.88	18.82	-0.06	0.06 kg
Girls WFA	1735.4375-1826.7500 days	342	4.876	P97	23.40	23.91	0.51	0.51 kg
Girls WFA	1826.7500-1918.0625 days	324	5.125	P3	15.31	15.47	0.16	0.16 kg
Girls WFA	1826.7500-1918.0625 days	324	5.125	P50	19.34	19.26	-0.08	0.08 kg
Girls WFA	1826.7500-1918.0625 days	324	5.125	P97	24.50	24.52	0.02	0.02 kg
Girls WFA	1918.0625-2009.3750 days	282	5.377	P3	15.97	15.79	-0.18	0.18 kg
Girls WFA	1918.0625-2009.3750 days	282	5.377	P50	19.70	19.70	0.00	0.00 kg
Girls WFA	1918.0625-2009.3750 days	282	5.377	P97	24.83	25.16	0.33	0.33 kg
Girls WFA	2009.3750-2100.6875 days	275	5.626	P3	16.34	16.16	-0.19	0.19 kg
Girls WFA	2009.3750-2100.6875 days	275	5.626	P50	20.08	20.23	0.16	0.16 kg
Girls WFA	2009.3750-2100.6875 days	275	5.626	P97	25.08	25.96	0.87	0.87 kg
Girls WFA	2100.6875-2192.0000 days	347	5.875	P3	16.50	16.46	-0.04	0.04 kg
Girls WFA	2100.6875-2192.0000 days	347	5.875	P50	20.85	20.69	-0.17	0.17 kg
Girls WFA	2100.6875-2192.0000 days	347	5.875	P97	27.01	26.66	-0.35	0.35 kg
Girls WFA	2192.0000-2283.3125 days	482	6.127	P3	16.73	16.68	-0.04	0.04 kg
Girls WFA	2192.0000-2283.3125 days	482	6.127	P50	21.25	21.04	-0.21	0.21 kg
Girls WFA	2192.0000-2283.3125 days	482	6.127	P97	27.67	27.23	-0.44	0.44 kg

Model	Age band	*N*	Prediction age (years)	Centile	Empirical	Model-based	Model - empirical	Absolute difference / unit
Girls WFA	2283.3125-2374.6250 days	436	6.376	P3	17.04	17.05	0.01	0.01 kg
Girls WFA	2283.3125-2374.6250 days	436	6.376	P50	21.21	21.56	0.35	0.35 kg
Girls WFA	2283.3125-2374.6250 days	436	6.376	P97	28.80	27.98	-0.82	0.82 kg
Girls WFA	2374.6250-2465.9375 days	432	6.626	P3	17.74	17.59	-0.14	0.14 kg
Girls WFA	2374.6250-2465.9375 days	432	6.626	P50	22.29	22.29	-0.00	0.00 kg
Girls WFA	2374.6250-2465.9375 days	432	6.626	P97	28.96	28.99	0.03	0.03 kg
Girls WFA	2465.9375-2557.2500 days	453	6.877	P3	18.15	18.13	-0.02	0.02 kg
Girls WFA	2465.9375-2557.2500 days	453	6.877	P50	23.37	23.00	-0.37	0.37 kg
Girls WFA	2465.9375-2557.2500 days	453	6.877	P97	29.49	29.98	0.49	0.49 kg
Girls WFA	2557.2500-2648.5625 days	455	7.127	P3	18.80	18.55	-0.25	0.25 kg
Girls WFA	2557.2500-2648.5625 days	455	7.127	P50	23.38	23.57	0.19	0.19 kg
Girls WFA	2557.2500-2648.5625 days	455	7.127	P97	30.69	30.78	0.10	0.10 kg
Girls WFA	2648.5625-2739.8750 days	440	7.376	P3	19.07	18.92	-0.15	0.15 kg
Girls WFA	2648.5625-2739.8750 days	440	7.376	P50	23.66	24.10	0.44	0.44 kg
Girls WFA	2648.5625-2739.8750 days	440	7.376	P97	31.26	31.55	0.28	0.28 kg
Girls WFA	2739.8750-2831.1875 days	466	7.628	P3	19.76	19.32	-0.44	0.44 kg
Girls WFA	2739.8750-2831.1875 days	466	7.628	P50	24.98	24.67	-0.31	0.31 kg
Girls WFA	2739.8750-2831.1875 days	466	7.628	P97	32.38	32.41	0.04	0.04 kg
Girls WFA	2831.1875-2922.5000 days	480	7.877	P3	19.83	19.77	-0.06	0.06 kg
Girls WFA	2831.1875-2922.5000 days	480	7.877	P50	25.40	25.32	-0.08	0.08 kg
Girls WFA	2831.1875-2922.5000 days	480	7.877	P97	32.76	33.41	0.65	0.65 kg
Girls WFA	2922.5000-3013.8125 days	515	8.126	P3	20.07	20.28	0.21	0.21 kg
Girls WFA	2922.5000-3013.8125 days	515	8.126	P50	26.08	26.07	-0.00	0.00 kg
Girls WFA	2922.5000-3013.8125 days	515	8.126	P97	34.38	34.56	0.18	0.18 kg

Model	Age band	*N*	Prediction age (years)	Centile	Empirical	Model-based	Model - empirical	Absolute difference / unit
Girls WFA	3013.8125-3105.1250 days	553	8.375	P3	20.58	20.82	0.24	0.24 kg
Girls WFA	3013.8125-3105.1250 days	553	8.375	P50	26.43	26.88	0.45	0.45 kg
Girls WFA	3013.8125-3105.1250 days	553	8.375	P97	35.90	35.83	-0.08	0.08 kg
Girls WFA	3105.1250-3196.4375 days	588	8.627	P3	21.44	21.33	-0.11	0.11 kg
Girls WFA	3105.1250-3196.4375 days	588	8.627	P50	28.18	27.67	-0.51	0.51 kg
Girls WFA	3105.1250-3196.4375 days	588	8.627	P97	37.53	37.09	-0.44	0.44 kg
Girls WFA	3196.4375-3287.7500 days	564	8.876	P3	21.53	21.77	0.24	0.24 kg
Girls WFA	3196.4375-3287.7500 days	564	8.876	P50	28.34	28.37	0.03	0.03 kg
Girls WFA	3196.4375-3287.7500 days	564	8.876	P97	38.46	38.23	-0.22	0.22 kg
Girls WFA	3287.7500-3379.0625 days	641	9.125	P3	22.21	22.19	-0.02	0.02 kg
Girls WFA	3287.7500-3379.0625 days	641	9.125	P50	29.24	29.04	-0.20	0.20 kg
Girls WFA	3287.7500-3379.0625 days	641	9.125	P97	39.56	39.36	-0.20	0.20 kg
Girls WFA	3379.0625-3470.3750 days	669	9.377	P3	22.59	22.66	0.07	0.07 kg
Girls WFA	3379.0625-3470.3750 days	669	9.377	P50	29.18	29.79	0.61	0.61 kg
Girls WFA	3379.0625-3470.3750 days	669	9.377	P97	39.47	40.61	1.13	1.13 kg
Girls WFA	3470.3750-3561.6875 days	624	9.626	P3	23.59	23.23	-0.36	0.36 kg
Girls WFA	3470.3750-3561.6875 days	624	9.626	P50	30.58	30.67	0.09	0.09 kg
Girls WFA	3470.3750-3561.6875 days	624	9.626	P97	43.53	42.05	-1.48	1.48 kg
Girls WFA	3561.6875-3653.0000 days	643	9.875	P3	23.70	23.88	0.17	0.17 kg
Girls WFA	3561.6875-3653.0000 days	643	9.875	P50	31.61	31.66	0.05	0.05 kg
Girls WFA	3561.6875-3653.0000 days	643	9.875	P97	43.77	43.64	-0.13	0.13 kg
Girls BMIFA	[2.0, 2.5)	2,419	2.250	P3	14.94	14.96	0.02	0.02 kg/m^2
Girls BMIFA	[2.0, 2.5)	2,419	2.250	P50	16.75	16.70	-0.05	0.05 kg/m^2
Girls BMIFA	[2.0, 2.5)	2,419	2.250	P97	19.41	19.40	-0.01	0.01 kg/m^2
Girls BMIFA	[2.5, 3.0)	818	2.750	P3	14.74	14.78	0.04	0.04 kg/m^2
Girls BMIFA	[2.5, 3.0)	818	2.750	P50	16.50	16.55	0.04	0.04 kg/m^2

Model	Age band	*N*	Prediction age (years)	Centile	Empirical	Model-based	Model - empirical	Absolute difference / unit
Girls BMIFA	[2.5, 3.0)	818	2.750	P97	19.41	19.27	-0.14	0.14 kg/m^2
Girls BMIFA	[3.0, 3.5)	1,165	3.250	P3	14.63	14.65	0.01	0.01 kg/m^2
Girls BMIFA	[3.0, 3.5)	1,165	3.250	P50	16.47	16.44	-0.03	0.03 kg/m^2
Girls BMIFA	[3.0, 3.5)	1,165	3.250	P97	19.14	19.20	0.06	0.06 kg/m^2
Girls BMIFA	[3.5, 4.0)	639	3.750	P3	14.48	14.51	0.03	0.03 kg/m^2
Girls BMIFA	[3.5, 4.0)	639	3.750	P50	16.31	16.33	0.01	0.01 kg/m^2
Girls BMIFA	[3.5, 4.0)	639	3.750	P97	18.84	19.15	0.31	0.31 kg/m^2
Girls BMIFA	[4.0, 4.5)	961	4.250	P3	14.41	14.37	-0.04	0.04 kg/m^2
Girls BMIFA	[4.0, 4.5)	961	4.250	P50	16.30	16.23	-0.07	0.07 kg/m^2
Girls BMIFA	[4.0, 4.5)	961	4.250	P97	19.13	19.11	-0.02	0.02 kg/m^2
Girls BMIFA	[4.5, 5.0)	729	4.750	P3	14.22	14.24	0.03	0.03 kg/m^2
Girls BMIFA	[4.5, 5.0)	729	4.750	P50	16.08	16.15	0.06	0.06 kg/m^2
Girls BMIFA	[4.5, 5.0)	729	4.750	P97	18.86	19.12	0.26	0.26 kg/m^2
Girls BMIFA	[5.0, 5.5)	627	5.250	P3	14.18	14.14	-0.04	0.04 kg/m^2
Girls BMIFA	[5.0, 5.5)	627	5.250	P50	16.10	16.09	-0.01	0.01 kg/m^2
Girls BMIFA	[5.0, 5.5)	627	5.250	P97	18.93	19.17	0.24	0.24 kg/m^2
Girls BMIFA	[5.5, 6.0)	659	5.750	P3	14.09	14.05	-0.04	0.04 kg/m^2
Girls BMIFA	[5.5, 6.0)	659	5.750	P50	16.09	16.07	-0.02	0.02 kg/m^2
Girls BMIFA	[5.5, 6.0)	659	5.750	P97	19.35	19.28	-0.07	0.07 kg/m^2
Girls BMIFA	[6.0, 6.5)	1,451	6.250	P3	13.94	13.99	0.05	0.05 kg/m^2
Girls BMIFA	[6.0, 6.5)	1,451	6.250	P50	16.08	16.08	0.00	0.00 kg/m^2
Girls BMIFA	[6.0, 6.5)	1,451	6.250	P97	19.59	19.44	-0.14	0.14 kg/m^2
Girls BMIFA	[6.5, 7.0)	1,383	6.750	P3	13.93	13.95	0.01	0.01 kg/m^2
Girls BMIFA	[6.5, 7.0)	1,383	6.750	P50	16.04	16.12	0.08	0.08 kg/m^2
Girls BMIFA	[6.5, 7.0)	1,383	6.750	P97	19.71	19.67	-0.04	0.04 kg/m^2
Girls BMIFA	[7.0, 7.5)	922	7.250	P3	13.88	13.92	0.04	0.04 kg/m^2
Girls BMIFA	[7.0, 7.5)	922	7.250	P50	16.15	16.20	0.04	0.04 kg/m^2
Girls BMIFA	[7.0, 7.5)	922	7.250	P97	19.78	19.96	0.19	0.19 kg/m^2
Girls BMIFA	[7.5, 8.0)	965	7.750	P3	13.95	13.91	-0.04	0.04 kg/m^2
Girls BMIFA	[7.5, 8.0)	965	7.750	P50	16.30	16.30	-0.00	0.00 kg/m^2
Girls BMIFA	[7.5, 8.0)	965	7.750	P97	20.10	20.32	0.23	0.23 kg/m^2
Girls BMIFA	[8.0, 8.5)	1,089	8.250	P3	13.84	13.91	0.07	0.07 kg/m^2
Girls BMIFA	[8.0, 8.5)	1,089	8.250	P50	16.46	16.43	-0.04	0.04 kg/m^2
Girls BMIFA	[8.0, 8.5)	1,089	8.250	P97	20.72	20.76	0.03	0.03 kg/m^2
Girls BMIFA	[8.5, 9.0)	1,184	8.750	P3	14.04	13.94	-0.10	0.10 kg/m^2
Girls BMIFA	[8.5, 9.0)	1,184	8.750	P50	16.70	16.59	-0.10	0.10 kg/m^2
Girls BMIFA	[8.5, 9.0)	1,184	8.750	P97	21.37	21.26	-0.11	0.11 kg/m^2
Girls BMIFA	[9.0, 9.5)	1,329	9.250	P3	14.06	14.00	-0.06	0.06 kg/m^2
Girls BMIFA	[9.0, 9.5)	1,329	9.250	P50	16.71	16.79	0.09	0.09 kg/m^2

Model	Age band	*N*	Prediction age (years)	Centile	Empirical	Model-based	Model - empirical	Absolute difference / unit
Girls BMIFA	[9.0, 9.5)	1,329	9.250	P97	22.10	21.82	-0.28	0.28 kg/m^2
Girls BMIFA	[9.5, 10.0)	1,294	9.750	P3	14.14	14.09	-0.04	0.04 kg/m^2
Girls BMIFA	[9.5, 10.0)	1,294	9.750	P50	16.89	17.04	0.15	0.15 kg/m^2
Girls BMIFA	[9.5, 10.0)	1,294	9.750	P97	22.67	22.43	-0.23	0.23 kg/m^2
Girls BMIFA	[10.0, 10.5)	1,490	10.250	P3	14.28	14.23	-0.05	0.05 kg/m^2
Girls BMIFA	[10.0, 10.5)	1,490	10.250	P50	17.36	17.33	-0.04	0.04 kg/m^2
Girls BMIFA	[10.0, 10.5)	1,490	10.250	P97	23.34	23.08	-0.26	0.26 kg/m^2
Girls BMIFA	[10.5, 11.0)	1,442	10.750	P3	14.36	14.43	0.07	0.07 kg/m^2
Girls BMIFA	[10.5, 11.0)	1,442	10.750	P50	17.64	17.66	0.02	0.02 kg/m^2
Girls BMIFA	[10.5, 11.0)	1,442	10.750	P97	23.56	23.74	0.18	0.18 kg/m^2
Girls BMIFA	[11.0, 11.5)	1,505	11.250	P3	14.67	14.68	0.01	0.01 kg/m^2
Girls BMIFA	[11.0, 11.5)	1,505	11.250	P50	18.09	18.04	-0.05	0.05 kg/m^2
Girls BMIFA	[11.0, 11.5)	1,505	11.250	P97	24.23	24.39	0.16	0.16 kg/m^2
Girls BMIFA	[11.5, 12.0)	1,400	11.750	P3	15.05	14.98	-0.07	0.07 kg/m^2
Girls BMIFA	[11.5, 12.0)	1,400	11.750	P50	18.42	18.46	0.04	0.04 kg/m^2
Girls BMIFA	[11.5, 12.0)	1,400	11.750	P97	25.23	25.02	-0.21	0.21 kg/m^2
Girls BMIFA	[12.0, 12.5)	1,349	12.250	P3	15.42	15.30	-0.12	0.12 kg/m^2
Girls BMIFA	[12.0, 12.5)	1,349	12.250	P50	18.97	18.89	-0.08	0.08 kg/m^2
Girls BMIFA	[12.0, 12.5)	1,349	12.250	P97	25.92	25.61	-0.31	0.31 kg/m^2
Girls BMIFA	[12.5, 13.0)	1,129	12.750	P3	15.61	15.65	0.03	0.03 kg/m^2
Girls BMIFA	[12.5, 13.0)	1,129	12.750	P50	19.13	19.33	0.20	0.20 kg/m^2
Girls BMIFA	[12.5, 13.0)	1,129	12.750	P97	25.60	26.15	0.56	0.56 kg/m^2
Girls BMIFA	[13.0, 13.5)	881	13.250	P3	15.81	16.00	0.19	0.19 kg/m^2
Girls BMIFA	[13.0, 13.5)	881	13.250	P50	19.62	19.76	0.14	0.14 kg/m^2
Girls BMIFA	[13.0, 13.5)	881	13.250	P97	26.00	26.64	0.64	0.64 kg/m^2
Girls BMIFA	[13.5, 14.0)	731	13.750	P3	16.34	16.35	0.00	0.00 kg/m^2
Girls BMIFA	[13.5, 14.0)	731	13.750	P50	20.03	20.17	0.14	0.14 kg/m^2
Girls BMIFA	[13.5, 14.0)	731	13.750	P97	27.08	27.07	-0.01	0.01 kg/m^2
Girls BMIFA	[14.0, 14.5)	541	14.250	P3	16.64	16.69	0.05	0.05 kg/m^2
Girls BMIFA	[14.0, 14.5)	541	14.250	P50	20.80	20.55	-0.25	0.25 kg/m^2
Girls BMIFA	[14.0, 14.5)	541	14.250	P97	26.91	27.44	0.53	0.53 kg/m^2
Girls BMIFA	[14.5, 15.0)	419	14.750	P3	17.09	17.01	-0.09	0.09 kg/m^2
Girls BMIFA	[14.5, 15.0)	419	14.750	P50	20.94	20.91	-0.03	0.03 kg/m^2
Girls BMIFA	[14.5, 15.0)	419	14.750	P97	27.69	27.74	0.05	0.05 kg/m^2
Girls BMIFA	[15.0, 15.5)	440	15.250	P3	17.42	17.31	-0.11	0.11 kg/m^2
Girls BMIFA	[15.0, 15.5)	440	15.250	P50	21.15	21.23	0.08	0.08 kg/m^2
Girls BMIFA	[15.0, 15.5)	440	15.250	P97	27.75	28.00	0.24	0.24 kg/m^2
Girls BMIFA	[15.5, 16.0)	506	15.750	P3	17.65	17.59	-0.06	0.06 kg/m^2
Girls BMIFA	[15.5, 16.0)	506	15.750	P50	21.50	21.53	0.03	0.03 kg/m^2

Model	Age band	*N*	Prediction age (years)	Centile	Empirical	Model-based	Model - empirical	Absolute difference / unit
Girls BMIFA	[15.5, 16.0)	506	15.750	P97	29.04	28.21	-0.83	0.83 kg/m^2
Girls BMIFA	[16.0, 16.5)	682	16.250	P3	18.25	17.85	-0.40	0.40 kg/m^2
Girls BMIFA	[16.0, 16.5)	682	16.250	P50	22.08	21.79	-0.29	0.29 kg/m^2
Girls BMIFA	[16.0, 16.5)	682	16.250	P97	28.77	28.38	-0.39	0.39 kg/m^2
Girls BMIFA	[16.5, 17.0)	929	16.750	P3	18.12	18.08	-0.03	0.03 kg/m^2
Girls BMIFA	[16.5, 17.0)	929	16.750	P50	21.85	22.03	0.18	0.18 kg/m^2
Girls BMIFA	[16.5, 17.0)	929	16.750	P97	28.22	28.53	0.31	0.31 kg/m^2
Girls BMIFA	[17.0, 17.5)	1,134	17.250	P3	18.35	18.30	-0.05	0.05 kg/m^2
Girls BMIFA	[17.0, 17.5)	1,134	17.250	P50	22.16	22.25	0.09	0.09 kg/m^2
Girls BMIFA	[17.0, 17.5)	1,134	17.250	P97	28.10	28.66	0.56	0.56 kg/m^2
Girls BMIFA	[17.5, 18.0)	1,452	17.750	P3	18.20	18.49	0.29	0.29 kg/m^2
Girls BMIFA	[17.5, 18.0)	1,452	17.750	P50	22.58	22.44	-0.14	0.14 kg/m^2
Girls BMIFA	[17.5, 18.0)	1,452	17.750	P97	28.75	28.76	0.01	0.01 kg/m^2

Note. HFA and BMIFA use half-year age bands; WFA uses three-month age bands. Comparisons describe calibration within the analytic data and are not external clinical validation.

Table S23. Absolute differences between Jordanian and WHO selected centiles by age, sex, and indicator.

Model	Target age	Matched age (days / years)	Centile	Jordanian	WHO	Jordanian - WHO	Absolute difference / unit	WHO source
Boys HFA	2	731 / 2.001	P3	81.48	81.38	0.10	0.10 cm	WHO_2006_Daily_0_5
Boys HFA	2	731 / 2.001	P15	84.02	83.96	0.05	0.05 cm	WHO_2006_Daily_0_5
Boys HFA	2	731 / 2.001	P50	87.32	87.13	0.19	0.19 cm	WHO_2006_Daily_0_5
Boys HFA	2	731 / 2.001	P85	90.86	90.30	0.56	0.56 cm	WHO_2006_Daily_0_5
Boys HFA	2	731 / 2.001	P97	93.94	92.88	1.06	1.06 cm	WHO_2006_Daily_0_5
Boys HFA	5	1826 / 4.999	P3	101.39	101.24	0.15	0.15 cm	WHO_2006_Daily_0_5
Boys HFA	5	1826 / 4.999	P15	104.91	105.16	-0.24	0.24 cm	WHO_2006_Daily_0_5
Boys HFA	5	1826 / 4.999	P50	109.44	109.96	-0.52	0.52 cm	WHO_2006_Daily_0_5
Boys HFA	5	1826 / 4.999	P85	114.21	114.76	-0.55	0.55 cm	WHO_2006_Daily_0_5
Boys HFA	5	1826 / 4.999	P97	118.28	118.67	-0.39	0.39 cm	WHO_2006_Daily_0_5
Boys HFA	10	3652 / 9.999	P3	126.12	125.79	0.34	0.34 cm	WHO_2007_Monthly_5_19
Boys HFA	10	3652 / 9.999	P15	130.55	131.17	-0.62	0.62 cm	WHO_2007_Monthly_5_19
Boys HFA	10	3652 / 9.999	P50	136.12	137.77	-1.65	1.65 cm	WHO_2007_Monthly_5_19
Boys HFA	10	3652 / 9.999	P85	141.84	144.38	-2.54	2.54 cm	WHO_2007_Monthly_5_19
Boys HFA	10	3652 / 9.999	P97	146.62	149.76	-3.14	3.14 cm	WHO_2007_Monthly_5_19
Boys HFA	15	5479 / 15.001	P3	153.44	154.28	-0.85	0.85 cm	WHO_2007_Monthly_5_19
Boys HFA	15	5479 / 15.001	P15	158.94	160.87	-1.94	1.94 cm	WHO_2007_Monthly_5_19
Boys HFA	15	5479 / 15.001	P50	165.79	168.96	-3.18	3.18 cm	WHO_2007_Monthly_5_19
Boys HFA	15	5479 / 15.001	P85	172.75	177.05	-4.30	4.30 cm	WHO_2007_Monthly_5_19
Boys HFA	15	5479 / 15.001	P97	178.50	183.64	-5.14	5.14 cm	WHO_2007_Monthly_5_19
Boys HFA	18	6574 / 17.999	P3	164.92	162.09	2.83	2.83 cm	WHO_2007_Monthly_5_19
Boys HFA	18	6574 / 17.999	P15	169.12	168.40	0.72	0.72 cm	WHO_2007_Monthly_5_19
Boys HFA	18	6574 / 17.999	P50	174.33	176.14	-1.82	1.82 cm	WHO_2007_Monthly_5_19
Boys HFA	18	6574 / 17.999	P85	179.57	183.89	-4.31	4.31 cm	WHO_2007_Monthly_5_19
Boys HFA	18	6574 / 17.999	P97	183.88	190.19	-6.32	6.32 cm	WHO_2007_Monthly_5_19
Boys WFA	2	731 / 2.001	P3	10.73	9.81	0.92	0.92 kg	WHO_2006_Daily_0_5
Boys WFA	2	731 / 2.001	P15	11.64	10.80	0.84	0.84 kg	WHO_2006_Daily_0_5
Boys WFA	2	731 / 2.001	P50	12.92	12.15	0.77	0.77 kg	WHO_2006_Daily_0_5
Boys WFA	2	731 / 2.001	P85	14.48	13.68	0.80	0.80 kg	WHO_2006_Daily_0_5
Boys WFA	2	731 / 2.001	P97	16.08	15.07	1.00	1.00 kg	WHO_2006_Daily_0_5
Boys WFA	5	1826 / 4.999	P3	15.83	14.29	1.55	1.55 kg	WHO_2006_Daily_0_5
Boys WFA	5	1826 / 4.999	P15	17.33	15.96	1.37	1.37 kg	WHO_2006_Daily_0_5
Boys WFA	5	1826 / 4.999	P50	19.49	18.34	1.15	1.15 kg	WHO_2006_Daily_0_5
Boys WFA	5	1826 / 4.999	P85	22.17	21.12	1.05	1.05 kg	WHO_2006_Daily_0_5
Boys WFA	5	1826 / 4.999	P97	24.98	23.76	1.22	1.22 kg	WHO_2006_Daily_0_5
Boys WFA	10	3652 / 9.999	P3	24.41	23.58	0.84	0.84 kg	WHO_2007_Monthly_5_10
Boys WFA	10	3652 / 9.999	P15	27.56	26.55	1.01	1.01 kg	WHO_2007_Monthly_5_10
Boys WFA	10	3652 / 9.999	P50	32.38	31.15	1.23	1.23 kg	WHO_2007_Monthly_5_10

Model	Target age	Matched age (days / years)	Centile	Jordanian	WHO	Jordanian - WHO	Absolute difference / unit	WHO source
Boys WFA	10	3652 / 9.999	P85	38.96	37.28	1.69	1.69 kg	WHO_2007_Monthly_5_10
Boys WFA	10	3652 / 9.999	P97	46.60	43.93	2.67	2.67 kg	WHO_2007_Monthly_5_10
Boys BMIFA	2	731 / 2.001	P3	15.05	13.92	1.12	1.12 kg/m^2	WHO_2006_Daily_0_5
Boys BMIFA	2	731 / 2.001	P15	15.76	14.81	0.95	0.95 kg/m^2	WHO_2006_Daily_0_5
Boys BMIFA	2	731 / 2.001	P50	16.88	16.02	0.86	0.86 kg/m^2	WHO_2006_Daily_0_5
Boys BMIFA	2	731 / 2.001	P85	18.37	17.40	0.97	0.97 kg/m^2	WHO_2006_Daily_0_5
Boys BMIFA	2	731 / 2.001	P97	20.04	18.68	1.36	1.36 kg/m^2	WHO_2006_Daily_0_5
Boys BMIFA	5	1826 / 4.999	P3	14.20	13.01	1.19	1.19 kg/m^2	WHO_2006_Daily_0_5
Boys BMIFA	5	1826 / 4.999	P15	14.99	13.92	1.08	1.08 kg/m^2	WHO_2006_Daily_0_5
Boys BMIFA	5	1826 / 4.999	P50	16.20	15.19	1.00	1.00 kg/m^2	WHO_2006_Daily_0_5
Boys BMIFA	5	1826 / 4.999	P85	17.82	16.67	1.15	1.15 kg/m^2	WHO_2006_Daily_0_5
Boys BMIFA	5	1826 / 4.999	P97	19.75	18.07	1.68	1.68 kg/m^2	WHO_2006_Daily_0_5
Boys BMIFA	10	3652 / 9.999	P3	14.15	13.86	0.29	0.29 kg/m^2	WHO_2007_Monthly_5_19
Boys BMIFA	10	3652 / 9.999	P15	15.34	14.87	0.47	0.47 kg/m^2	WHO_2007_Monthly_5_19
Boys BMIFA	10	3652 / 9.999	P50	17.33	16.44	0.89	0.89 kg/m^2	WHO_2007_Monthly_5_19
Boys BMIFA	10	3652 / 9.999	P85	20.28	18.57	1.71	1.71 kg/m^2	WHO_2007_Monthly_5_19
Boys BMIFA	10	3652 / 9.999	P97	23.92	20.98	2.94	2.94 kg/m^2	WHO_2007_Monthly_5_19
Boys BMIFA	15	5479 / 15.001	P3	16.45	16.19	0.27	0.27 kg/m^2	WHO_2007_Monthly_5_19
Boys BMIFA	15	5479 / 15.001	P15	18.02	17.58	0.44	0.44 kg/m^2	WHO_2007_Monthly_5_19
Boys BMIFA	15	5479 / 15.001	P50	20.74	19.77	0.97	0.97 kg/m^2	WHO_2007_Monthly_5_19
Boys BMIFA	15	5479 / 15.001	P85	24.57	22.81	1.76	1.76 kg/m^2	WHO_2007_Monthly_5_19
Boys BMIFA	15	5479 / 15.001	P97	28.61	26.35	2.26	2.26 kg/m^2	WHO_2007_Monthly_5_19
Boys BMIFA	18	6574 / 17.999	P3	18.22	17.50	0.73	0.73 kg/m^2	WHO_2007_Monthly_5_19
Boys BMIFA	18	6574 / 17.999	P15	19.85	19.16	0.68	0.68 kg/m^2	WHO_2007_Monthly_5_19
Boys BMIFA	18	6574 / 17.999	P50	22.65	21.71	0.94	0.94 kg/m^2	WHO_2007_Monthly_5_19
Boys BMIFA	18	6574 / 17.999	P85	26.31	25.05	1.26	1.26 kg/m^2	WHO_2007_Monthly_5_19
Boys BMIFA	18	6574 / 17.999	P97	29.68	28.65	1.04	1.04 kg/m^2	WHO_2007_Monthly_5_19
Girls HFA	2	731 / 2.001	P3	79.50	79.66	-0.16	0.16 cm	WHO_2006_Daily_0_5
Girls HFA	2	731 / 2.001	P15	82.50	82.39	0.11	0.11 cm	WHO_2006_Daily_0_5
Girls HFA	2	731 / 2.001	P50	85.96	85.73	0.23	0.23 cm	WHO_2006_Daily_0_5
Girls HFA	2	731 / 2.001	P85	89.44	89.07	0.37	0.37 cm	WHO_2006_Daily_0_5
Girls HFA	2	731 / 2.001	P97	92.46	91.80	0.66	0.66 cm	WHO_2006_Daily_0_5
Girls HFA	5	1826 / 4.999	P3	99.51	100.47	-0.96	0.96 cm	WHO_2006_Daily_0_5
Girls HFA	5	1826 / 4.999	P15	103.39	104.49	-1.10	1.10 cm	WHO_2006_Daily_0_5
Girls HFA	5	1826 / 4.999	P50	108.43	109.42	-0.99	0.99 cm	WHO_2006_Daily_0_5
Girls HFA	5	1826 / 4.999	P85	113.73	114.35	-0.61	0.61 cm	WHO_2006_Daily_0_5
Girls HFA	5	1826 / 4.999	P97	118.24	118.36	-0.12	0.12 cm	WHO_2006_Daily_0_5
Girls HFA	10	3652 / 9.999	P3	123.63	126.60	-2.97	2.97 cm	WHO_2007_Monthly_5_19
Girls HFA	10	3652 / 9.999	P15	129.32	132.00	-2.68	2.68 cm	WHO_2007_Monthly_5_19

Model	Target age	Matched age (days / years)	Centile	Jordanian	WHO	Jordanian - WHO	Absolute difference / unit	WHO source
Girls HFA	10	3652 / 9.999	P50	136.61	138.63	-2.01	2.01 cm	WHO_2007_Monthly_5_19
Girls HFA	10	3652 / 9.999	P85	144.16	145.26	-1.09	1.09 cm	WHO_2007_Monthly_5_19
Girls HFA	10	3652 / 9.999	P97	150.43	150.66	-0.23	0.23 cm	WHO_2007_Monthly_5_19
Girls HFA	15	5479 / 15.001	P3	146.84	148.73	-1.89	1.89 cm	WHO_2007_Monthly_5_19
Girls HFA	15	5479 / 15.001	P15	151.95	154.54	-2.59	2.59 cm	WHO_2007_Monthly_5_19
Girls HFA	15	5479 / 15.001	P50	158.15	161.67	-3.52	3.52 cm	WHO_2007_Monthly_5_19
Girls HFA	15	5479 / 15.001	P85	164.31	168.80	-4.49	4.49 cm	WHO_2007_Monthly_5_19
Girls HFA	15	5479 / 15.001	P97	169.34	174.61	-5.27	5.27 cm	WHO_2007_Monthly_5_19
Girls HFA	18	6574 / 17.999	P3	148.41	150.63	-2.22	2.22 cm	WHO_2007_Monthly_5_19
Girls HFA	18	6574 / 17.999	P15	153.18	156.21	-3.03	3.03 cm	WHO_2007_Monthly_5_19
Girls HFA	18	6574 / 17.999	P50	158.83	163.06	-4.23	4.23 cm	WHO_2007_Monthly_5_19
Girls HFA	18	6574 / 17.999	P85	164.37	169.91	-5.54	5.54 cm	WHO_2007_Monthly_5_19
Girls HFA	18	6574 / 17.999	P97	168.89	175.49	-6.60	6.60 cm	WHO_2007_Monthly_5_19
Girls WFA	2	731 / 2.001	P3	10.23	9.16	1.06	1.06 kg	WHO_2006_Daily_0_5
Girls WFA	2	731 / 2.001	P15	11.13	10.12	1.01	1.01 kg	WHO_2006_Daily_0_5
Girls WFA	2	731 / 2.001	P50	12.40	11.48	0.92	0.92 kg	WHO_2006_Daily_0_5
Girls WFA	2	731 / 2.001	P85	13.89	13.09	0.80	0.80 kg	WHO_2006_Daily_0_5
Girls WFA	2	731 / 2.001	P97	15.29	14.62	0.68	0.68 kg	WHO_2006_Daily_0_5
Girls WFA	5	1826 / 4.999	P3	15.32	13.96	1.36	1.36 kg	WHO_2006_Daily_0_5
Girls WFA	5	1826 / 4.999	P15	16.85	15.69	1.17	1.17 kg	WHO_2006_Daily_0_5
Girls WFA	5	1826 / 4.999	P50	19.05	18.22	0.83	0.83 kg	WHO_2006_Daily_0_5
Girls WFA	5	1826 / 4.999	P85	21.68	21.33	0.35	0.35 kg	WHO_2006_Daily_0_5
Girls WFA	5	1826 / 4.999	P97	24.23	24.43	-0.20	0.20 kg	WHO_2006_Daily_0_5
Girls WFA	10	3652 / 9.999	P3	24.21	23.67	0.54	0.54 kg	WHO_2007_Monthly_5_10
Girls WFA	10	3652 / 9.999	P15	27.39	26.87	0.51	0.51 kg	WHO_2007_Monthly_5_10
Girls WFA	10	3652 / 9.999	P50	32.17	31.85	0.31	0.31 kg	WHO_2007_Monthly_5_10
Girls WFA	10	3652 / 9.999	P85	38.23	38.49	-0.25	0.25 kg	WHO_2007_Monthly_5_10
Girls WFA	10	3652 / 9.999	P97	44.46	45.69	-1.23	1.23 kg	WHO_2007_Monthly_5_10
Girls BMIFA	2	731 / 2.001	P3	15.07	13.47	1.60	1.60 kg/m^2	WHO_2006_Daily_0_5
Girls BMIFA	2	731 / 2.001	P15	15.78	14.40	1.37	1.37 kg/m^2	WHO_2006_Daily_0_5
Girls BMIFA	2	731 / 2.001	P50	16.81	15.69	1.13	1.13 kg/m^2	WHO_2006_Daily_0_5
Girls BMIFA	2	731 / 2.001	P85	18.13	17.16	0.96	0.96 kg/m^2	WHO_2006_Daily_0_5
Girls BMIFA	2	731 / 2.001	P97	19.51	18.53	0.97	0.97 kg/m^2	WHO_2006_Daily_0_5
Girls BMIFA	5	1826 / 4.999	P3	14.19	12.82	1.37	1.37 kg/m^2	WHO_2006_Daily_0_5
Girls BMIFA	5	1826 / 4.999	P15	14.96	13.84	1.12	1.12 kg/m^2	WHO_2006_Daily_0_5
Girls BMIFA	5	1826 / 4.999	P50	16.11	15.28	0.84	0.84 kg/m^2	WHO_2006_Daily_0_5
Girls BMIFA	5	1826 / 4.999	P85	17.58	16.96	0.63	0.63 kg/m^2	WHO_2006_Daily_0_5
Girls BMIFA	5	1826 / 4.999	P97	19.14	18.55	0.58	0.58 kg/m^2	WHO_2006_Daily_0_5
Girls BMIFA	10	3652 / 9.999	P3	14.16	13.62	0.54	0.54 kg/m^2	WHO_2007_Monthly_5_19

Model	Target age	Matched age (days / years)	Centile	Jordanian	WHO	Jordanian - WHO	Absolute difference / unit	WHO source
Girls BMIFA	10	3652 / 9.999	P15	15.33	14.78	0.55	0.55 kg/m^2	WHO_2007_Monthly_5_19
Girls BMIFA	10	3652 / 9.999	P50	17.18	16.61	0.56	0.56 kg/m^2	WHO_2007_Monthly_5_19
Girls BMIFA	10	3652 / 9.999	P85	19.74	19.14	0.60	0.60 kg/m^2	WHO_2007_Monthly_5_19
Girls BMIFA	10	3652 / 9.999	P97	22.75	22.06	0.69	0.69 kg/m^2	WHO_2007_Monthly_5_19
Girls BMIFA	15	5479 / 15.001	P3	17.16	16.07	1.09	1.09 kg/m^2	WHO_2007_Monthly_5_19
Girls BMIFA	15	5479 / 15.001	P15	18.69	17.69	1.01	1.01 kg/m^2	WHO_2007_Monthly_5_19
Girls BMIFA	15	5479 / 15.001	P50	21.07	20.21	0.86	0.86 kg/m^2	WHO_2007_Monthly_5_19
Girls BMIFA	15	5479 / 15.001	P85	24.28	23.66	0.63	0.63 kg/m^2	WHO_2007_Monthly_5_19
Girls BMIFA	15	5479 / 15.001	P97	27.88	27.56	0.32	0.32 kg/m^2	WHO_2007_Monthly_5_19
Girls BMIFA	18	6574 / 17.999	P3	18.58	16.68	1.90	1.90 kg/m^2	WHO_2007_Monthly_5_19
Girls BMIFA	18	6574 / 17.999	P15	20.15	18.48	1.67	1.67 kg/m^2	WHO_2007_Monthly_5_19
Girls BMIFA	18	6574 / 17.999	P50	22.53	21.26	1.27	1.27 kg/m^2	WHO_2007_Monthly_5_19
Girls BMIFA	18	6574 / 17.999	P85	25.58	24.92	0.66	0.66 kg/m^2	WHO_2007_Monthly_5_19
Girls BMIFA	18	6574 / 17.999	P97	28.81	28.87	-0.06	0.06 kg/m^2	WHO_2007_Monthly_5_19

Note. Differences are descriptive and do not establish diagnostic superiority, interchangeability, or clinical validity. WHO 2006 daily values were used through day 1856; from day 1857 onward, WHO 2007 monthly LMS values were linearly interpolated using age in days/30.4375. Confidence intervals, reclassification metrics, concordance coefficients, and Jordan-specific clinical-outcome validation were unavailable and are not reported.

Figure S1. Flow from eligible Hakeem records through proportionate stratified sampling and analytical cleaning to the six sex-specific model domains.

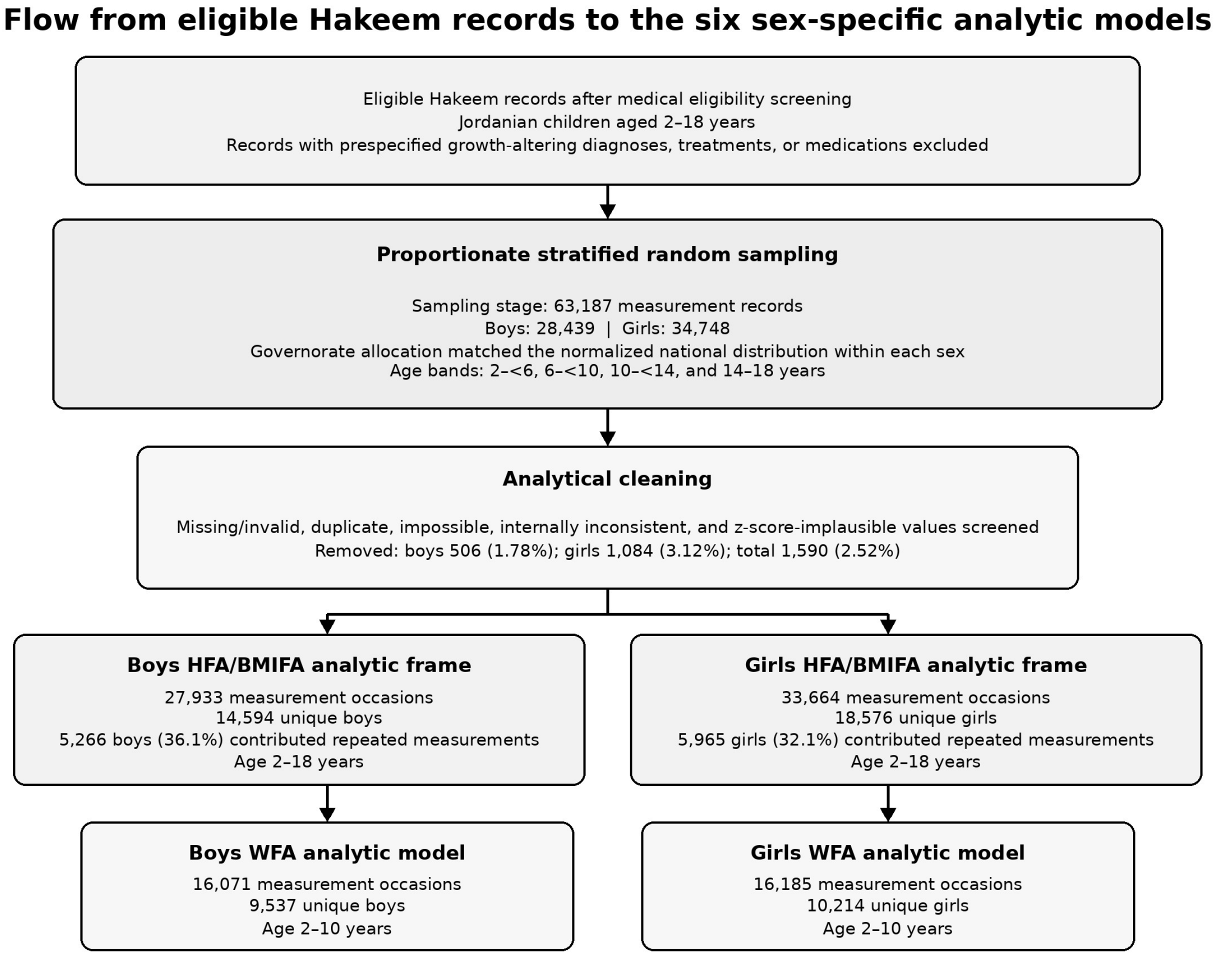

Distinct repeated measurement occasions were retained and modelled as independent attained-growth observations. Outcome-specific denominators must not be summed.

Figure S2. Worm plots for the six selected growth-reference models.

A. Boys: Height-for-age

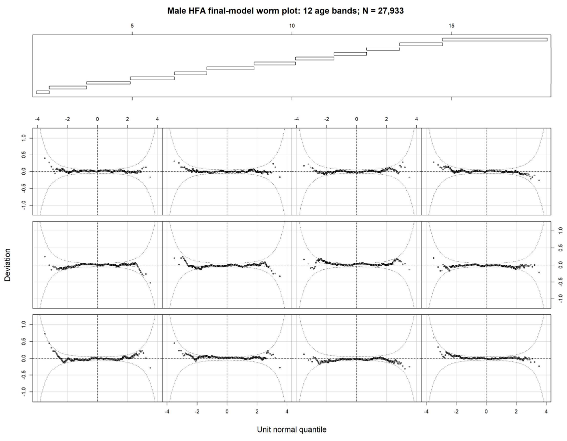

B. Boys: Weight-for-age

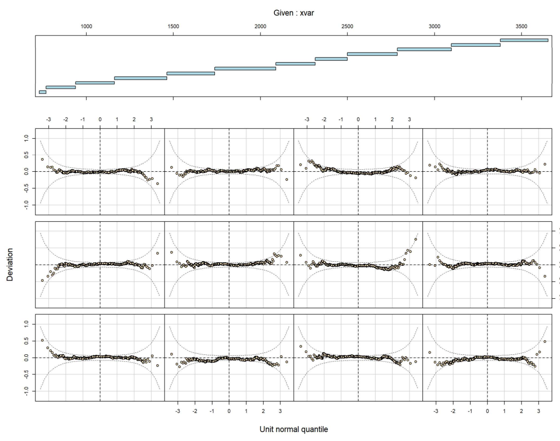

C. Boys: BMI-for-age

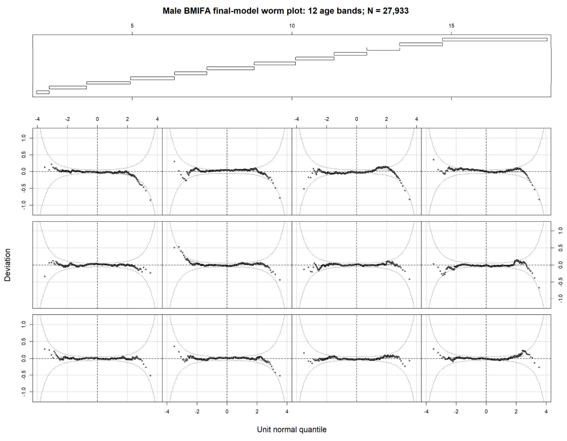

D. Girls: Height-for-age

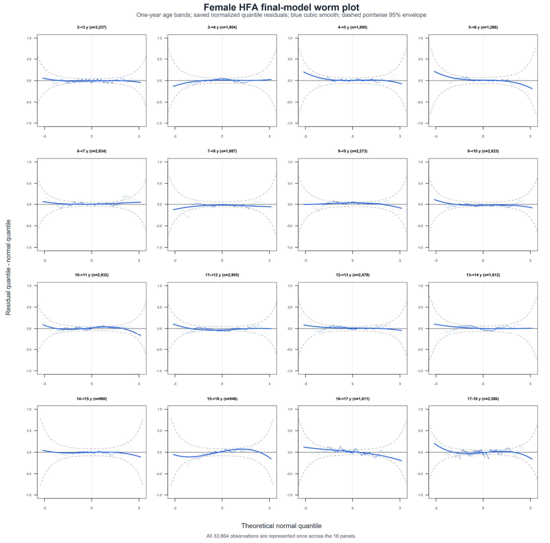

E. Girls: Weight-for-age

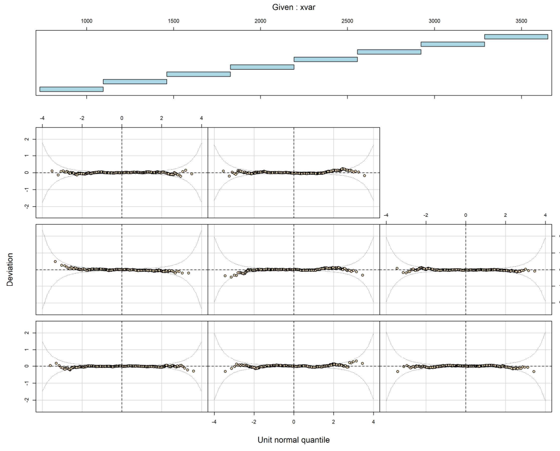

F. Girls: BMI-for-age

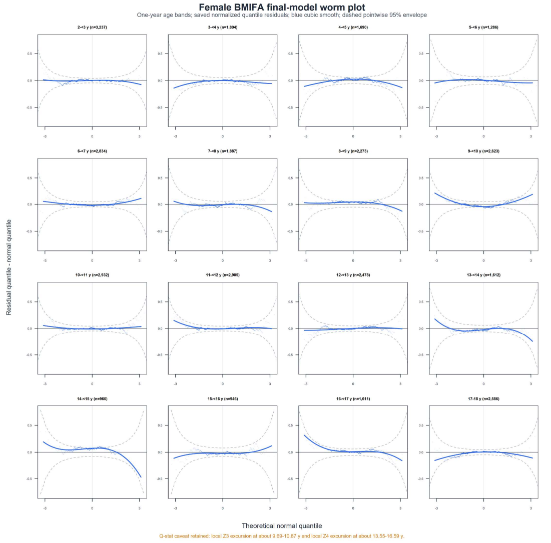

Figure S3. Residual distributions for the six selected growth-reference models.

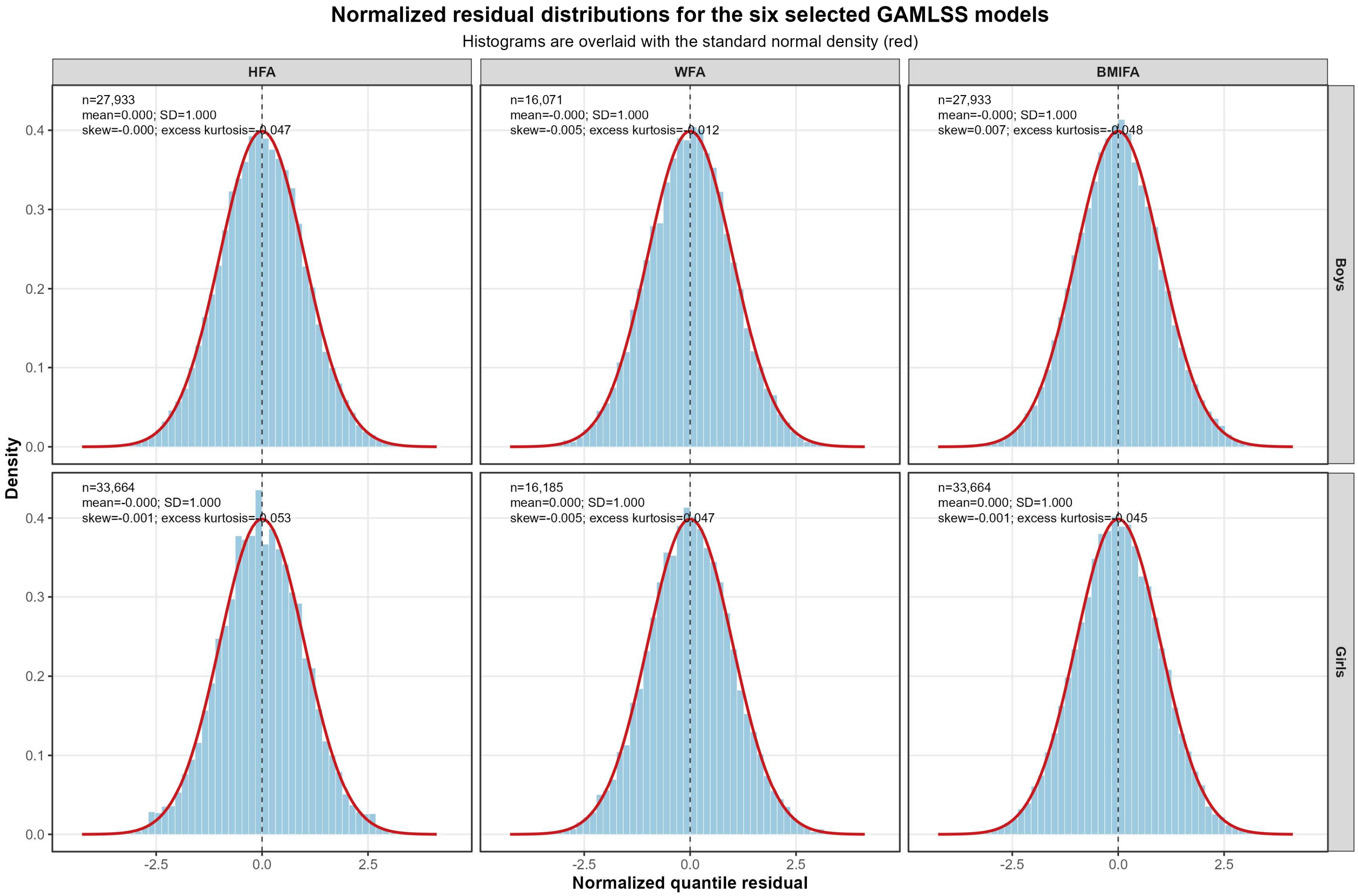

Supplement: Supplementary file 1 [file healthcare-14-02898-s001.zip › healthcare-4413951-supplementary.pdf]
